# Intramolecular Singlet Fission in Individual Graphene Nanoribbons—Competition with a Charge Transfer

**DOI:** 10.1021/jacs.4c18051

**Published:** 2025-03-19

**Authors:** Phillip M. Greißel, Giovanni M. Beneventi, René Weiß, Anna-Sophie Wollny, Rajeev K. Dubey, Manuel Melle-Franco, Timothy Clark, Aurelio Mateo-Alonso, Dirk M. Guldi

**Affiliations:** † Department of Chemistry and Pharmacy and Interdisciplinary Center for Molecular Materials (ICMM), Friedrich-Alexander-University Erlangen-Nuremberg, Egerlandstraße 3, 91058 Erlangen, Germany; ‡ POLYMAT, University of the Basque Country, Avenida de Tolosa 72, 20018 Donostia-San Sebastian, Spain; § CICECO - Aveiro Institute of Materials, Department of Chemistry, University of Aveiro, 3810-193 Aveiro, Portugal; ∥ Department of Chemistry and Pharmacy and Computer-Chemie-Center (CCC), Friedrich-Alexander-University Erlangen-Nuremberg, Nägelsbachstraße 25, 91052 Erlangen, Germany; ⊥ Ikerbasque, Basque Foundation for Science, 48009 Bilbao, Spain

## Abstract

Graphene nanoribbons (NRs) constitute a versatile platform for developing novel materials, where their structure governs their optical, electronic, and magnetic properties while also shaping their excited-state dynamics. Here, we investigate a set of three twisted N-doped molecular NRs of increasing length, obtained by linearly fusing perylene diimide to pyrene and pyrazino- or thiadiazolo-quinoxaline residues. By employing various temperature-dependent time-resolved spectroscopy techniques, we reveal how the flexible twisted NR geometry promotes the formation of a mixed electronic state with varying contributions from locally excited and charge-transfer (CT) states. The fate of this mixed state is highly sensitive to the molecular geometry, length, and solvent polarity. For the shortest NR, intersystem crossing dominates the deactivation pathway, efficiently generating triplets in low-polarity solvents. In contrast, for the extended NRs, intramolecular singlet fission (SF) takes place within a single nanoribbon. This is enabled by enhanced superexchange coupling due to a pronounced push–pull nature and the existence of multiple localized π-electron states caused by heteroatom doping, thereby circumventing the need for dimeric interactions typically associated with conventional SF systems. In higher-polarity environments, evidence of a (diabatic) CT state emerges. These findings underscore the intricate relationship between geometry, energy levels, and excited-state dynamics in twisted N-doped NRs.

## Graphical abstract

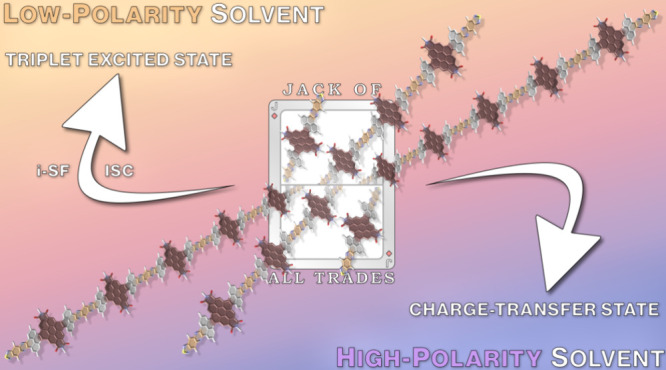

## Introduction

Conventional single-junction solar cells are subject to intrinsic loss processes restricting efficiencies to a thermodynamic maximum of about 33%.^1^ Most notable are thermalization losses, that is, excess photon energy—surpassing the band gap of the material—being lost in the form of heat.^2^ One strategy to overcome such thermalization losses is down-converting high-energy photons, thereby increasing the solar energy conversion efficiencies to roughly 44%.^3^

In organic materials, such a phenomenon is known as singlet fission (SF). SF requires at least two interacting chromophores with triplet energies roughly half that of the singlet energy, as seen in acenes and rylenes.^4–9^ If this fundamental SF energy requirement is met, a singlet excited state ^1^(S_1_S_0_) that spans across both chromophores converts into two independent triplet excited states (T_1_ + T_1_) each residing on one chromophore.^8,10^ The process is best described by the simplified model given in eq 1:^10,11^

(1S1S0)⇄(1T1T1)⇄(MT1···T1)⇄(T1+T1) 1

This conversion proceeds via two intermediate triplet pairs. The initial multiexcitonic correlated triplet pair state ^1^(T_1_T_1_) is strongly coupled and, thus, has pure-spin (singlet) character, rendering SF spin-allowed.^12^ The subsequent triplet pair state ^M^(T_1_···T_1_) exhibits weaker electronic couplings, which may enable spin evolution or mixing, resulting in the loss of its pure-spin singlet nature. Structural fluctuations, as well as spatial separations, allow for modulating the intertriplet exchange couplings, thereby giving rise to spin evolution from ^1^(T_1_T_1_) to ^5^(T_1_T_1_).^7,13–17^

The strength of the electronic couplings governs the SF dynamics, efficiencies, and the mechanism by which it proceeds. In solids, molecular packing is crucial, while in solution, topology and linker electronics play key roles.^4,18–26^ Weakly coupled systems are subject to indirect SF via a charge-transfer (CT) state that mediates superexchange between ^1^(S_1_S_0_) and ^1^(T_1_T_1_).^20,27–30^ In contrast, strongly coupled systems allow for a direct mechanism as couplings are sufficiently large to support a concerted two-electron process.^7,31,32^ However, excessive coupling can lead to low-lying CT or excimer trap states, especially in polar environments.^19,33^ A third scenario arises when ^1^(S_1_S_0_), CT, and ^1^(T_1_T_1_) are close in energy, leading to a coherent SF mechanism, where the electronic state is a dynamic mix of all three contributions.^6,13,19,27,34–37^ Such CT mixing may also lower the ^1^(T_1_T_1_) energies, enabling SF in otherwise unfavorable systems.^19,27^ In other words, the presence of CT is fundamental in SF, especially when a pronounced CT character characterizes the excited state as seen, for example, in ‘push–pull’ oligomers.^38,39^

While diabatic CT formation can hinder SF, it can be harnessed in other applications.^40–42^ For instance, efficient CT formation across large electron–hole separations modulates the ^1^CT-^3^CT gap, which may drive efficient intersystem crossing (ISC) as well as reverse ISC via second-order spin–vibronic coupling,^43^ a principle exploited in high-performance thermally activated delayed fluorescence materials.^44–47^

All this illustrates that a fundamental understanding of the structure–property relationship in multichromophoric architectures is key, particularly for manipulating mixed electronic states through chromophore design and environmental tuning. However, the prediction of such effects toward desired outcomes, such as SF, remains challenging. On top of that, many SF materials suffer from limited stability and processability.^27^ These issues pose a challenge in the development of practical applications, for which reason alternative SF materials are sought, ideally with triplet energies high enough to sensitize common bulk semiconductors such as the 1.1 eV for silicon.^48–50^

Graphene nanoribbons (NRs) are strips or 1D cut outs of graphene^51–55^ (Scheme 1a) with highly tunable electronic, optical, and magnetic properties that are dominated by structural variables, such as edge structure, width, length, and heteroatom-doping, among others.^52,56,57^ The edge structure is a key variable that controls the metallicity of the NRs, as the boundary conditions imposed by the different edge types have a direct impact on the delocalization of π electrons. For example, zigzag edges give rise to metallic NRs with highly delocalized π-electron distributions, whereas armchair, cove or fjord edges generally give rise to semiconducting NRs with localized π-electron distributions.^52,55,58–73^ The length of the NRs is a critical variable that directly influences their properties. With increasing NR lengths, the band gap decreases until saturation,^56,63,72^ a topological phase transition can occur,^74^ molar absorptivity rises,^56,63,72^ and phonon energies decline.^75^ Despite these effects, the impact of length on NR properties remains largely underexplored.

**Scheme 1:**
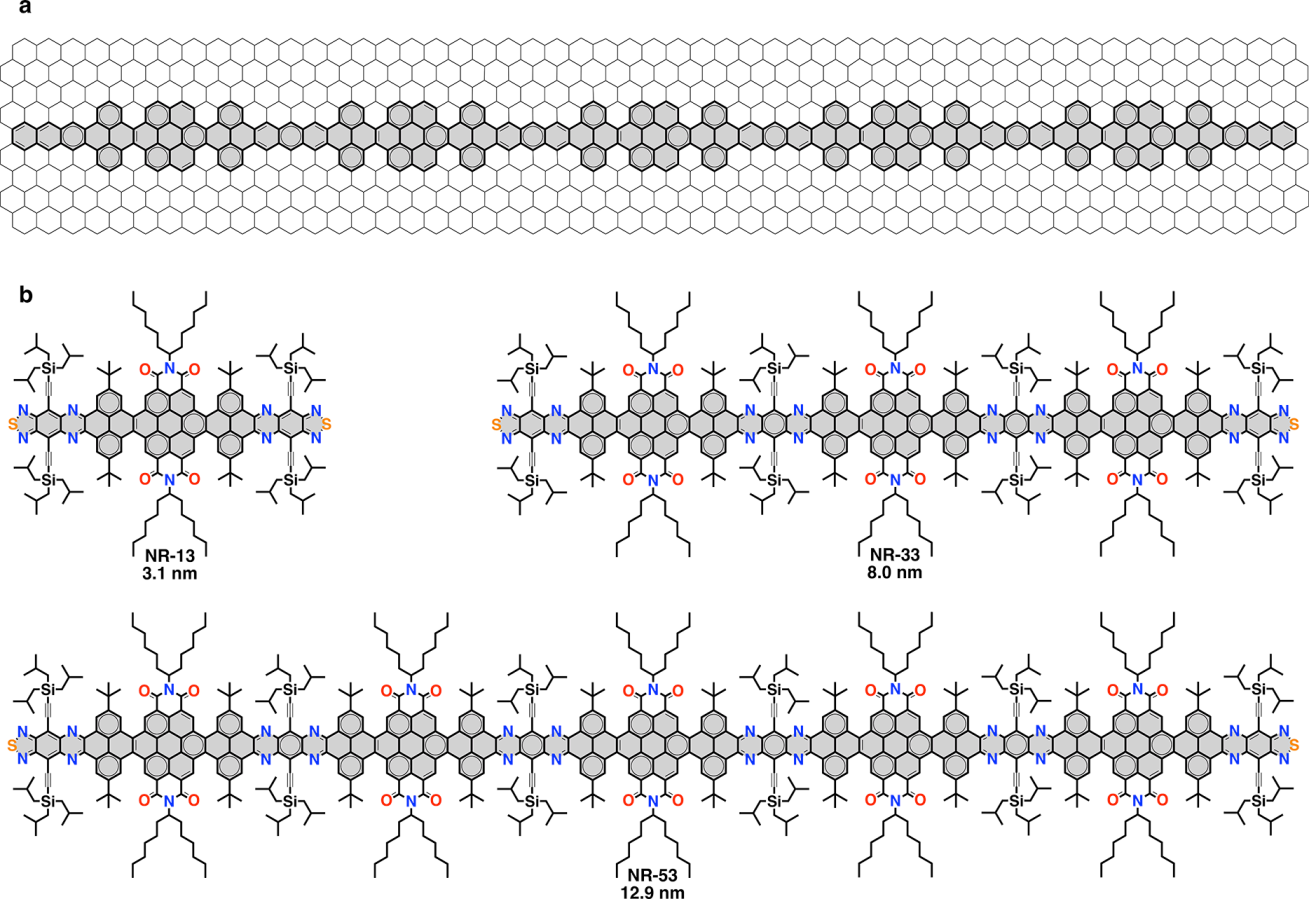
Investigated Nanoribbons^a^

a (a) General structure of graphene nanoribbons. (b) Chemical structures of **NR-13**, **NR-33**, and **NR-53**.

Here, we show that individual semiconducting molecular N-doped NRs can generate triplets through intramolecular SF (*i*-SF) without the need to couple two or more NRs. This generation of multiple excitons within the same NR is possible because semiconducting NRs possess multiple localized π-electron states and can behave as multichromophoric systems at the single NR level. This has been demonstrated by studying a set of semiconducting molecular NRs with zigzag-cape-cove edges and increasing lengths, namely **NR-13** (3.1 nm), **NR-33** (8.0 nm) and **NR-53** (12.9 nm) (Scheme 1b). Employing transient electronic (visible and near-infrared) and vibrational (mid-infrared) absorption as well as emission spectroscopy at various temperatures, we uncover how the admixing of Frenkel-excitonic locally singlet excited (LE) state and CT evolves as a function of time after photoexcitation. When external influences, such as solvent polarity, are strong, a pure, diabatic CT is the consequence. In low-polarity solvents, all NRs show significant triplet populations with energies that closely match the silicon bandgap. Furthermore, we show how the length of the NRs is a crucial factor in enabling intramolecular SF. For instance, in the shortest **NR-13**, triplet formation finds its origin in ISC, whereas in the longer **NR-33** and **NR-53**, triplets are generated via *i*-SF within a single NR along with some minor ISC contributions.

## Results and Discussion

We selected this set of NRs because they fulfill many of the requisites for SF materials, such as their (i) high stability, (ii) edge structure that generates multiple localized states, and (iii) triplet energies that are approximately half of those of the singlets. Previous TDDFT calculations^56^ showed that while in the case of **NR-13** only one excitation is possible, in the case of **NR-33** and **NR-53**, the generation of multiple excitons is in principle possible. In addition, to establish the triplet energies, we carried out TDDFT calculations with the B3LYP functional and the 6–31g­(d,p) basis set in chloroform with geometries optimized at the B3LYP/6–31g­(d,p) level. The triplet energies correspond in all cases to approximately half of those of the singlet energies (Table S12).

Another important feature is that all the NRs are perfectly soluble in organic solvents as they have been equipped with bulky tert-butyl, 1-hexylheptyl, and tri*iso*-butylsilyl-acetylene solubilizing groups. Also, the NRs present twists along the backbone that further contribute to the solubility, as non-planar systems are less prone to aggregate in solution. Such twists are generated by the steric congestion between the inner hydrogen atoms at the cove regions located at the pyrene-coronene junctions, which force both residues away from each other as seen by the single crystal X-ray structure of **NR-13** (Figure 1).^56^ Because of these multiple twists, the NRs can adopt different helical and alternated conformations that are in constant exchange at room temperature owing to the low interconversion barriers typical of these systems.^59,65,76,77^

**Figure 1:**
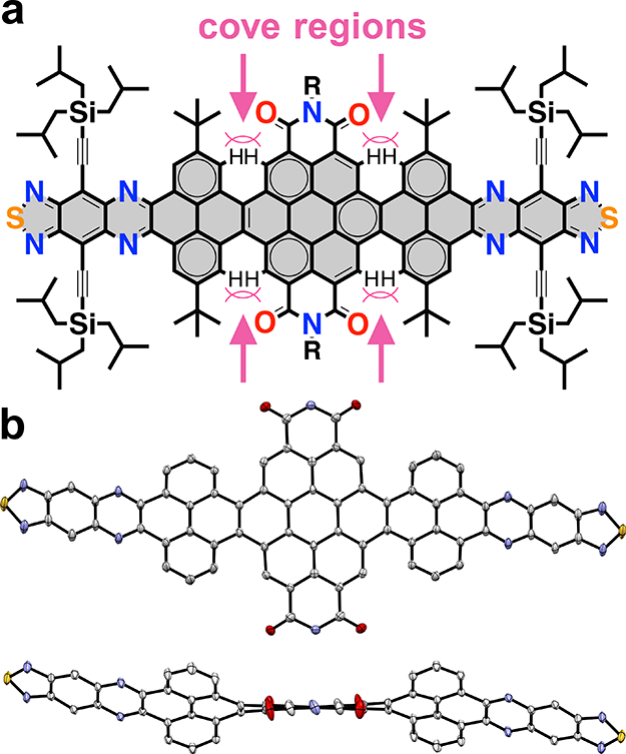
Twisted structure of the nanoribbons. (a) Chemical structure as well as (b) front and side views of the crystal structure of **NR-13**, with solubilizing groups removed for clarity, illustrating the steric crowding at the cove regions.

### Ground- and Excited-State Interactions—Admixture of Low-Lying Charge-Transfer to Locally (Singlet) Excited State

We probed the solvent-dependent ground- and excited-state interactions of NRs by absorption and emission experiments. The absorption of **NR-13** is characterized by three different bands, that is, the β-band around 405 nm, the α-band at around 470 nm, and the longest-wavelength ρ-band at roughly 600 nm (Figure S1a). The position and the peak molar extinction coefficient of the β-band (∼215,000 M^–1^ cm^–1^) are hardly influenced by the solvent polarity, suggesting their π–π* nature. The ρ-band absorption, however, is subject to a gradual broadening as well as attenuation upon increasing the polarity of the solvent, indicative for some CT character (Figure S1a; Tables S1 and S3). In general, the absorption is subject to sizable inhomogeneous broadening presumably due to the conformational flexibility along the longitudinal axis.^78,79^ The absorption of **NR-33** and **NR-53** are similar to that of **NR-13**. They also reveal β-band, α-band, and ρ-band absorptions at around 409, 470, and 600 nm (Figures S1a and S2). Overall, only minor bathochromic shifts stand in stark contrast to the substantial length increase (Table S3). This trend is consistent with that observed in other molecular NRs and nanographenes^56,63,64,71,73,80,81^ in which the absorption shifts rapidly saturate and remains almost invariable upon further length increase. When going from toluene to THF, the absorption features of **NR-33** and **NR-53** follow the trend that is summarized for **NR-13**. In particular, the ρ-band absorption considerably attenuates with respect to the β-band, and slightly broadens, albeit to a lesser extent than in **NR-13** (Table S3).

The conformational flexibility of the NRs prompted us to perform complementary temperature-dependent absorption experiments in 2-MeTHF. Looking at **NR-13** in 2-MeTHF, only a minor narrowing of the absorptions is discernible in the range from room temperature to 80 K (Figure S3a). **NR-33** and **NR-53** are different. Here, a narrowing of the ρ-band absorption goes hand-in-hand with a bathochromic shift of the longest-wavelength absorption and an evolution of a 580 nm shoulder (Figure S3b,c).

The emission spectra of all NRs are markedly influenced by the solvent polarity (Figure S1b). For **NR-13**, the 615 nm emission maximum is part of a somewhat pronounced vibronic fine structure in low-polarity toluene that spans from 600 to 750 nm. In THF, the gradual broadening of the emission leads to a loss of vibronic fine structure, further hinting at a significant CT character in the lowest-optical transition. Quite similar are the emission spectra of **NR-33** and **NR-53**. Here again, the emission is clearly structured in low-polarity toluene, and a gradual loss of the vibronic fine structure is noted in more polar THF. Lippert-Mataga plots, in which the bathochromic shifts of the emission and therewith associated Stokes shifts (Figures S4–S6) are plotted as a function of polarity, support a marked increase in the dipole moment of the excited states (Figure S7; Tables S1 and S2).^20,82,83^

All these observations point to a coupling between a diabatic LE and a CT that results in a mixed electronic state. An increase in solvent polarity stabilizes CT and lowers its energy, thereby narrowing the energy gap between LE and CT. This enables a stronger mixing of CT in the resulting mixed electronic state and causes the oscillator strength of the lowest-energy optical transition to be reduced. The CT character most likely stems from the stabilization in polar environments of the highly localized LUMOs at the terminal pyrazino-benzothiadiazoles, which is consistent with previous TDDFT calculations,^56^ and also from the distorted backbone that can further enhance the CT character of the lowest-energy optical transition.^84^ Notable is the fact that the CT admixture is strongest among the NRs for **NR-13**, regardless of the solvent. We attributed this to the shorter backbone of **NR-13** compared to **NR-33** and **NR-53** that favors a more localized polarization of the electronic density.

Considering again the conformational flexibility of this family of NRs, we perform temperature-dependent emission measurements. In 2-MeTHF, at room temperature, all the NRs feature an emission that is quite similar to that seen in THF (Figures 2a and S8), wherein a broad and structureless emission is complemented by a marked shoulder at around 660 nm. Upon lowering the temperature, a gradual red-shifting and a significant broadening is noted. At around 160 K, only an unstructured emission feature at 730 nm is left. Below 137 K, that is, in a frozen solvent glass matrix, the emission sharpens, and vibronic structure re-evolves. Fluorescence quantum yields parallel this trend as they drop from 17% at 297 K to a minimum of 8% at 160 K before they increase to 17% below 137 K (Table S4).^85^ Given the intrinsic flexibility in the NRs, we infer that CT formation is coupled to intramolecular vibrations and/or rotations. It is most likely some low-frequency twisting motions that are effectively suppressed in a frozen solvent glass matrix–vide infra. CT character in the emission turns out to be stronger in the low temperature range, a fact that we link to an increase of the effective polarity of the solvent.^86,87^ This increases the LE-CT coupling via stabilizing the CT, entailing a stronger CT contributions in the mixed state. The latter reduces radiative deactivation channels and promotes nonradiative ones. Independent evidence for this trend comes from the Lippert-Mataga plots, where larger Stokes shifts develop as the medium is gradually cooled down (Figure S9).

**Figure 2:**
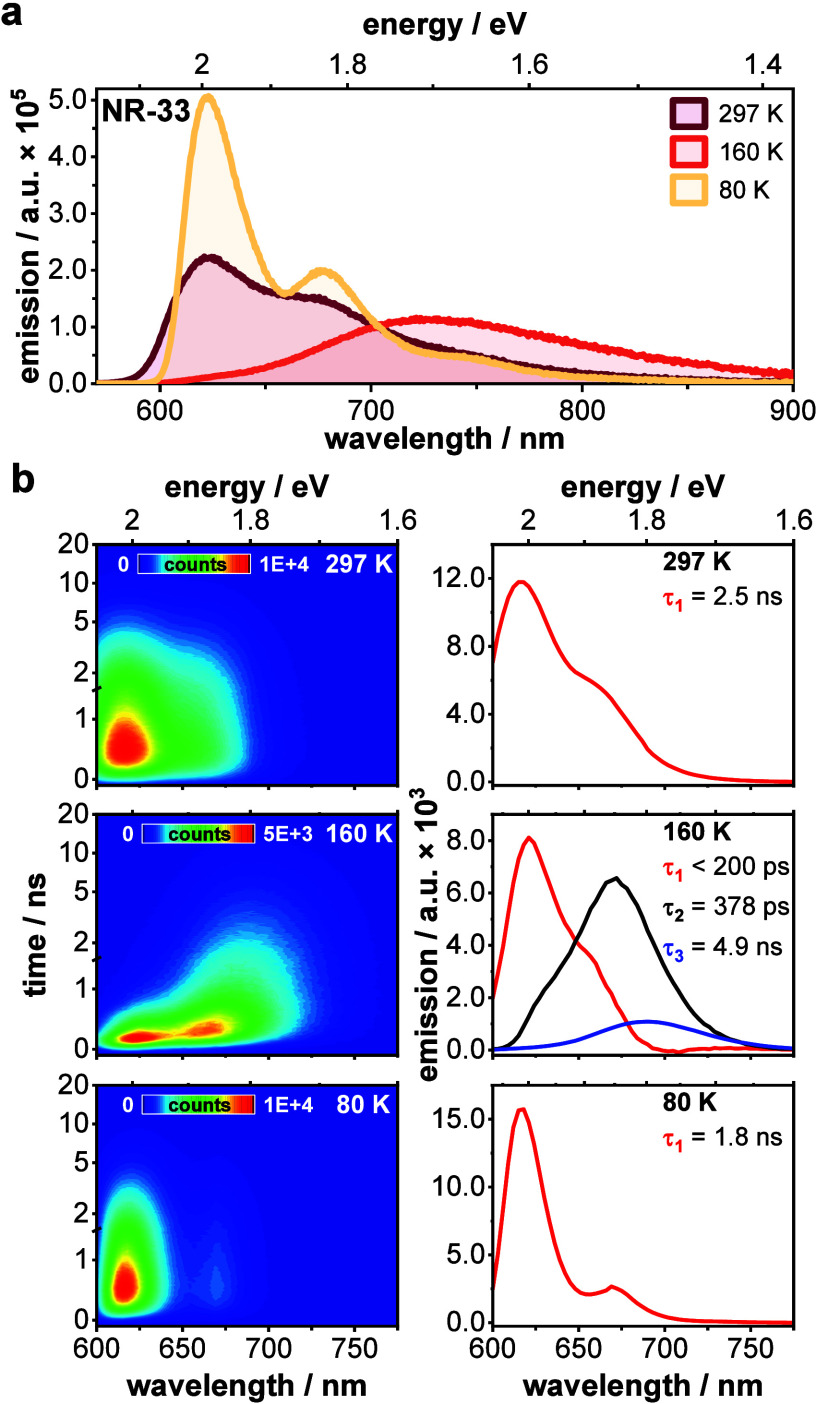
Temperature-dependent steady-state and time-resolved emission. (a) Steady-state emission spectra of **NR-33** recorded in 2-MeTHF after photoexcitation at 550 nm at selected temperatures (see Figure S8b for all temperatures). (b) Time-resolved emission 2D heat maps of **NR-33** along with their corresponding deconvoluted spectra obtained via global sequential analysis, recorded in 2-MeTHF after photoexcitation at 550 nm at 297 K (top), 160 K (middle), and 80 K (bottom). The optical densities were approximately 0.1 at the excitation wavelength for all samples.

Altogether, steady-state absorption and emission data support the presence of an intramolecular CT state. This is consistent with TDDFT calculations and the twisted nature of the NR, since a twisting, most likely, reduces orbital overlap, and in turn stabilizes a potential CT state.

### Excited State Dynamics of the Nanoribbons—Evolution of Charge-Transfer Character in the Mixed Electronic State

To deconvolute the polarity-dependent emission and, thereby, further elucidate the nature of the mixed electronic state, we turned to time-resolved emission spectroscopy (TRES). Interestingly, all decays upon 532 nm photoexcitation at room temperature were strictly monoexponential, irrespective of solvent polarity (Figure S10). The lifetimes decrease with increasing the length from **NR-13** to **NR-53** and lengthen with enhancing the polarity of the solvent. For instance, lifetimes of **NR-13** range from 4.4 ns in low-polarity toluene up to 7.8 ns in THF (Table S5). Considering that we only resolve a single emissive species, whose lifetime correlates with solvent polarity, corroborates the fact that the emission originates from a mixed electronic state, in which LE and CT are moderately coupled.^19,34,88^ Increasing CT contributions in the mixed electronic state evoke longer-lived emission. As the mixed electronic state picks up CT character, its geometry differs more strongly from that of the ground-state–vide infra. Admixing CT to LE also reduces the oscillator strength and, thus, further slows down the radiative recovery.

Given the aptitude of emission experiments to assess the CT character, we recorded time-resolved emission maps in 2-MeTHF and performed spectral deconvolution using GloTarAn to pinpoint the temporal evolution of the mixed state (Figures 2b, S11, and S12; Table S6).^89–91^ For **NR-33**, shown as a representative example, only a single emissive species with a 2.5 ns lifetime is resolved at room temperature. This is the emission that originates from the mixed electronic state. Upon cooling to 160 K, the emission of **NR-33** is deconvoluted into three different emissive species. Please note that the cooling and therewith associated increase in solvent polarity assists in a stronger coupling between diabatic LE and CT.^19,86^ For the short-lived emissive state (τ_1_ < 200 ps), clear vibronic structure is discernible. This finding suggests that the mixed electronic state exhibits a predominant LE character, whereas the CT character is minor. The intermediate- (τ_2_ = 378 ps) and long-lived (τ_3_ = 4.9 ns) emissions are reminiscent of structureless CT features, which are red-shifted by about 730 cm^–1^ relative to each other. From this, we deduce that the initially photogenerated electronic state collapses into a pure, diabatic CT, which subsequently undergoes solvent and structural relaxation. The changes in solvent polarity constitute the driving forces at lower temperatures.^19,92^ The observation of a red-shift, i.e., relaxation, occurring on a time scale of several hundreds of picoseconds in combination with the absence of pure, diabatic CT emission when cooling down beyond the freezing point of the solvent, at which twisting of the nanoribbon is restricted, provides further evidence for the critical role of intramolecular vibrational and torsional motion in the CT formation.^79^ As such, at 80 K, only a single emissive species is resolved. It appears with vibronic structure and decays with a 1.8 ns lifetime that is even shorter than what is seen at 297 K. As neither structural nor solvent reorganizations are feasible in the frozen solvent glass matrix, formation of a pure, diabatic CT as well as solvent and structural relaxation are ruled out. Instead, the ground-state geometry with a given LE to CT mixing is preserved in the excited state and radiative deactivation dominates the excited-state decay (Table S6).^92,93^ The dynamics of all relevant states will be explored in the next section.

To gain a full understanding of the temporal evolution of CT admixture in the mixed electronic state and the accompanying structural reorganization, we employed femtosecond transient absorption (fsTA) spectroscopy. Data sets were always analyzed by performing global analysis using GloTarAn.^89–91^ In Figure 3, room temperature fsTA data along with the corresponding evolution-associated spectra (EAS) in toluene and THF are contrasted. In toluene, the dynamics obtained from fsTA experiments are best described using a three-species sequential model. Following 550 nm photoexcitation, the immediate development of a very broad excited state absorption (ESA) that ranges from 420 nm all the way up to 1200 nm is observed. A closer inspection reveals maxima at around 480, 630, 695, 810, 905, and 990 nm. In addition, ground-state bleaching (GSB) is discernible at around 590 nm. These features are assigned to a hot mixed electronic state with predominant locally excited character (LE*) that undergoes solvent and structural reorganization with a lifetime of around 1 ps to yield LE, which then decays within nanoseconds (Table S8). Toward the end of the fsTA setup time window (ca. 5500 ps) a long-lived species remains. It exhibits ESA maxima in the visible region, that is, at around 476, 570, and 620 nm that partially overlap with the 590 nm GSB, while EASs in the near-infrared (NIR) region are absent. Our assignment is a triplet excited state (T_1_)–vide infra.^94,95^

**Figure 3:**
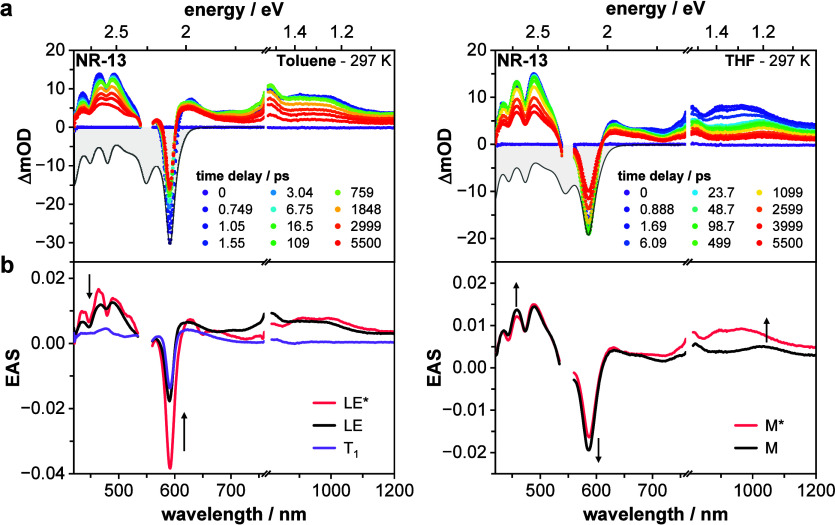
Early excited state dynamics of the nanoribbons. (a) Chirp- and zero-point-corrected differential transient absorption (TA) spectra of **NR-13** obtained from femtosecond TA experiments upon photoexcitation at 550 nm (500 nJ) in argon-saturated toluene and THF at room temperature with various time delays between 0 and 5500 ps (corresponding single-wavelength kinetics are shown in the Supporting Information in Figure S20; steady-state absorption in gray). (b) Corresponding deconvoluted evolution-associated spectra (EAS) of the initially populated mixed electronic state (red), which has predominantly locally excited character (LE*) or includes some charge-transfer contributions (M*), the subsequent solvent- and vibrationally relaxed mixed electronic states (LE and M, respectively; black), and the triplet excited state (T_1_; purple) as obtained by global analysis of the TA data shown in (a) using the kinetic models given in the Supporting Information in Figure S18.

Conversely, in solvents of higher polarity, such as THF, the deactivation cascade is modeled with a two-species sequential model. ESAs of the hot mixed electronic state—now with sizable CT contributions (M*)—are discernible immediately upon photoexcitation at 550 nm. From there on, solvent and vibrational relaxation sets in with a lifetime of 11 ps. Larger CT contributions in the mixed electronic state M* require larger solvent and structural adaptations, leading to relaxation occurring on longer time scales. In addition, ESAs and GSBs of M differ strongly from those of M*. Most notably, EASs at 905 and 990 nm are replaced by a new ESA at around 1050 nm (Figure S13). Features in the range of 420–540 nm are more intense, and the 590 nm GSB is more pronounced. Thus, we can take either the ESA around 450 nm or the GSB at 590 nm to assess the CT character in M. For example, an intensification reflects the time-dependent admixing of CT in the mixed electronic state due to structural and solvent fluctuations (Figures 4e and S66).^19,42^ Interestingly, the lifetime of M is considerably longer in THF than in toluene confirming our time-resolved emission experiments (Figure S20; Tables S5 and S8).

**Figure 4:**
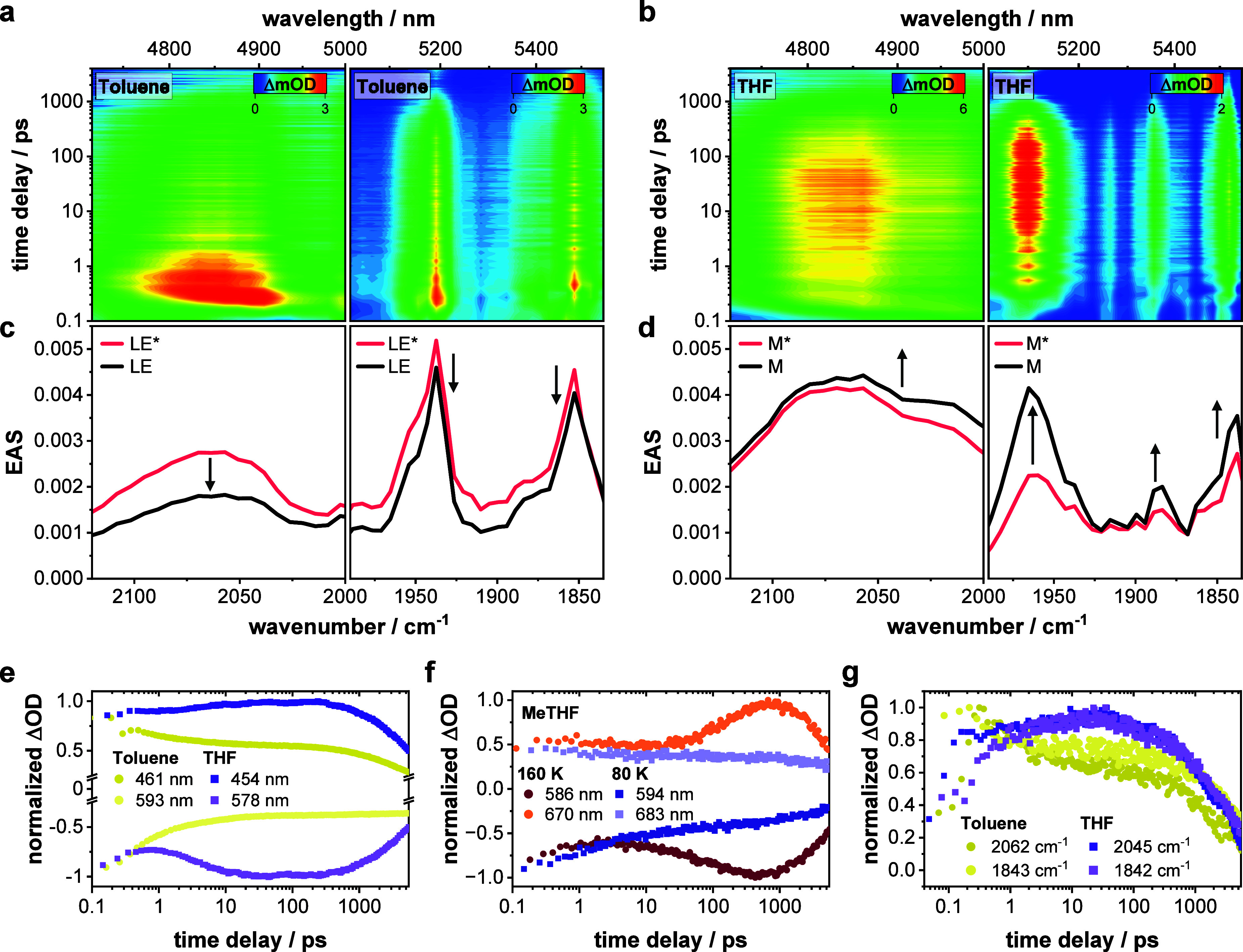
Evolution of charge-transfer character and therewith associated structural reorganization. Chirp- and zero-point-corrected differential transient infrared (IR) 2D heat maps of **NR-13** obtained from femtosecond transient IR absorption experiments upon photoexcitation at 550 nm (2.0 μJ) in (a) toluene and (b) THF at room temperature with various time delays between 0 and 5500 ps. Deconvoluted evolution-associated spectra (EAS) of the (c) predominantly locally excited state (LE*; red) and the subsequent solvent- and vibrationally relaxed locally excited state (LE; black) in toluene, as well as of the (d) mixed electronic state (M*; red) and the subsequent solvent- and vibrationally relaxed mixed electronic state (M; black) in THF as obtained by global analysis of the transient IR absorption data of **NR-13** shown in (a) and (b) using a two-species sequential kinetic model given in Figure S18. Single-wavelength dynamics from both transient electronic absorption (e, f) and IR (g) experiments highlighting the dependence of temporal CT admixture in the mixed state on external factors such as solvent polarity and temperature.

For **NR-33** and **NR-53**, the fsTA data in toluene were best fit with a three-species sequential model (Figures S14–S17, S19a, and S21–S24). Considering our conclusions for **NR-13**, the first and second species of **NR-33** and **NR-53** represent the mixed electronic state with predominant locally excited nature undergoing relaxation, that is, LE*→LE. Rather than assigning the long-lived species to a simple T_1_, it is assigned to a singlet correlated triplet pair state ^1^(T_1_T_1_) formed via *i*-SF. Nanosecond transient absorption (nsTA) spectroscopy further backs up this assignment–vide infra. Similar to **NR-13**, the lifetimes of LE/M depend on the solvent polarity for **NR-33** and **NR-53**, as a substantial increase is noted upon going from toluene to THF (Table S8). Simultaneously, the ^1^(T_1_T_1_) intensities drop to the extent that they are even unresolvable in THF. At this point, the use of a two-species rather than a three-species sequential model is imperative. At the forefront is the solvent and structural relaxation of M* to yield M (Figure S19b).^96^

Next, we carried out fsTA experiments at cryogenic temperatures. The fsTA data of **NR-13** recorded in 2-MeTHF at various representative temperatures, that is, 297, 160, and 80 K, are shown in Figures S25 and S32. At 297 K, the data obtained in 2-MeTHF match what we concluded for THF (Figures 3 and S25a). Immediately after photoexcitation, broad ESAs across the entire spectral range are notable. Maxima at around 490 and 620 nm in the visible region and from 800 to 1200 nm in the NIR region are the fingerprints of the mixed electronic state M*. The kinetic traces of the ESA at 490 nm and the GSB of the ρ-band substantiate that the CT character develops on a time scale of structural and solvent fluctuations (Figures S26 and S33).^19,42^

At 160 K, an additional third species becomes visible (Figures S25b and S31). Turning to the first species, it is virtually identical to the mixed electronic state M* that is seen in room temperature experiments. M* is short-lived and decays with a lifetime of 2.0 ps. Throughout this decay, a minor hypsochromic shift of GSB takes place and affords a 132 ps lived second species. Based on the good match to the lifetime derived from TRES experiments (Figure S11b; Tables S6 and S7), we assign the second species to a vibrationally hot charge-transfer state CT*. Following structural and solvent relaxation, a relaxed, presumably longitudinally ‘twisted’ CT is obtained–vide supra. The latter is characterized by 670, 820, and 1050 nm signatures as well as a 6.7 ns lifetime. Spectro-electrochemistry experiments support the CT nature of such state. Specifically, a similar set of peaks is observed upon reduction of the nanoribbons at applied potentials lower than −0.8 V (Figure S66a).^56^

At 80 K, despite the further solvent polarity increase, no gradual CT admixture to LE in the mixed state occurs, as the necessary solvent and structural reorganizations are suppressed by the frozen solvent glass matrix (Figures 4f and S25c).^86^ This affects the kinetic modeling, where a two-species rather than a three-species model now suffices. Following relaxation, M uniformly deactivates back to the electronic ground state with a lifetime that is with 5.7 ns shorter at 80 K than at 297 K with 6.6 ns. These observations are in line with the results obtained from TRES measurements and, thus, further support that intramolecular torsional motions are key drivers of CT formation in the NRs.

Quite similar are the temperature-dependent dynamics for **NR-33** and **NR-53** on the femtosecond time scale (Figures S27–S30 and S34–S37). Only the ESAs of CT are narrower and blue-shifted with respect to **NR-13** from, for example, 670 to 630 nm or 1050 to 910 nm (Figures S25, S27, and S29). Such observation is reasonable considering that the shorter backbone of **NR-13** favors a more localized polarization of the hole density. For **NR-33** and **NR-53**, the hole density is more delocalized across the longer backbone, resulting in weaker CT character.

Complementary transient infrared experiments were performed for **NR-13** at room temperature to follow the evolution of the CT character in the mixed electronic state and the associated reorganization as a function of solvent polarity (Figures 4, S38, and S39). In toluene as well as THF, a continuous broad absorption and several ESAs were instantly observed across the ∼2120 to 1835 cm^–1^ range. These are ascribed to the hot mixed electronic state.^97^ The data is best fit with a two-species sequential model, yielding one lifetime of a few picoseconds and another one of several nanoseconds. These are consistent with the lifetimes obtained in the TRES and electronic fsTA experiments (Tables S5 and S8). Our observation that similar vibrations are active in both solvents supports the notion that the lowest-energy optical transition features some CT character.

This is consistent with previous TDDFT calculations that show how the HOMO–LUMO transition, characterized by a strong CT nature, exhibits significant oscillator strength.^56^ In low-polarity toluene, the initial CT lacks, however, a significant stabilization and causes the mixed state to rapidly evolve to a LE. Evidence came from an abrupt decrease in signal intensity right after the photoexcitation (Figure 4a,c). In contrast, the temporal ESA evolution in THF is quite different (Figure 4b,d). Maximum EAS intensities are not reached before roughly 30 ps have passed, a finding that is in sound agreement with the trends observed in electronic fsTA experiments (Figure 4e,g). Such an ESA increase is linked to changes in the wave function overlaps due to geometric modifications. A similar behavior is observed in an excimer/mixed state if relaxation is accompanied by nuclear reorganization.^98,99^ In other words, after the lowest-energy transition in polar solvents, the system adapts to changes in electron density through structural reorganization. Possible reorganization modes are the backbone twists at the NR termini. This, in turn, can interfere with orbital overlap. As the EASs persist despite the increased CT character even on longer time scales, we infer that the mixed state does not fully transition into a pure, diabatic CT at room temperature. Independent confirmation comes from temperature-dependent measurements. These document that a pure CT is only achieved at lower temperatures, at a sufficient driving force, and at low thermal energy (Figures S25b, S32b, and S33b).^100^

### Excited State Dynamics of the Nanoribbons—Singlet Fission in the Extended Ribbons

The long-lived species seen in the fsTA experiments necessitated nanosecond transient absorption (nsTA) experiments.

The dynamics of **NR-13** in both toluene and THF are modeled well with a two-species sequential model (Figure S46). Immediately after photoexcitation, features of LE/M are observable (Figures 5, S40, and S41). LE/M decays within nanoseconds, which affords a long-lived species, which is characterized by ESA maxima at 480, 570, and 620 nm. As these features not only match the EAS of T_1_ generated in the triplet-sensitization experiments but also its lifetimes (τ_T1_ ∼ 3 μs), it is assigned to T_1_ (Figure S55). The slow formation is indicative for a spin-forbidden ISC. Triplet quantum yields in toluene are remarkably high reaching 79%.^101,102^ Such high yields correlate with, first, an efficient ISC due to the significant CT character, second, the backbone’s conformational flexibility, and, third, the presence of sulfur in the electron-accepting terminal units.^43,95,103^ Increasing the solvent polarity from toluene to THF leads to an increase of the LE/M lifetime (Table S8) and to a decrease of triplet quantum yields.

**Figure 5:**
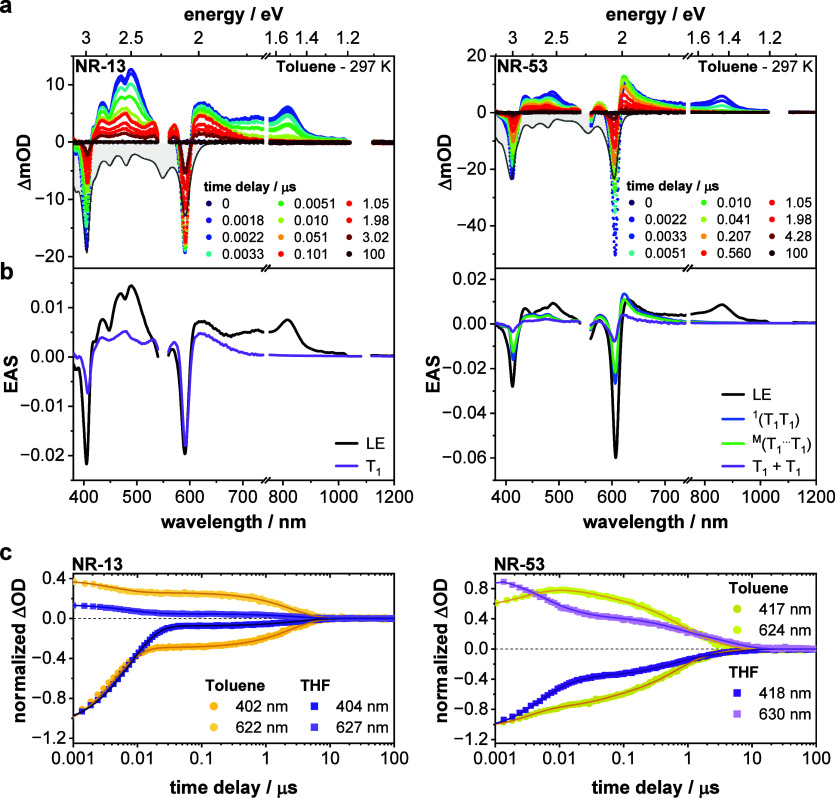
Triplet excited state dynamics of the nanoribbons. (a) Zero-point-corrected differential transient absorption (TA) spectra of **NR-13** (left) and **NR-53** (right) obtained from nsTA experiments upon photoexcitation at 550 nm (400 nJ) in argon-saturated toluene at room temperature with various time delays between 0 and 100 μs (corresponding single-wavelength kinetics are shown in the Supporting Information in Figures S49 and S54; steady-state absorption in gray). (b) Corresponding evolution-associated spectra (EAS) of the predominantly locally excited state (LE; black), the singlet correlated triplet pair state (^1^(T_1_T_1_); blue), the subsequent weakly coupled triplet pair state (^M^(T_1_···T_1_); green), and the free triplet excited state (T_1_ and (T_1_ + T_1_), respectively; purple) as obtained by global analysis of the TA data shown in (a) using the kinetic models given in the SI in Figures S46 and S47. (c) Single-wavelength kinetics and corresponding fits of **NR-13** and **NR-53** obtained from nsTA experiments contrasting the multiexponential decay of **NR-53** with the biphasic decay of **NR-13** and highlighting the reduced formation of long-lived species (namely, triplet excited states) in higher-polarity solvents, regardless of the formation pathway.

Peculiarly, the nsTA data of **NR-33** and **NR-53** necessitate a four-species sequential model to fit the experimental results (Figures S42–S45, S47, and S50–S54). As observed for **NR-13**, photoexcitation at 550 nm initially populates the LE/M state. Concurrently with the decay of LE/M, new spectral features emerge that resemble those of T_1_. However, in stark contrast to **NR-13**, the overall triplet dynamics in **NR-33** and **NR-53** reveal, on one hand, a notably faster triplet formation and, on the other hand, a three-exponential decay (Figure 5c). Deconvolution of the triplet decay reveals three distinct triplet species with markedly different lifetimes. The first triplet species exhibits a lifetime in the range of several tens of nanoseconds (16 ns for **NR-33**; 32 ns for **NR-53**), whereas the lifetime of the second triplet species is in the range of several hundreds of nanoseconds (245 ns for **NR-33**; 560 ns for **NR-53**). The lifetime of the third triplet species is on the order of microseconds (4.5 μs for **NR-33** and **NR-53**). While the lifetime of the third triplet species matches the lifetime of the ISC-born T_1_ seen for **NR-13**, the lifetimes of the first two triplet species are considerably shorter and more in line with lifetimes observed for intermediate triplet pairs seen in weakly coupled SF systems. Following this, the first and second triplet species are assigned to a singlet correlated triplet pair state ^1^(T_1_T_1_) and a weakly coupled triplet pair ^M^(T_1_···T_1_), respectively, which are formed via *i*-SF within a single NR.^15,17,20–22,104,105^ This leaves the third triplet species to be the free triplet excited state (T_1_ + T_1_) following decoherence (Figures 6, S56, and S57). The occurrence of *i*-SF within individual extended NRs is rationalized by their push–pull nature and CT character in the excited state. Such substantial CT character is the result of a redistribution of electron density between the coronene-centered HOMOs to the electron-accepting pyrazino-centered LUMOs, which is facilitated by the twisted structure.^56,84^ This CT nature, combined with the rapid interconversion between conformers, facilitates the necessary interactions by enabling superexchange-mediated coupling to the multiexcitonic state.^38,82^ However, while the bridging units presumably favor *i*-SF, the terminal units—due to the presence of sulfur—are more likely to promote ISC. This explains why only the extended nanoribbons exhibit *i*-SF, whereas very efficient ISC is seen for **NR-13**.^106^ An additional driving force for *i*-SF arises from the delocalization of the initial LE/M state. Rather than being confined to the terminal units, it is distributed across the bridging quinoxalines for **NR-33** and **NR-53**. Upon *i*-SF, the correlated triplet pair state is preferentially localized onto the electron-accepting bridging pyrazino-quinoxalines or terminal pyrazino-benzothiadiazoles, likely due to their favorable T_1_ energies.^81,107^ More generally, any delocalization of the initial state renders the formation of localized correlated triplet pairs entropically favorable.^79,108–111^ The observed decrease in LE/M lifetimes with increasing nanoribbon length, from **NR-33** to **NR-53**, supports the notion that the initial mixed electronic state becomes increasingly delocalized, thereby enhancing exchange couplings to the multiexcitonic state.^79,112^ In line with **NR-13**, the overall triplet yields of **NR-33** and **NR-53** decrease with increasing solvent polarity, whereas the lifetime of LE/M increases with polarity. In general, all lifetimes derived from global analysis of the nsTA data match the lifetimes of LE/M obtained in the TRES and fsTA experiments (Tables S5 and S8).

**Figure 6:**
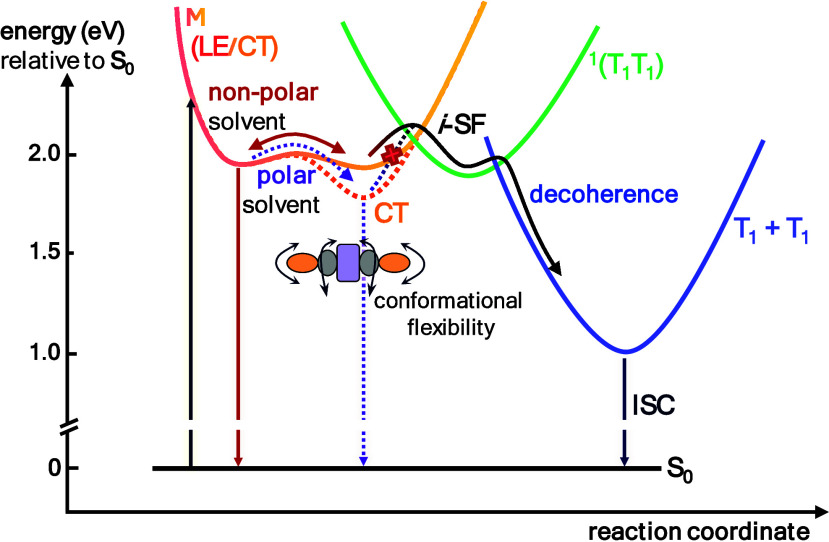
Deactivation cascade of the extended nanoribbons. Proposed qualitative deactivation scheme for **NR-33** and **NR-53**. Following the lowest-energy optical transition, the system transitions into a state exhibiting an admixture of locally excited (LE) and charge-transfer (CT) character owing to its electron donor–acceptor nature. The environment governs the ratio of LE to CT and, thereby, influences the subsequent deactivation cascade. In nonpolar solvents, the CT character is only weakly pronounced, which favors intramolecular singlet fission (*i*-SF). However, in polar solvents, stabilization of the CT state shifts the dynamics toward the formation of a diabatic CT state, whose formation is accompanied by significant structural reorganization. The deactivation cascade of **NR-13** is shown in the SI in Figure S65.

The yields of ^1^(T_1_T_1_), ^M^(T_1_···T_1_), and T_1_ + T_1_ were further investigated for **NR-33** and **NR-53** in toluene only. ^1^(T_1_T_1_) yields are obtained by extrapolating the singlet oxygen quantum yields (see SI, Chapter 2.3.1.2 ‘Triplet quantum yields’ for details). Based on these, the yields of ^1^(T_1_T_1_) are estimated to reach 122 and 111% in toluene for **NR-33** and **NR-53**, respectively (Figures S58 and S59; Tables S9 and S10).^113^ Determination of the triplet dissociation yields is carried out by comparing the intensities of the EASs for the different triplet species. Triplet dissociation yields from ^1^(T_1_T_1_) to ^M^(T_1_···T_1_) are very high in toluene at room temperature, reaching values of up to 87% for **NR-33** and 81% for **NR-53**, respectively. Further dissociation from ^M^(T_1_···T_1_) to T_1_ + T_1_ reaches values of 43% for **NR-33** and 35% for **NR-53**. We rationalize the high triplet dissociation yields as follows. On one hand, **NR-33** and **NR-53** represent weakly coupled systems and, in turn, the intertriplet exchange coupling *J* is small. As a result, structural fluctuations may drive spin evolution via mixing between ^1^(T_1_T_1_) and other triplet manifolds to yield ^5^(T_1_T_1_), which gateways the efficient dissociation of the triplet pair into free triplets in SF.^14,16,114^ As free rotations are excluded in the nanoribbons, changes in orientation are somewhat restricted for **NR-33** and **NR-53**. This is beneficial as it allows mixing of ^1^(T_1_T_1_) to ^5^(T_1_T_1_), but suppresses mixing to ^3^(T_1_T_1_).^7,16,22,115,116^

To unravel how temperature influences SF, in general, and to elucidate the role of torsional motion/disorder on the triplet dissociation, in particular, additional nsTA experiments were performed at variable temperatures down to 200 K in toluene for **NR-33** (Figures S60–S62). Data were consistently analyzed with the same four-species kinetic model used for the room temperature experiments of **NR-33** given in Figure S47. Global analysis shows that ^1^(T_1_T_1_) yields remain largely consistent across the temperatures; 119% at 297 K and 112% at 200 K.^117^ Lowering the temperature impacts, however, the formation rates of ^1^(T_1_T_1_). Plotting k_SF_ as a function of temperature in an Arrhenius plot yields an activation energy of roughly 120 cm^–1^ (Figure S63). We link such a low energy barrier to a low-frequency torsional mode. An interconversion between various twisted conformers is key in temporally increasing the planarization and, thus, maximizing the electronic coupling. Lower temperatures impair such interconversions as torsional motions are reduced. The immediate impact is not only observed in the formation dynamics and yields of ^1^(T_1_T_1_), but also the subsequent decoherence thereof. For example, ^1^(T_1_T_1_) lifetimes rise from 15 ns at 297 K to 176 ns at 200 K, whereas ^1^(T_1_T_1_) to ^M^(T_1_···T_1_) dissociation yields decrease from 85% at 297 K to 36% at 200 K (Figure S64 and Table S11). Such observations can also be attributed to reduced geometry fluctuations at lower temperatures, which preclude effective conversion of ^1^(T_1_T_1_) to ^5^(T_1_T_1_). Intramolecular motions either vibrational or torsional are required to modulate *J* and to overcome the energy gap between ^1^(T_1_T_1_) and ^5^(T_1_T_1_).^14,118^ Without it, ^M^(T_1_···T_1_) yields will be low, entailing increased ^1^(T_1_T_1_) lifetimes. ^1^(T_1_T_1_) lifetimes may be further increased as nonradiative internal conversions are slowed down toward the low temperature regime.^119^ Overall, the great temperature sensitivity of the triplet dynamics in the extended **NR-33** underscores the strong dependence of *i*-SF on molecular geometry. It also demonstrates the potential of weakly to moderately coupled and relatively rigid SF systems as materials for achieving high yields of ^5^(T_1_T_1_) and ^M^(T_1_···T_1_), respectively.

## Conclusions

Overall, we have shown how individual semiconducting molecular N-doped graphene NRs, owing to the edge-induced multiple localized π-electron states, can function as coupled multichromophoric systems, thereby enabling the generation of triplets through intramolecular singlet fission at the single NR level. Furthermore, we show how the length of the NRs is a crucial factor in enabling *i*-SF. **NR-13** generates triplets exclusively via intersystem crossing, which is attributed to the stronger push–pull character between the pyrazino-benzothiadiazole termini and the shorter backbone. In contrast, the attenuated push–pull character and suitable triplet energies of the bridging pyrazino-quinoxaline acceptors (of roughly half the energy of the mixed state) in **NR-33** and **NR-53** enable *i*-SF as additional deactivation pathway within an individual NR rather than requiring dimeric interactions. Low intertriplet exchange interactions coupled with moderate torsional flexibility allow for efficient triplet decoherence of the intermediate triplet pair state ^1^(T_1_T_1_). Higher-polarity solvents, however, promote an evolution toward higher CT admixture in the mixed state or even a pure (diabatic) CT state for all NRs, entailing significant geometric relaxation through twisting along the longitudinal axis. This compromises triplet generation as the energy of the CT is lowered to such an extent that the CT state acts more as a trap state rather than as a mediator for intramolecular SF. These findings offer insights that will enhance the manipulation of excited-state dynamics, open new application potential for N-doped graphene NRs, and provide new approaches to designing molecular NRs for intramolecular SF, charge separation, and potentially even thermally activated delayed fluorescence.

## Methods

### Synthesis

The detailed synthetic procedure and purity characterization of the materials are provided in ref 56.

### Steady-State Absorption Spectroscopy

Steady-state absorption experiments at room temperature were performed using a PerkinElmer Lambda 2 UV/vis double beam spectrometer (190–1100 nm). Temperature-dependent steady-state absorption experiments at temperatures down to 80 K were recorded using an optical cryostat optistatDN-2 from Oxford Instruments with a MercuryiTC temperature controller coupled to a Cary 5000 UV–vis-NIR spectrometer. All spectra were acquired using standard 10 × 10 mm quartz glass cuvettes.

### Steady-State Emission Spectroscopy

Steady-state emission spectra at room temperature were acquired on a Fluoromax-3 Fluorometer from Horiba Jobin Yvon. Fluorescence quantum yields were determined by means of gradient analysis (with optical densities of the samples ranging from 0 to 0.025), as described by Williams et al.,^120^ using Cresyl Violet in ethanol (Φ_F_ = 0.58) as reference.^121^ Temperature-dependent steady-state emission experiments at temperatures down to 80 K were conducted using an optistatDN-2 coupled to a FS5 spectrofluorometer from Edinburgh instruments. Singlet oxygen emission spectra were recorded using a FluoroLog3 emission spectrometer from Horiba Jobin Yvon coupled to a Symphony II detector in the NIR detection range. The samples were purged with oxygen gas for 20 min.

### Steady-State Infrared Spectroscopy

ATR infrared spectra were recorded using a FT-IR Prestige 21 spectrometer from Shimadzu.

### Time-Resolved Emission Spectroscopy

Fluorescence decay curves at room temperature were recorded using FS5 spectrofluorometer from Edinburgh instruments in a time-correlated single photon counting configuration. A pulsed UV–vis picosecond laser system from PicoQuant was used to generate the excitation pulses. Time-resolved emission maps at different temperatures were recorded using an optistatDN-2 coupled to a FluoroLog-3 spectrofluorometer from Horiba Jobin Yvon with a Hamamatsu MCP photomultiplier (R3809U-58). For excitation, a laser diode (NKT Photonic) was used. All samples were deoxygenated for approximately 15 min using argon gas and measured at an optical density of roughly 0.1 at the respective excitation wavelength.

### Transient Absorption Spectroscopy

Femtosecond transient absorption (fsTA) experiments were conducted employing an amplified Ti:sapphire CPA-2101 laser system from Clark:MXR Inc. as excitation source. The generated lasers pulses are characterized by an initial wavelength of 775 nm, a pulse width of 150 fs, and a frequency of 1050 Hz. The detection of the data was realized using a HELIOS “transient absorption pump-probe system” (TAPPS) detection unit from Ultrafast Systems. The 550 nm excitation wavelength, with energies of 50–400 nJ, was generated by a noncolinear optical parametric amplifier (NOPA, Clark:MXR Inc.), whereas the white light (probe pulse) is generated by a sapphire crystal. An optical delay line from Ultrafast Systems allowed for time delays up to 5500 ps. The experiments were carried out using fused quartz glass cuvettes with a width of 2 mm. Nanosecond transient absorption (nsTA) experiments were recorded with the EOS TAPPS detection unit from Ultrafast systems using two independent pulsed laser sources. While the pump pulse is also generated by an amplified Ti:sapphire CPA-2101 laser system from Clark:MXR Inc., the probe pulse is generated by a pulsed supercontinuum laser (output 350–2,200 nm, repetition rate of 2100 Hz, and 1 ns pulse width). The electronic setup allows for time delays up to 400 μs between the pump and probe pulse. The excitation wavelength of 550 nm was generated by a noncolinear optical para-metric amplifier (NOPA, Clark:MXR Inc.). Temperature-dependent transient absorption experiments were realized by coupling an optistatDN-2 (Oxford Instruments) to the same TAPPS system as described above. For all fsTA and nsTA experiments, samples were deoxygenated for approximately 15 min using argon gas and measured at an optical density of roughly 0.2 at the excitation wavelength.

Transient IR (fsIR) experiments were performed employing an Astrella-F-1K amplified Ti:sapphire femtosecond laser system from Coherent, operating at a repetition rate of 1 kHz with a pulse duration of 80 fs, reaching a power of 5.5 W (5 mJ pulse energy), and exhibiting an initial wavelength of 800 nm. To acquire the fsIR spectra on a subps and ns resolution, a commercial HELIOS IR transient absorption spectrometer was used in combination with a delay line providing a temporal time window of 8 ns (both from Ultrafast Systems). The probe pulse in the mid-infrared region (∼2.6 to 11 μm) was generated via a TOPAS Prime from Light Conversion with a standard nDFG extension kit. Inside the fsIR spectrometer, the IR probe pulse was divided into a reference and probe beam, which are detected by nitrogen cooled 32 × 2 pixel MCT detector. The sample (concentration of around 0.1 mM) was prepared in a home-built IR cell using two CaF2 windows and a Teflon spacer of 250 μm.

### Data Analysis

For the fsTA and nsTA measurements, global analysis of the transient data was carried out utilizing the open-source software GloTarAn, which is a free, Java-based graphical user interface to the R-package TIMP.^89–91^ Global analysis was performed on the TA data sets using a sequential model. The instrument response function and dispersion (chirp of the white light pulse) were taken into account and modeled during the fitting procedure. The nsTA data sets were corrected for scattered light as well.

### Spectro-Electrochemistry

Spectro-electrochemistry measurements were performed in argon-purged solutions of dichloromethane employing an AutoLab PGStat302N potentiostat (with a Pt-gauze as working electrode, Pt-wire as counter electrode and Ag-wire as pseudoreference electrode) and tetrabutylammonium hexafluorophosphate TBA­(PF_6_), 0.1 M as supporting electrolyte. The spectra were collected by using an AvaLight-DH-S-BAL Balanced Power lamp (215–500 nm deuterium, 500–2500 nm Halogen) coupled to an AvaSpec-ULS2048 StarLine spectrometer.

## Supplementary Material



## Notes

Additional steady-state absorption and emission data, time-resolved emission data, and transient absorption data on the femtosecond and nanosecond time scales (along with the corresponding kinetic models used to fit the data) for **NR-13**, **NR-33**, and **NR-53** at both room and cryogenic temperatures; and spectro-electrochemistry data for **NR-13** and computational details (PDF)
