# Supplementary material for: Intramolecular Singlet Fission in Individual Graphene Nanoribbons—Competition with a Charge Transfer
Source: J Am Chem Soc. 2025 Mar 19;147(13):11277–90. doi: 10.1021/jacs.4c18051 (PMC12272549; doi:10.1021/jacs.4c18051)
Supplement: Supplementary file 1 [file ja4c18051_si_001.pdf]

Supporting Information for

Intramolecular Singlet Fission in Individual Graphene Nanoribbons—Competition with a Charge Transfer

Phillip M. Greißel,[a] Giovanni M. Beneventi,[a] René Weiß,[a] Anna-Sophie Wollny,[a] Rajeev K. Dubey,[b] Manuel Melle-Franco,[c] Timothy Clark,[d] Aurelio Mateo-Alonso*,[b,e] and Dirk M. Guldi*[a]

[a] Department of Chemistry and Pharmacy & Interdisciplinary Center for Molecular Materials (ICMM), Friedrich-Alexander-University Erlangen-Nuremberg, Egerlandstraße 3, 91058 Erlangen, Germany.

[b] POLYMAT, University of the Basque Country, Avenida de Tolosa 72, 20018 Donostia-San Sebastian, Spain.

[c] CICECO - Aveiro Institute of Materials, Department of Chemistry, University of Aveiro, 3810-193 Aveiro, Portugal

[d] Department of Chemistry and Pharmacy & Computer-Chemie-Center (CCC), Friedrich-Alexander-University Erlangen-Nuremberg, Nägelsbachstraße 25, 91052 Erlangen, Germany.

[e] Ikerbasque, Basque Foundation for Science, 48009 Bilbao, Spain

Correspondence to: amateo@polymat.eu (AMA), dirk.guldi@fau.de (DMG)

Table of Contents

1. Steady-state absorption and emission data 3
1.1 Absorption data 3
1.2 Emission data 6
2. Time-resolved spectroscopy 15
2.1 Time-resolved emission 15
2.2 Femtosecond transient absorption spectroscopy data 20
2.2.1 Femtosecond transient absorption spectroscopy experiments at room temperature 20
2.2.2 Femtosecond transient absorption spectroscopy experiments at cryogenic temperatures 32
2.2.3 Femtosecond transient infrared spectroscopy 46
2.3 Nanosecond transient absorption spectroscopy data 48
2.3.1 Nanosecond transient absorption spectroscopy experiments at room temperature 48
2.3.1.1 Triplet-sensitization experiments 63
2.3.1.2 Triplet quantum yields 65
2.3.2 Nanosecond transient absorption spectroscopy experiments at cryogenic temperatures 68
3. Spectro-electrochemistry 75
4. Computational analysis 76
5. References 78

1. Steady-state absorption and emission data

1.1 Absorption data

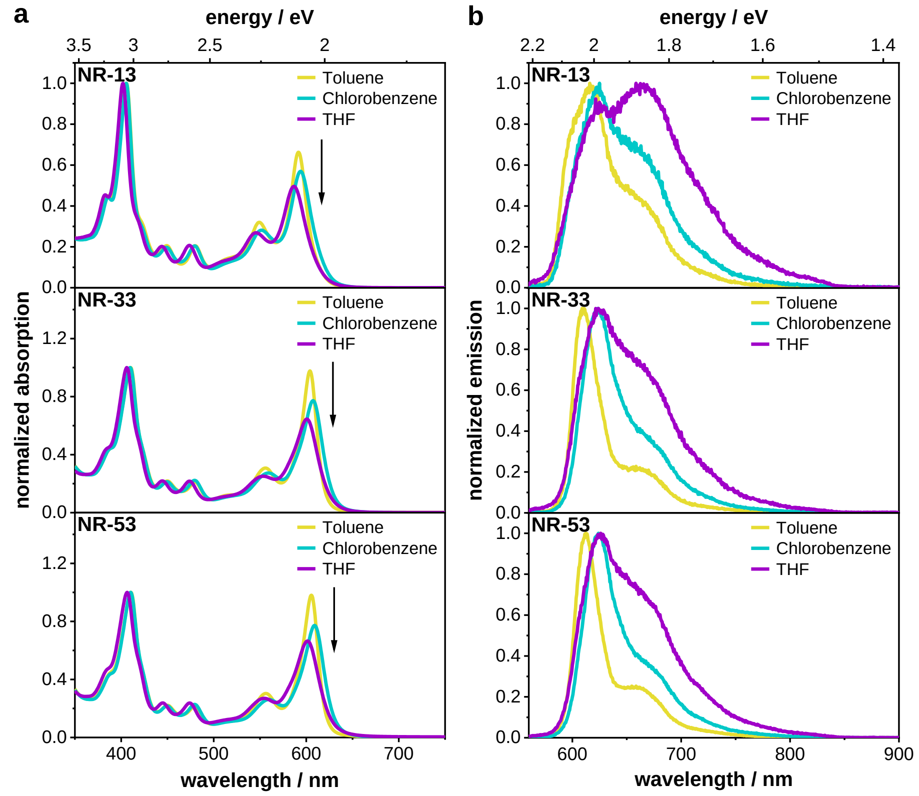

Figure S1 | Steady-state absorption and emission spectra at room temperature. (a) Normalized steady-state absorption spectra of the nanoribbons (NRs) **NR-13**, **NR-33**, and **NR-53** recorded in solvents of varying polarity (see figure legend) at room temperature. The spectra are normalized with respect to their respective maximum of the β-band, that is, around 410 nm. (b) Normalized steady-state emission spectra of the NRs recorded in solvents of varying polarity (see figure legend) at room temperature.

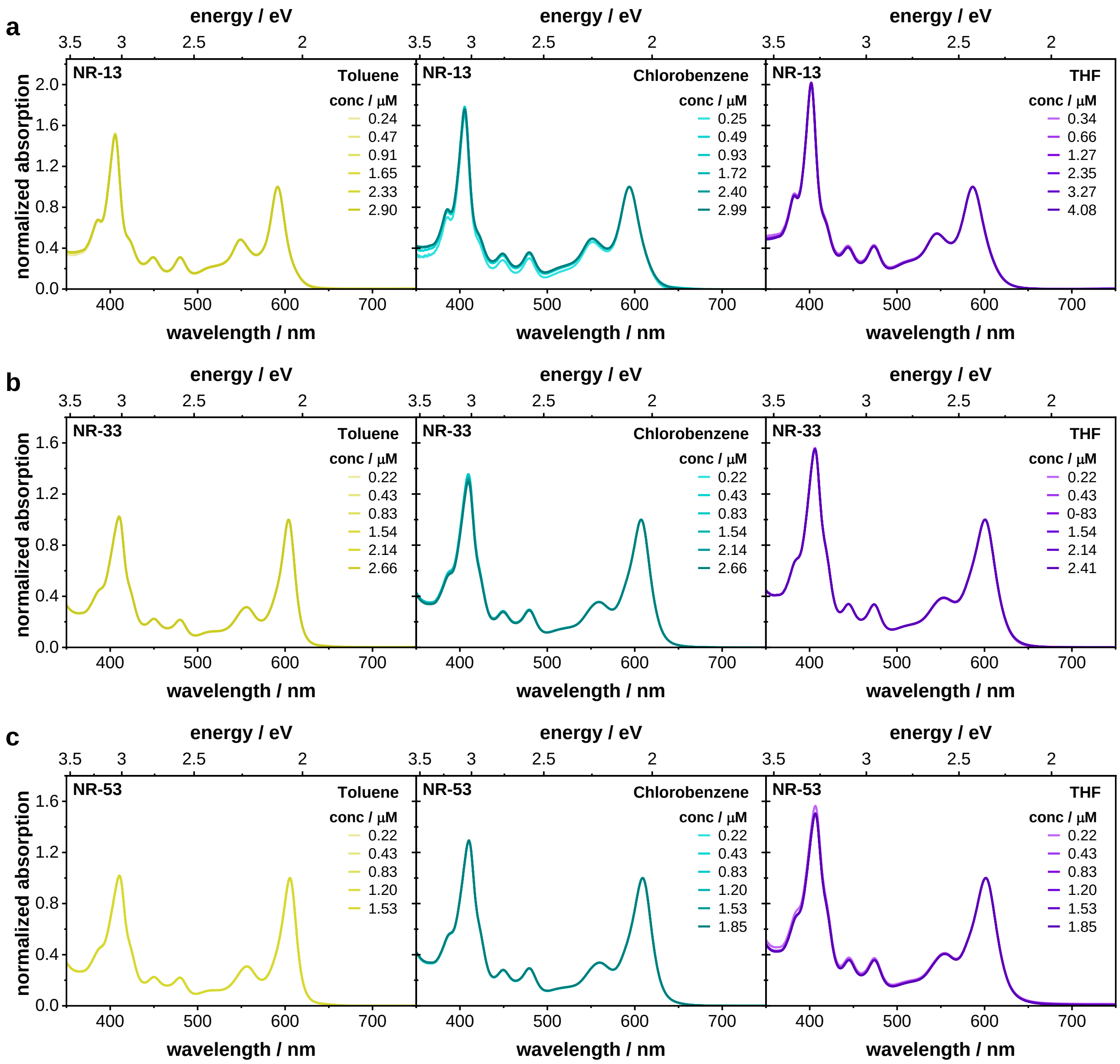

Figure S2 | Concentration-dependent steady-state absorption spectra at room temperature. Normalized steady-state absorption spectra of (a) **NR-13**, (b) **NR-33**, and (c) **NR-53** recorded in solvents of varying polarity at different concentrations up to a few µM. The spectra are normalized with respect to their longest-wavelength absorption maximum, that is, around 600 nm. No deviations are observed with increasing concentration, indicating no aggregation within the concentration range used for further photophysical characterization.

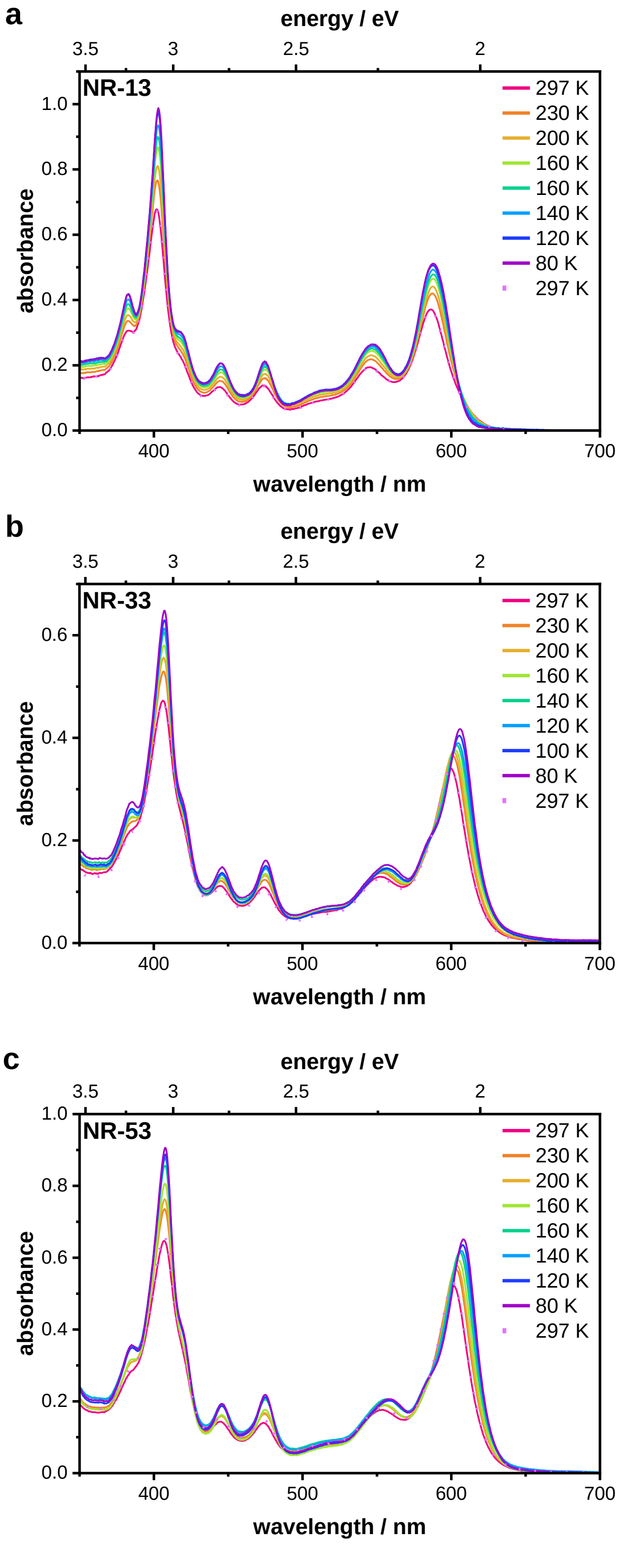

Figure S3 | Temperature-dependent steady-state absorption. Temperature-dependent steady-state absorption spectra of (a) **NR-13**, (b) **NR-33**, and (c) **NR-53** recorded in 2-MeTHF at various temperatures (see figure legend for exact values).

1.2 Emission data

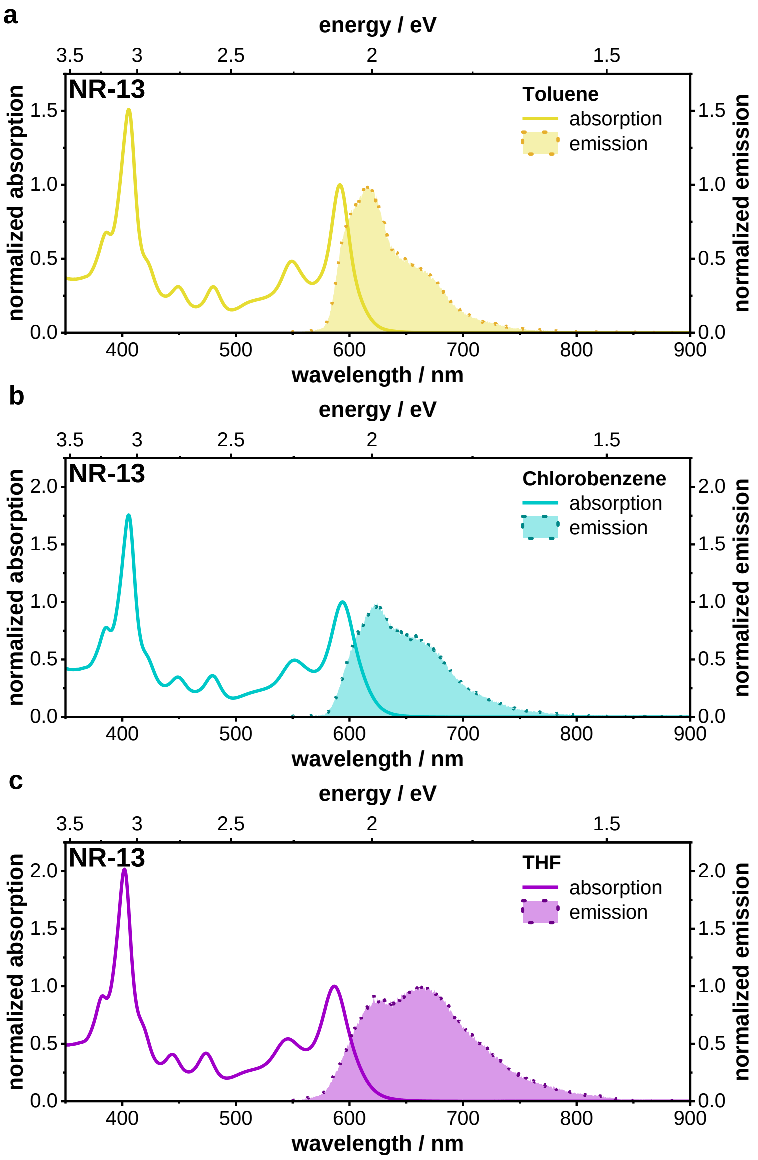

Figure S4 | Stokes shift plots. Normalized steady-state absorption spectra plotted along with normalized steady-state emission spectra after photoexcitation at 550 nm of **NR-13** in (a) toluene, (b) chlorobenzene, and (c) THF highlighting the Stokes shift. Absorption spectra were normalized with respect to their respective longest-wavelength absorption maximum.

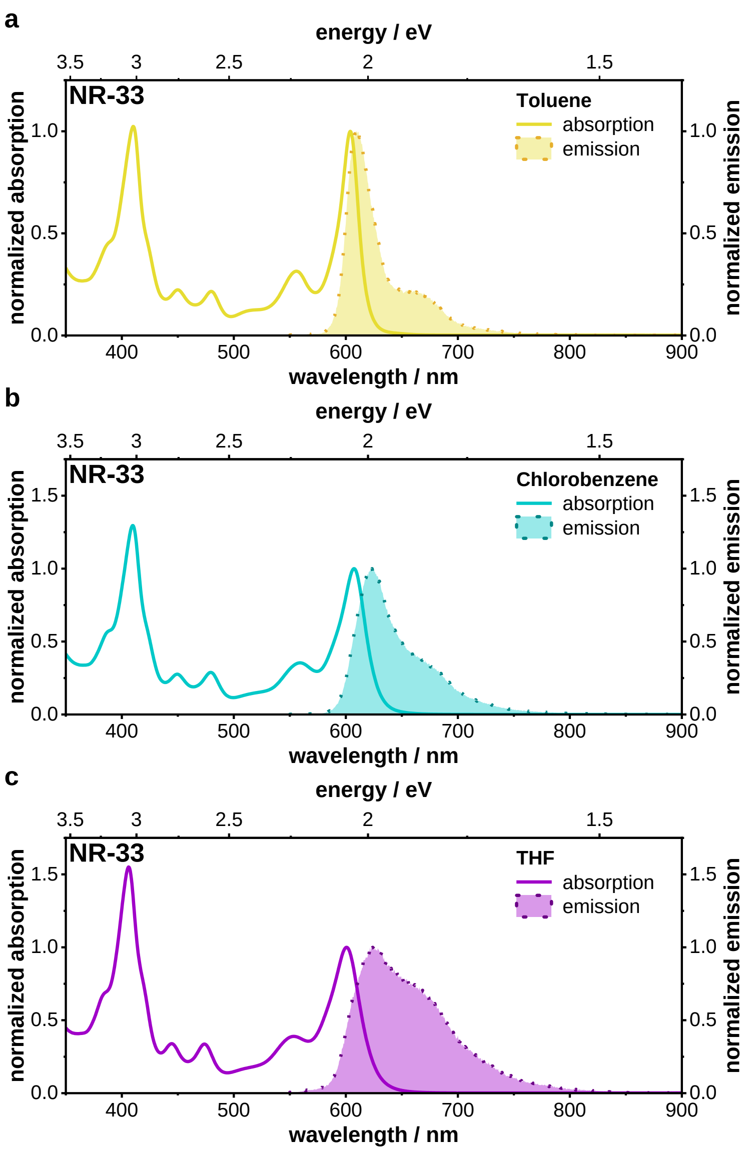

Figure S5 | Stokes shift plots. Normalized steady-state absorption spectra plotted along with normalized steady-state emission spectra after photoexcitation at 550 nm of **NR-33** in (a) toluene, (b) chlorobenzene, and (c) THF highlighting the Stokes shift. Absorption spectra were normalized with respect to their respective longest-wavelength absorption maximum.

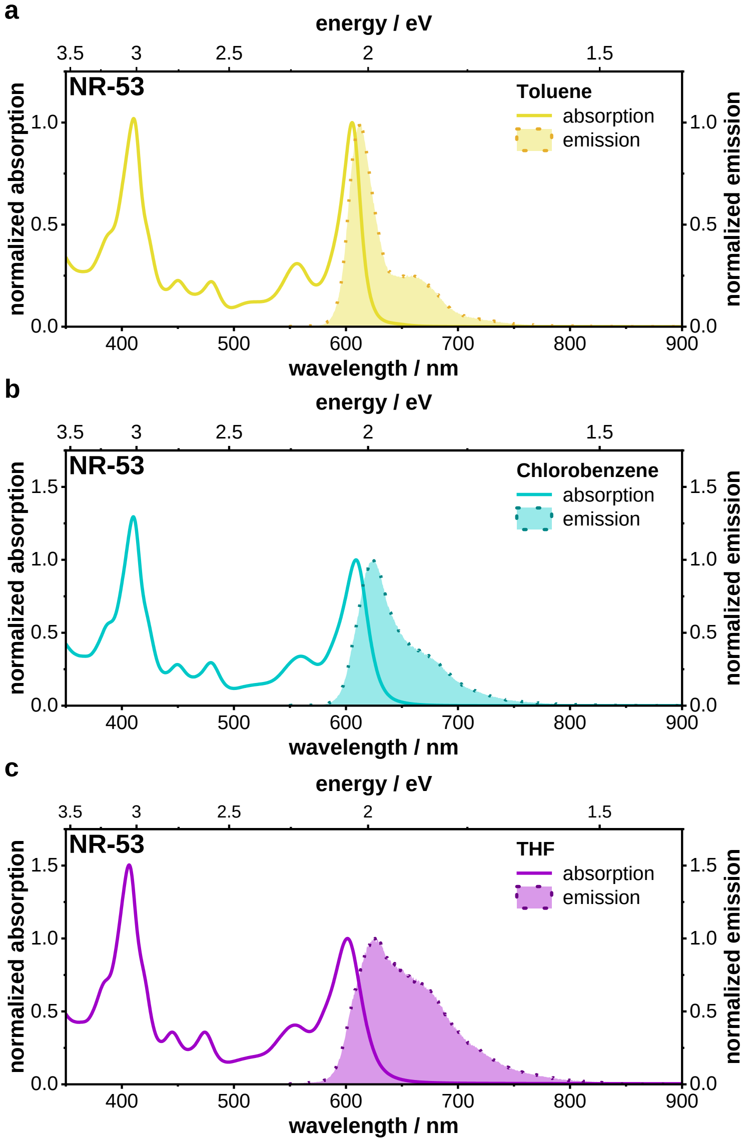

Figure S6 | Stokes shift plots. Normalized steady-state absorption spectra plotted along with normalized steady-state emission spectra after photoexcitation at 550 nm of **NR-53** in (a) toluene, (b) chlorobenzene, and (c) THF highlighting the Stokes shift. Absorption spectra were normalized with respect to their respective longest-wavelength absorption maximum.

Lippert-Mataga plot

In Lippert-Mataga plots, the change in dipole moment upon excitation is typically analyzed by relating the Stokes shift in wavenumbers ($\Delta\bar{\nu}$) to the orientation polarizability (Δf), which is a function of the refractive index (n) and dielectric constant (ε) of the respective solvent:

Equation 1:

$$\Delta\bar{\nu} = \frac{2\Delta f}{4\pi\varepsilon_0 hca_0^3}\,\Delta\mu^2 + \Delta\bar{\nu}^{\circ}$$

Equation 2:

$$\Delta f = \frac{\varepsilon - 1}{2\varepsilon + 1} - \frac{n^2 - 1}{2n^2 + 1}$$

Table S1 | Solvent properties. Refractive index n, dielectric constant ε, and calculated orientation polarizability of the various solvents relevant for the analysis using Lippert-Mataga plots.[1]

solvent[a]	n	ε	Δf
**Toluene**	1.49	2.38	0.014
**Chlorobenzene**	1.52	5.69	0.145
**THF**	1.41	7.52	0.210

[a] The values of the solvent properties were taken from reference 1.

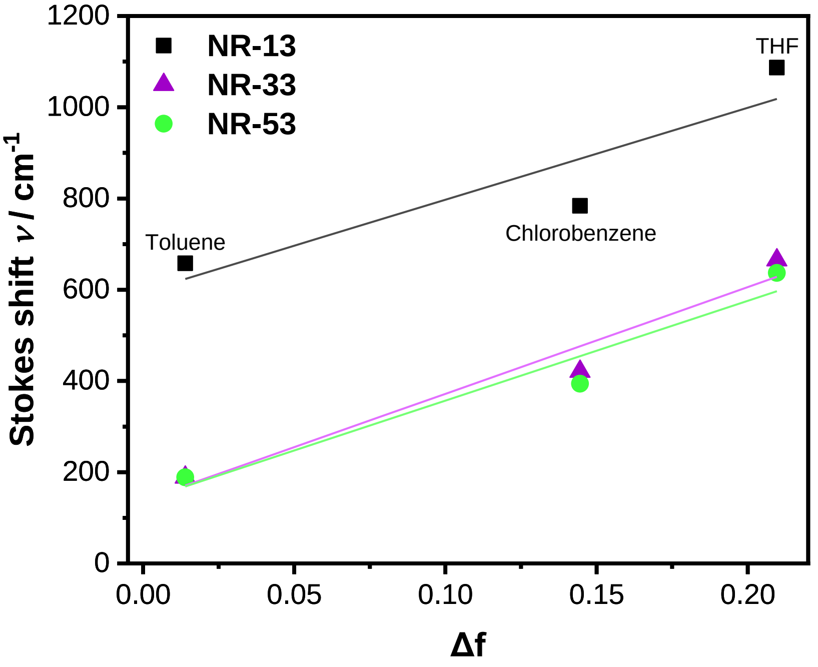

Figure S7 | Lippert-Mataga plots. Stokes shift as a function of the orientation polarizability of the solvent (Δf) for **NR-13** (black squares), **NR-33** (purple triangles), and **NR-53** (green circles). The lines correspond to the best linear fits of the data, using **Equation 1**, yielding a slope of $\frac{2}{4\pi\varepsilon_0 hca_0^3}$ $\boldsymbol{\Delta\mu}^2$= 2017 cm^{-1} for **NR-13** (R^2 = 0.83), $\frac{2}{4\pi\varepsilon_0 hca_0^3}$ $\boldsymbol{\Delta\mu}^2$= 2339 cm^{-1} for **NR-33** (R^2 = 0.96), and $\frac{2}{4\pi\varepsilon_0 hca_0^3}$ $\boldsymbol{\Delta\mu}^2$= 2187 cm^{-1} for **NR-53** (R^2 = 0.94). The Δf values were calculated using **Equation 2** *via* the dielectric constants and refractive indices of the solvents given in **Table S1**.

Changes in the dipole moment ($\Delta\mu$) for a compound upon excitation can be determined using the slope obtained from Lippert-Mataga plots (**Figure S7**) and the corresponding calculated Onsager radius (**Table S2**; see chapter 4 'Computational analysis' for details regarding its calculation).[2] For **NR-13**, $\Delta\mu$ is substantial, reaching a value of 10.4 D. Even larger values of $\Delta\mu$ are observed for **NR-33** (17.2 D) and **NR-53** (21.3 D), although the exact values should be interpreted with caution due to the deviation of the extended nanoribbons from the assumption of spherical point charges. Nonetheless, these results demonstrate a clear trend of a significant increase in the dipole moment for all three nanoribbons, indicative of strong charge-transfer character in the excited state.

Table S2 | Increase in dipole moment upon excitation. Onsager radii a_0 of the ground state equilibrium structures of **NR-13**, **NR-33**, and **NR-53**, which can be estimated by taking the molecular volume that can fit inside a contour of the 0.001 e^-/a_0^3 density, and the therewith calculated increase in dipole moment Δμ upon excitation.[2]

compound	a_0 / Å	Δμ / D
**NR-13**	8.13	10.4
**NR-33**	10.83	17.2
**NR-53**	12.78	21.3

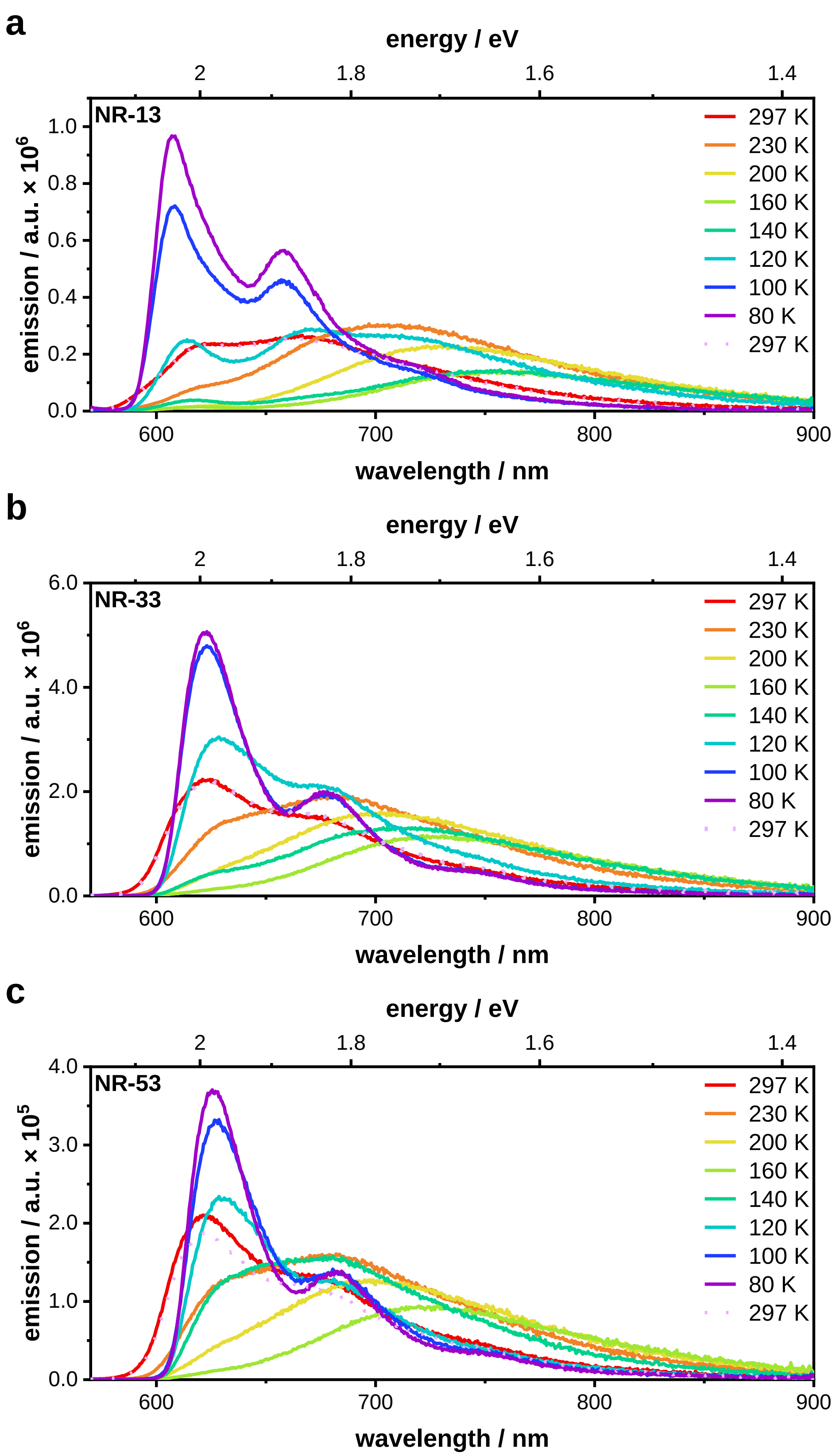

Figure S8 | Temperature-dependent steady-state emission in 2-MeTHF. Steady-state emission spectra of (a) **NR-13**, (b) **NR-33,** and (c) **NR-53** recorded in 2-MeTHF after photoexcitation at 550 nm at various temperatures (see figure legend for exact values).

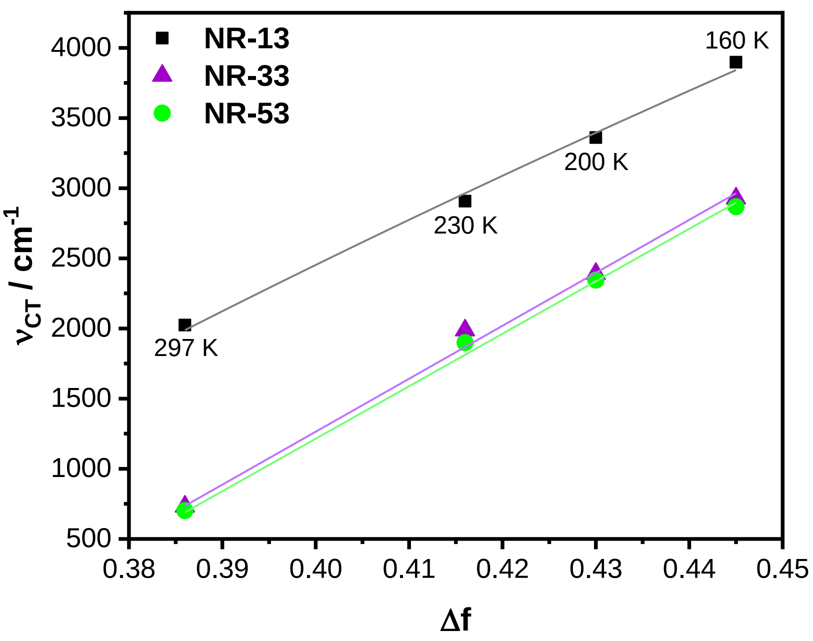

Figure S9 | Lippert-Mataga plots. Stokes shift (ν_{CT}) as a function of the temperature-dependent orientation polarizability (Δf) of 2-MeTHF for **NR-13** (black squares), **NR-33** (purple triangles), and **NR-53** (green circles). The lines correspond to the best linear fits of the data. The effective solvent polarity Δf values of 2-MeTHF at different temperatures are taken from reference 3.[3] Note that Δf increases upon lowering of the temperature.[4]

Table S3 | Summary of the steady-state absorption and emission properties. Steady-state absorption maxima of the β-band (λ_β) as well as the ρ-band (λ_ρ), corresponding molar extinction coefficients ε, emission maxima λ_{em}, the Stokes shift between absorption and emission maximum, fluorescence quantum yield Φ_F, and the optical band gap energy E_g^{opt}.

comp.	solvent	λ_β / nm	ε_β / M^{-1} cm^{-1}	λ_ρ / nm	ε_ρ / M^{-1} cm^{-1}	ratio ρ:β	λ_{em} / nm	Stokes shift / cm^{-1}	Φ_F / %	E_g^{opt} / eV
**NR-13**	**Toluene**	406	196,000	592	130,000	0.66	616	661	19	2.03
	**Chloro-benzene**	406	204,000	594	116,000	0.57	624	782	23	2.00
	**THF**	402	222,000	587	111,000	0.50	627	1089	17	2.02
**NR-33**	**Toluene**	410	522,000	604	510,000	0.98	611	194	26	2.00
	**Chloro-benzene**	410	505,000	608	390,000	0.77	624	419	31	1.97
	**THF**	406	558,000	601	360,000	0.65	626	661	26	1.98
**NR-53**	**Toluene**	411	847,000	606	830,000	0.98	613	186	24	2.00
	**Chloro-benzene**	410	868,000	609	670,000	0.77	624	395	27	1.97
	**THF**	407	828,000	602	550,000	0.66	626	637	20	1.97

An error margin of ± 3 % is implicit in the determination of the fluorescence quantum yields.

Table S4 | Temperature-dependent fluorescence quantum yields. Calculated fluorescence quantum yields (Φ_F) of **NR-13**, **NR-33**, and **NR-53** in 2-MeTHF after photoexcitation at 550 nm as a function of temperature.[a]

temperature / K	Φ_F / %		
	**NR-13**	**NR-33**	**NR-53**
**297 K**	17	26	20
**230 K**	19	31	22
**200 K**	14	26	18
**160 K**	8	19	13
**140 K**	9	22	19
**120 K**	8	32	17
**100 K**	16	31	20
**80 K**	17	30	20

[a] Calculated by comparing the integrated emission at each temperature to the integrated emission at room temperature (297 K). Deviations in the absorption with respect to temperature are taken into account in the calculations *via* **Figure S3**.

2. Time-resolved spectroscopy

2.1 Time-resolved emission

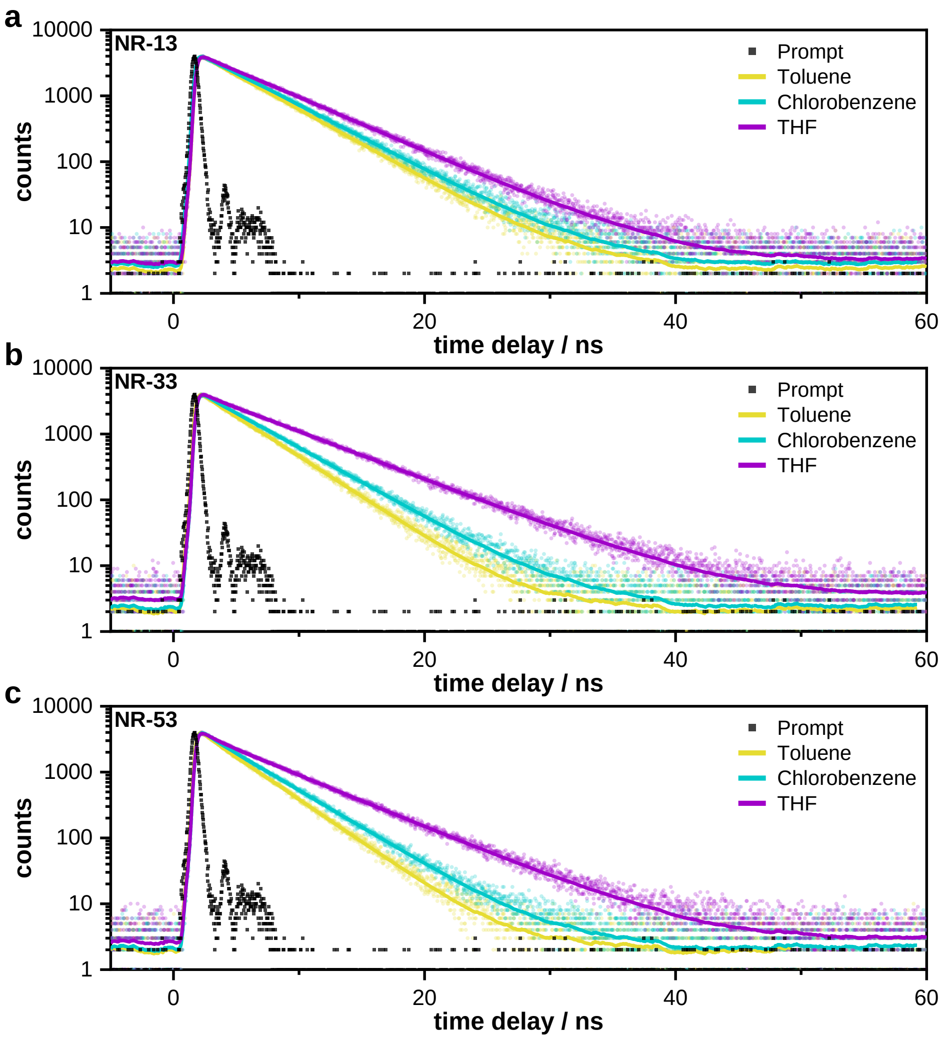

Figure S10 | Time-resolved emission. Time-resolved emission spectra of (a) **NR-13**, (b) **NR-33**, and (c) **NR-53** including their corresponding prompts (black scatter plot) and mono-exponential fits in argon-saturated solvents of varying polarity. Decays (of the respective emission maxima) and corresponding fits are assigned to the same color (see figure legend for exact details). All measurements were performed at room temperature after photoexcitation at 532 nm. The optical densities were approximately 0.1 at the excitation wavelength for all samples.

Table S5 | Time-resolved emission. Lifetimes (τ) and corresponding chi-square values (χ^2) of **NR-13**, **NR-33**, and **NR-53** obtained from time-correlated single photon counting experiments upon photoexcitation at 532 nm in various solvents at room temperature.

compound	solvent	τ / ns	χ^2
**NR-13**	**Toluene**	4.4	1.2
	**Chlorobenzene**	5.2	1.3
	**THF**	7.8	1.3
**NR-33**	**Toluene**	3.5	1.1
	**Chlorobenzene**	4.0	1.2
	**THF**	5.9	1.3
**NR-53**	**Toluene**	3.2	1.2
	**Chlorobenzene**	3.7	1.2
	**THF**	5.3	1.5

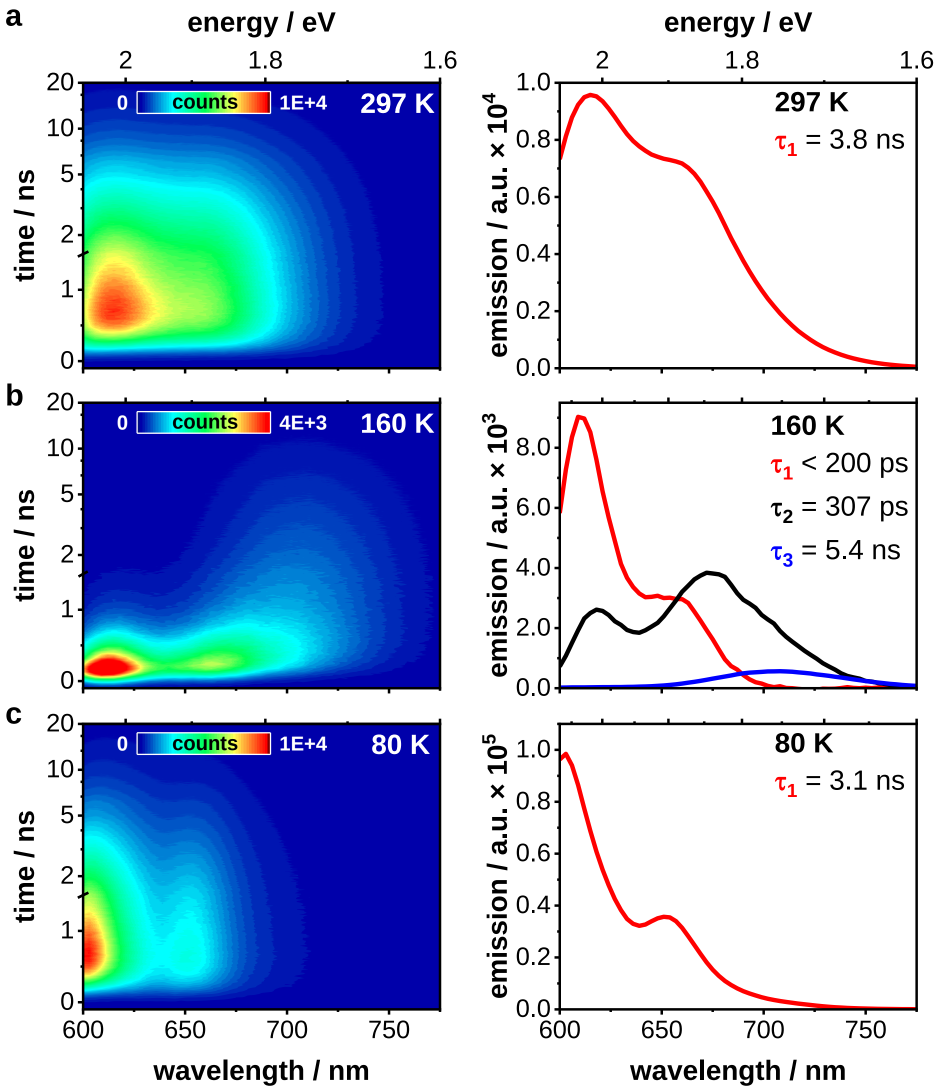

Figure S11 | Temperature-dependent time-resolved emission. Time-resolved emission 2D heat maps of **NR-13** along with their corresponding deconvoluted spectra obtained *via* global sequential analysis, recorded in argon-saturated 2-MeTHF after photoexcitation at 550 nm at (a) 297 K, (b) 160 K, and (c) 80 K. The optical density was approximately 0.1 at the excitation wavelength.

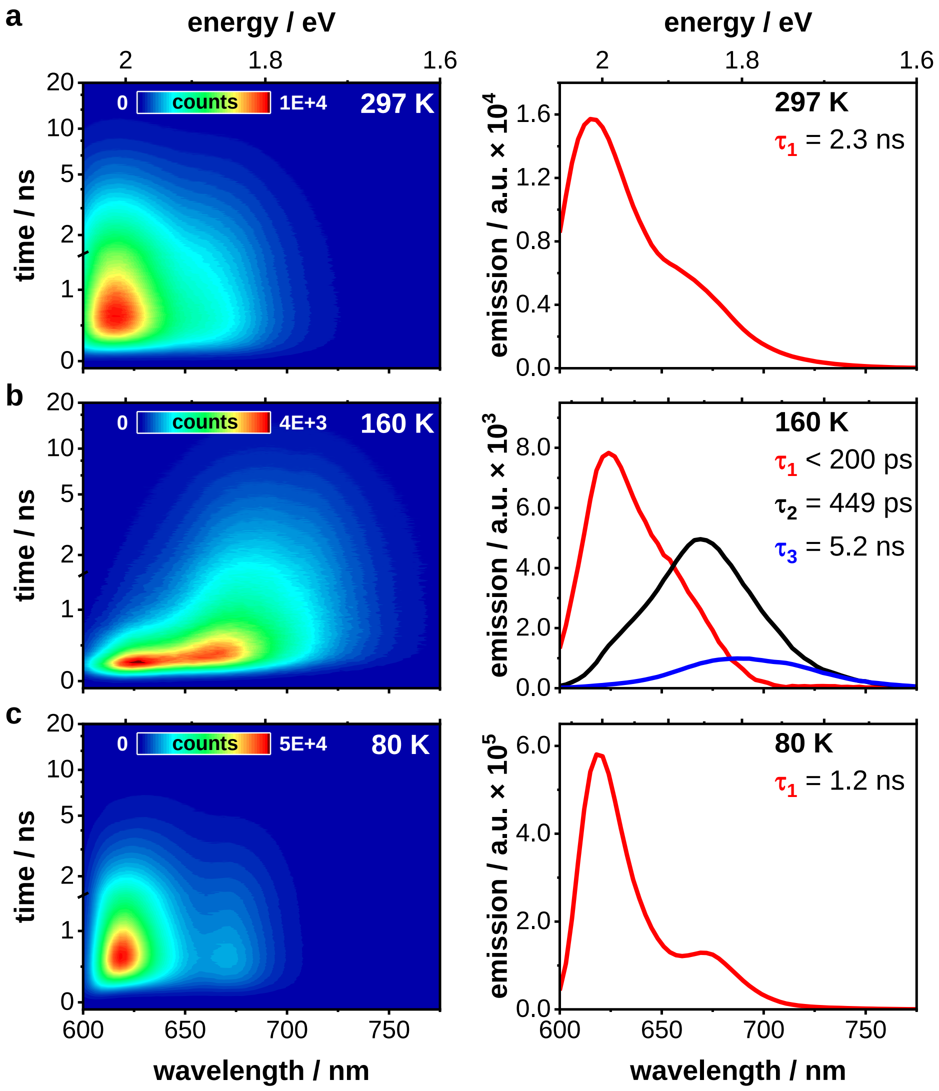

Figure S12 | Temperature-dependent time-resolved emission. Time-resolved emission 2D heat maps of **NR-53** along with their corresponding deconvoluted spectra obtained *via* global sequential analysis, recorded in argon-saturated 2-MeTHF after photoexcitation at 550 nm at (a) 297 K, (b) 160 K, and (c) 80 K. The optical density was approximately 0.1 at the excitation wavelength.

Table S6 | Temperature-dependent time-resolved emission. Lifetimes of the deconvoluted species of **NR-13**, **NR-33**, and **NR-53** obtained from temperature-dependent time-resolved emission experiments recorded in 2-MeTHF after photoexcitation at 550 nm at various temperatures.

compound	temperature	τ_1 / ps	τ_2 / ps	τ_3 / ns
	**297 K**	-	-	3.8
**NR-13**	**160 K**	< 200[a]	307	5.4
	**80 K**	-	-	3.1
	**297 K**	-	-	2.5
**NR-33**	**160 K**	< 200[a]	378	4.9
	**80 K**	-	-	1.8
	**297 K**	-	-	2.3
**NR-53**	**160 K**	< 200[a]	449	5.2
	**80 K**	-	-	1.2

[a] The lifetime of the short-lived component (τ_1) is below the resolution limit of the instrument and, thus, cannot be deconvoluted from the instrument response function.

2.2 Femtosecond transient absorption spectroscopy data

2.2.1 Femtosecond transient absorption spectroscopy experiments at room temperature

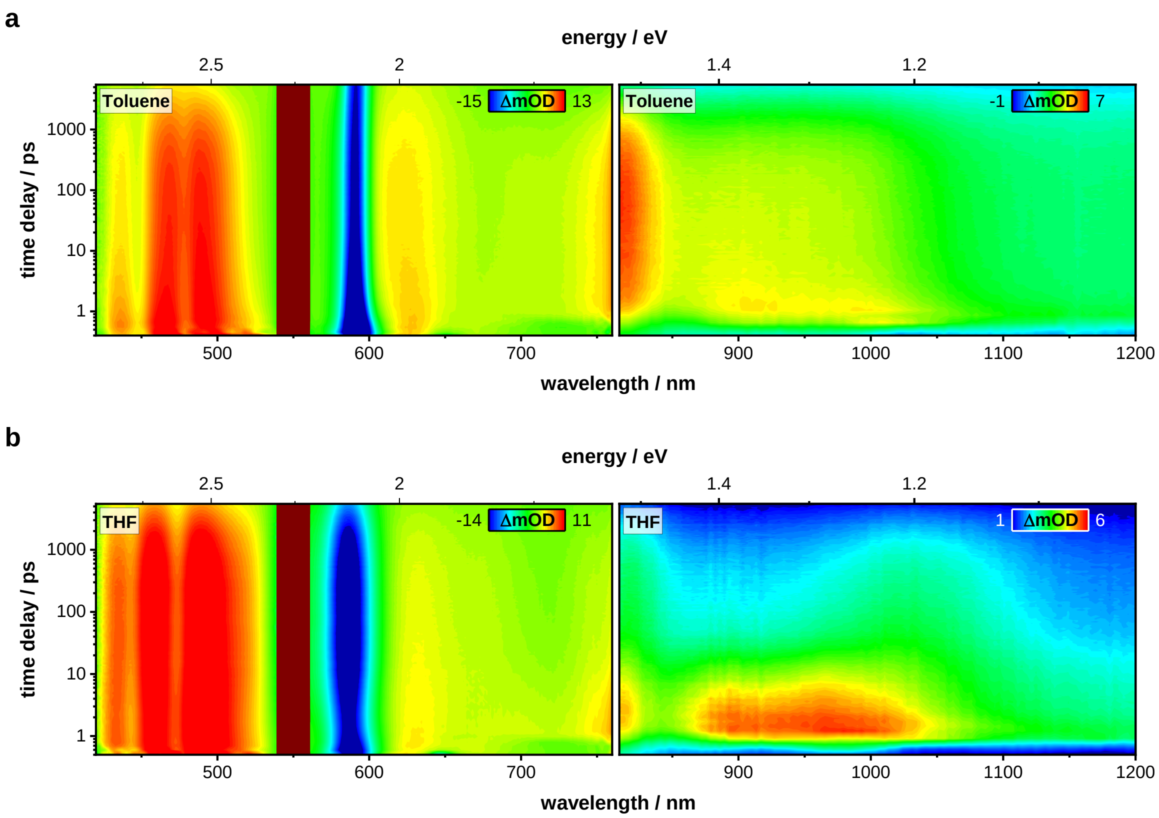

Figure S13 | Femtosecond transient absorption data of NR-13. Chirp- and zero-point-corrected differential transient absorption (TA) 2D heat maps of **NR-13** obtained from femtosecond TA experiments upon photoexcitation at 550 nm (500 nJ) in argon-saturated (a) toluene, and (b) THF at room temperature with various time delays between 0 to 5500 ps (corresponding single-wavelength kinetics are shown in the supporting information in **Figure S20**).

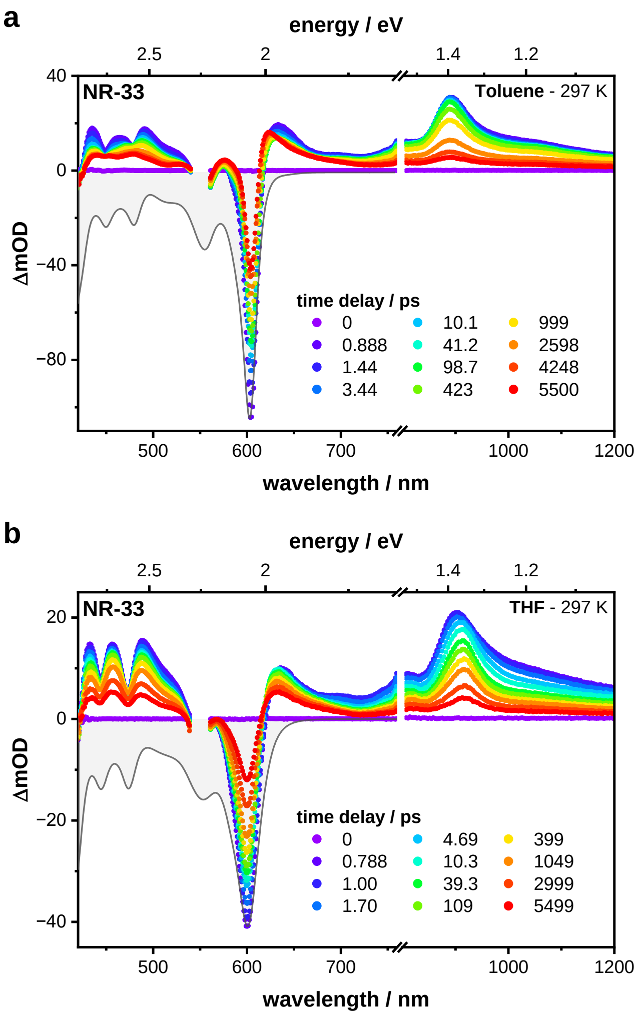

Figure S14 | Femtosecond transient absorption data of NR-33. Chirp- and zero-point-corrected differential transient absorption (TA) spectra of **NR-33** obtained from femtosecond TA experiments upon photoexcitation at 550 nm (500 nJ) in argon-saturated (a) toluene, and (b) THF at room temperature with various time delays between 0 to 5500 ps (corresponding single-wavelength kinetics are shown in the supporting information in **Figure S22**).

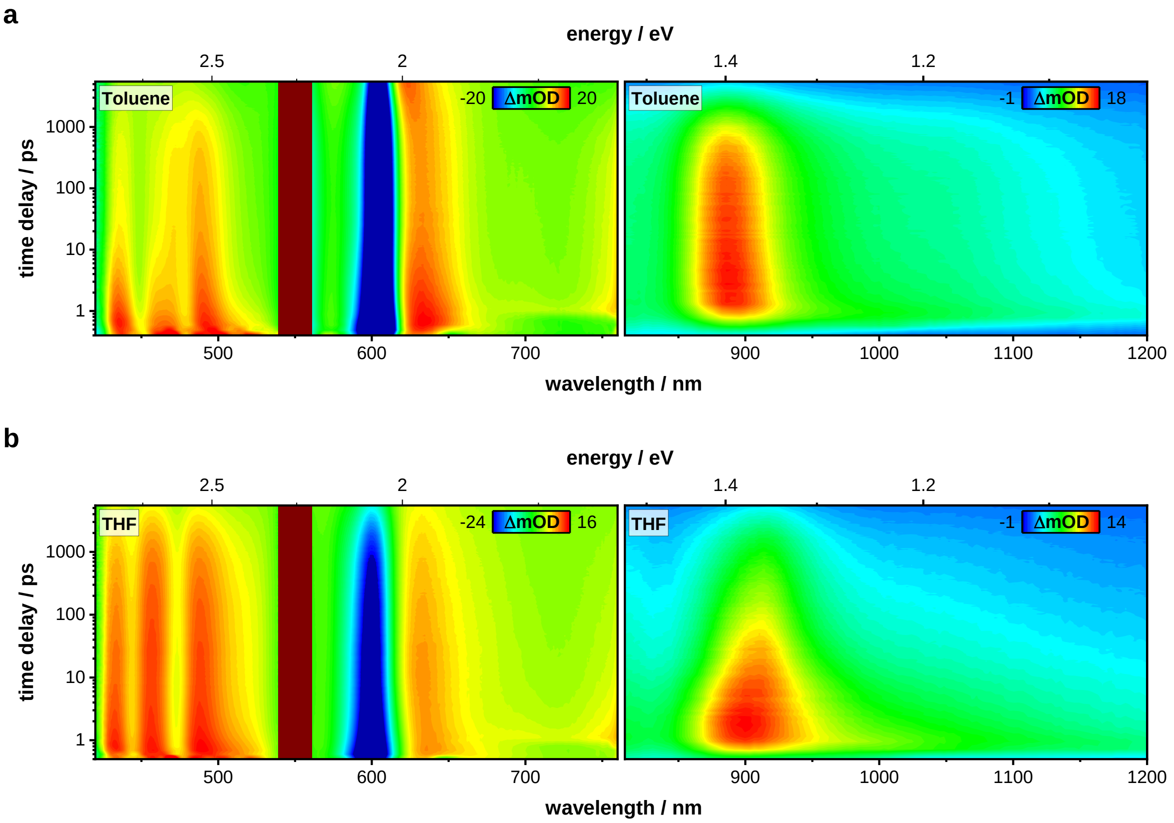

Figure S15 | Femtosecond transient absorption data of NR-33. Chirp- and zero-point-corrected differential transient absorption (TA) 2D heat maps of **NR-33** obtained from femtosecond TA experiments upon photoexcitation at 550 nm (500 nJ) in argon-saturated (a) toluene, and (b) THF at room temperature with various time delays between 0 to 5500 ps (corresponding single-wavelength kinetics are shown in the supporting information in **Figure S22**).

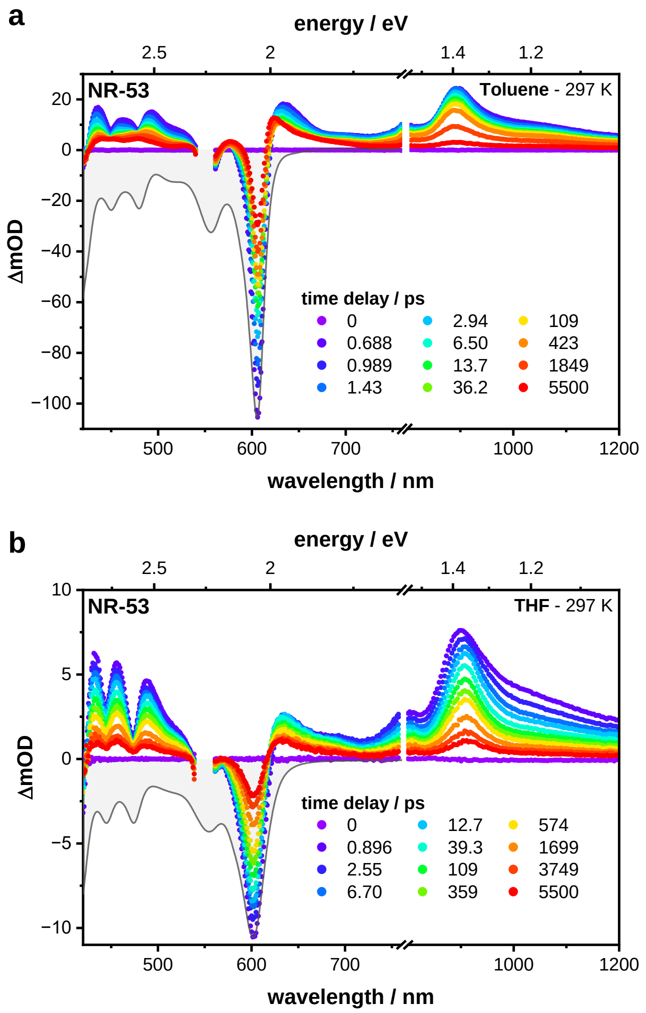

Figure S16 | Femtosecond transient absorption data of NR-53. Chirp- and zero-point-corrected differential transient absorption (TA) spectra of **NR-53** obtained from femtosecond TA experiments upon photoexcitation at 550 nm (500 nJ) in argon-saturated (a) toluene, and (b) THF at room temperature with various time delays between 0 to 5500 ps (corresponding single-wavelength kinetics are shown in the supporting information in **Figure S24)**.

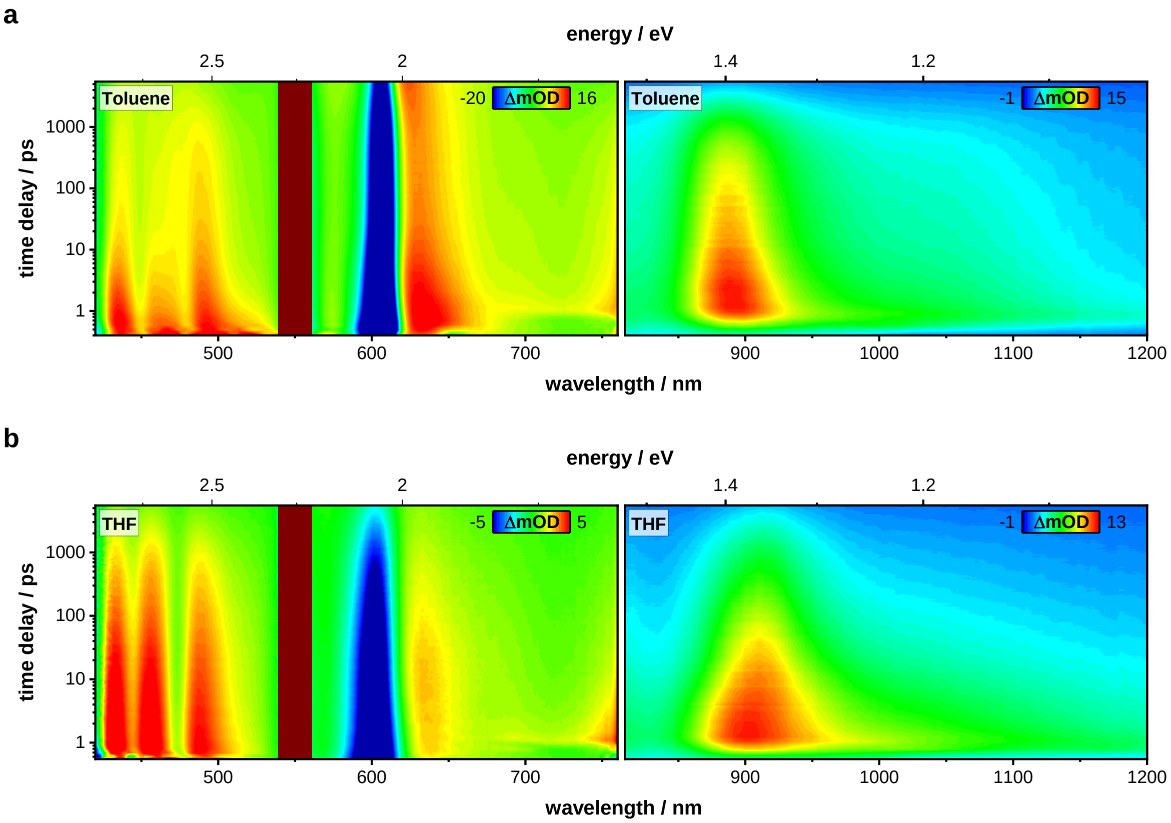

Figure S17 | Femtosecond transient absorption data of NR-53. Chirp- and zero-point-corrected differential transient absorption (TA) 2D heat maps of **NR-53** obtained from femtosecond TA experiments upon photoexcitation at 550 nm (500 nJ) in argon-saturated (a) toluene, and (b) THF at room temperature with various time delays between 0 to 5500 ps (corresponding single-wavelength kinetics are shown in the supporting information in **Figure S24)**.

model used to fit the fsTA data of **NR-13** in

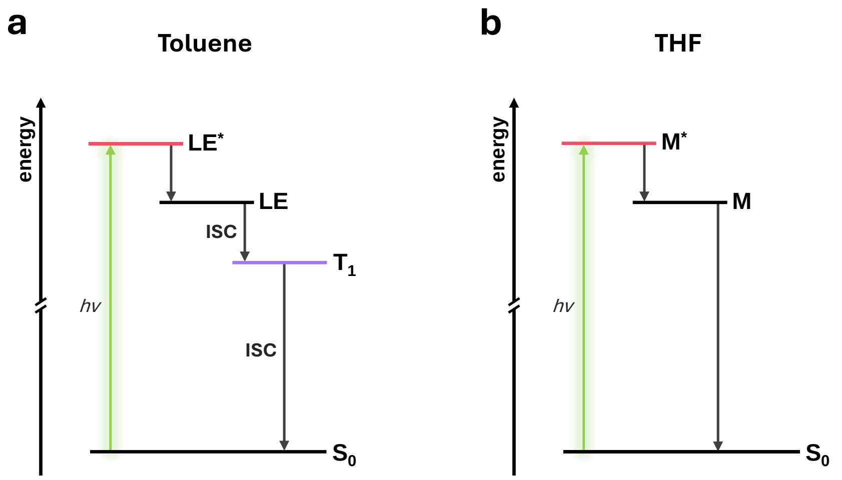

Figure S18 | Kinetic model employed to fit the femtosecond transient absorption data of NR-13 at room temperature. (a) The deactivation cascade of **NR-13** in low-polarity solvents, such as toluene, is modeled using a three-species sequential model, in which an electronic state with predominant locally excited character (LE*) is initially populated upon photoexcitation at 550 nm. Following solvent and vibrational relaxation yielding a relaxed locally excited state (LE), **NR-13** undergoes intersystem crossing (ISC) resulting in the population of the first triplet excited state (T_1) before the deactivation process is concluded. (b) Conversely, in solvents with increased polarity, such as THF, the deactivation cascade of **NR-13** is modeled using only a two-species sequential model, as the lifetime of the initially populated state—now with larger charge-transfer character and, thus, described as a mixed electronic state (M)—becomes longer in more polar environments to the extent that any following species cannot be deconvoluted within the achievable time delays of the femtosecond transient absorption setup.

model used to fit the fsTA data of **NR-33** and **NR-53** in

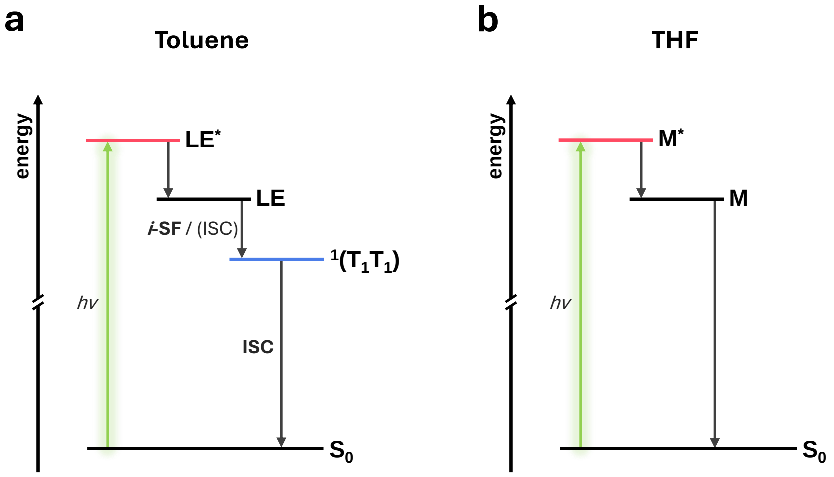

Figure S19 | Kinetic model employed to fit the femtosecond transient absorption data of NR-33 and NR-53 at room temperature. (a) The deactivation cascade of **NR-33** and **NR-53** in low-polarity solvents, namely toluene, is modeled using a three-species sequential model, in which a mixed electronic state with predominant locally excited character (LE*) or some charge-transfer contributions (M*) is initially populated upon photoexcitation at 550 nm. Solvent and vibrational relaxation yields a relaxed mixed electronic state (LE and M, respectively), which is subsequently converted into the singlet correlated triplet pair state $^1(T_1T_1)$ *via* intramolecular singlet fission (*i*-SF) before the deactivation process is concluded. A parallel formation of triplet excited states with some minor contribution stemming from the intersystem crossing (ISC) channel cannot be precluded but is not modeled due to challenges differentiating between *via* ISC and *i*-SF generated triplets spectroscopically and, consequently, a lack of experimental data that would allow for an accurate modeling of such. (b) Conversely, in solvents with increased polarity, such as THF, the deactivation cascade of **NR-33** and **NR-53** is modeled using only a two-species sequential model, as the lifetime of the initially populated state—now with larger charge-transfer character and, thus, described as a mixed electronic state (M)—becomes longer in more polar environments to the extent that any following species cannot be deconvoluted within the achievable time delays of the femtosecond transient absorption setup.

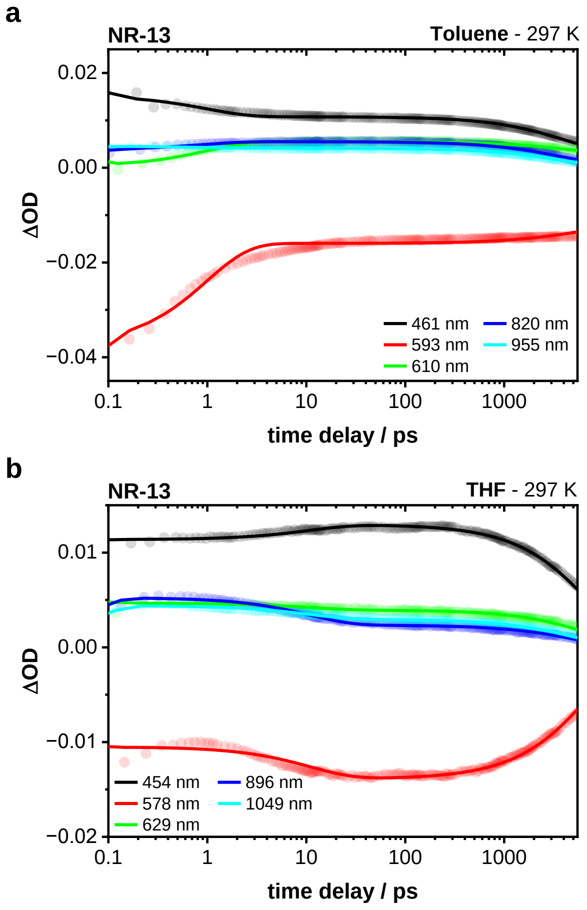

Figure S20 | Global analysis of the femtosecond transient absorption data of NR-13. Raw data single-wavelength kinetics of selected wavelengths (scatter plot) and fits to the data (solid line) of **NR-13** in (a) toluene, and (b) THF at room temperature shown in **Figure 3** and **S13**. Raw data and corresponding fit for a particular wavelength are assigned to the same color (see figure legend for exact details).

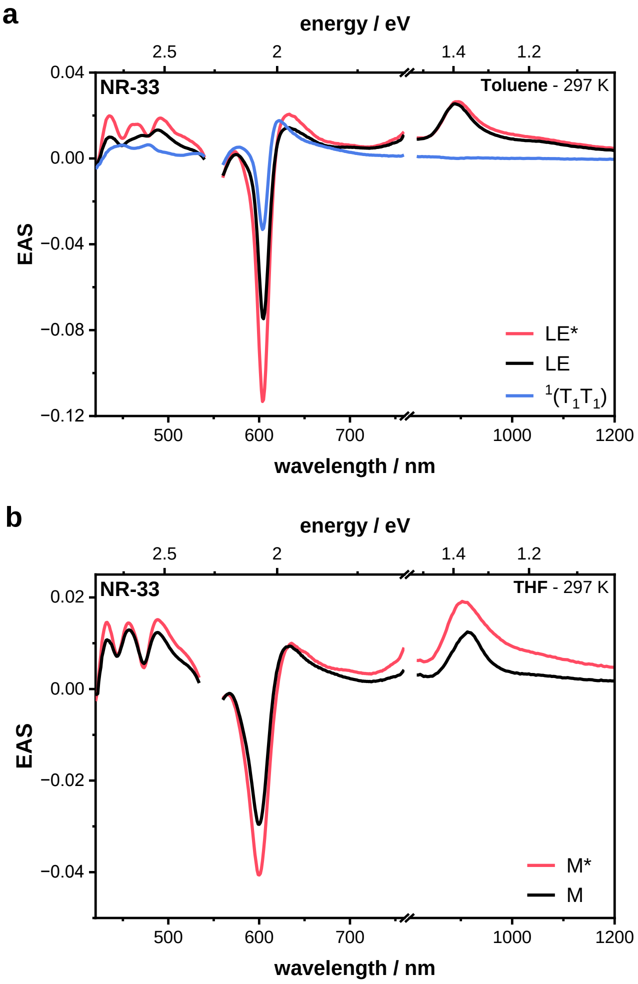

Figure S21 │ Global analysis of the femtosecond transient absorption data of NR-33. Deconvoluted evolution-associated spectra (EAS) of the initially populated mixed electronic state (red), which has predominantly locally excited character (LE*) or includes some charge-transfer contributions (M*), the subsequent solvent- and vibrationally-relaxed mixed electronic states (LE and M, respectively; black), and the singlet correlated triplet pair state ($^1(T_1T_1)$; blue) as obtained by global analysis of the transient absorption data of **NR-33** in argon-saturated (a) toluene, and (b) THF shown in **Figure S14** using the kinetic model given in **Figure S19**.

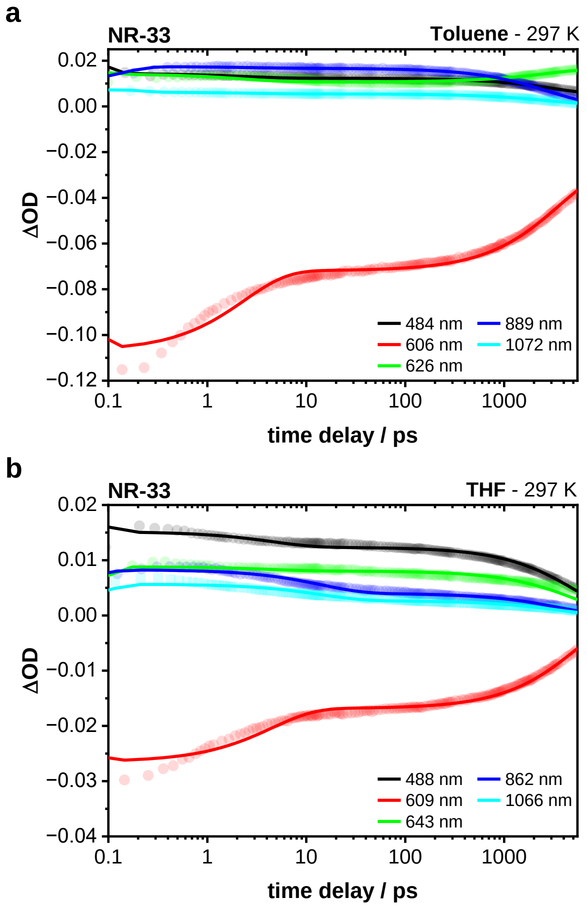

Figure S22 | Global analysis of the femtosecond transient absorption data of NR-33. Raw data single-wavelength kinetics of selected wavelengths (scatter plot) and fits to the data (solid line) of **NR-33** in (a) toluene, and (b) THF at room temperature shown in **Figure S14**. Raw data and corresponding fit for a particular wavelength are assigned to the same color (see figure legend for exact details).

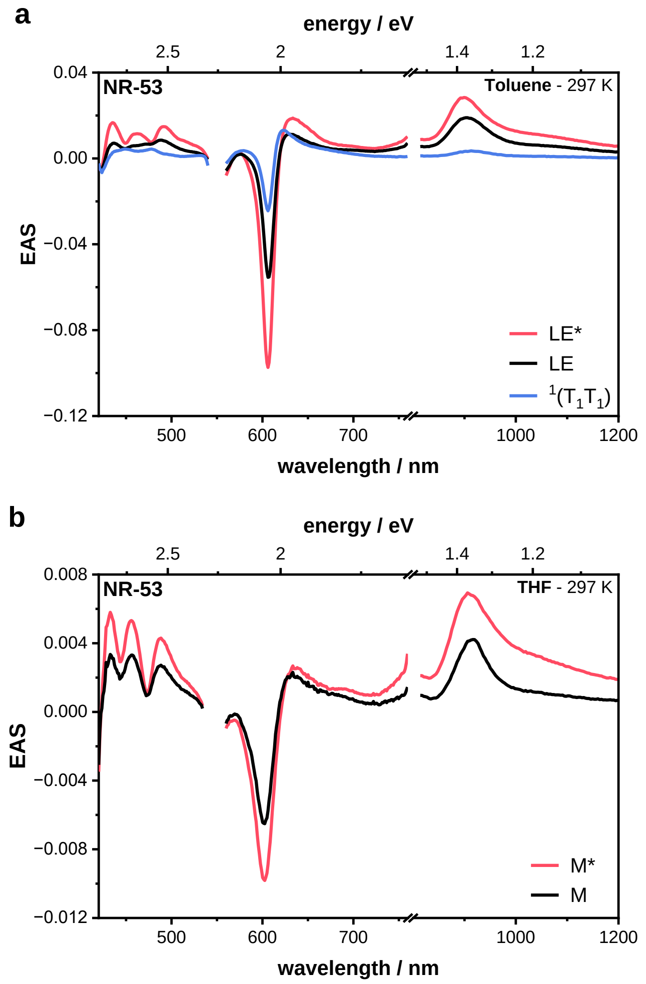

Figure S23 | Global analysis of the femtosecond transient absorption data of NR-53. Deconvoluted evolution-associated spectra (EAS) of the initially populated mixed electronic state (red), which has predominantly locally excited character (LE*) or includes some charge-transfer contributions (M*), the subsequent solvent- and vibrationally-relaxed mixed electronic states (LE and M, respectively; black), and the singlet correlated triplet pair state ($^1(T_1T_1)$; blue) as obtained by global analysis of the transient absorption data of **NR-53** in argon-saturated (a) toluene, and (b) THF shown in **Figure S16** using the kinetic model given in **Figure S19**.

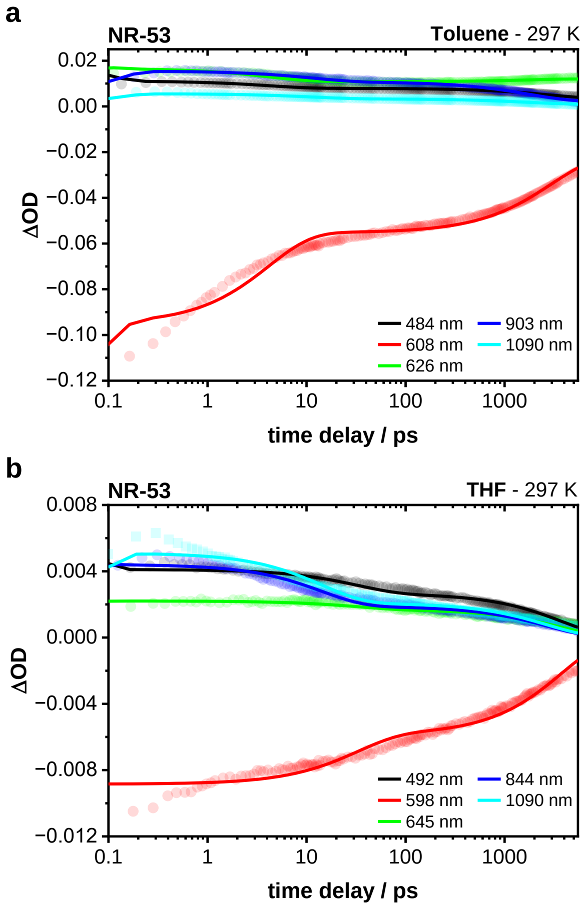

Figure S24 | Global analysis of the femtosecond transient absorption data of NR-53. Raw data single-wavelength kinetics of selected wavelengths (scatter plot) and fits to the data (solid line) of **NR-53** in (a) toluene, and (b) THF at room temperature shown in **Figure S16**. Raw data and corresponding fit for a particular wavelength are assigned to the same color (see figure legend for exact details).

A summary of all lifetimes obtained by global analysis of the femtosecond transient absorption data at room temperature is given in **Table S8**.

2.2.2 Femtosecond transient absorption spectroscopy experiments at cryogenic temperatures

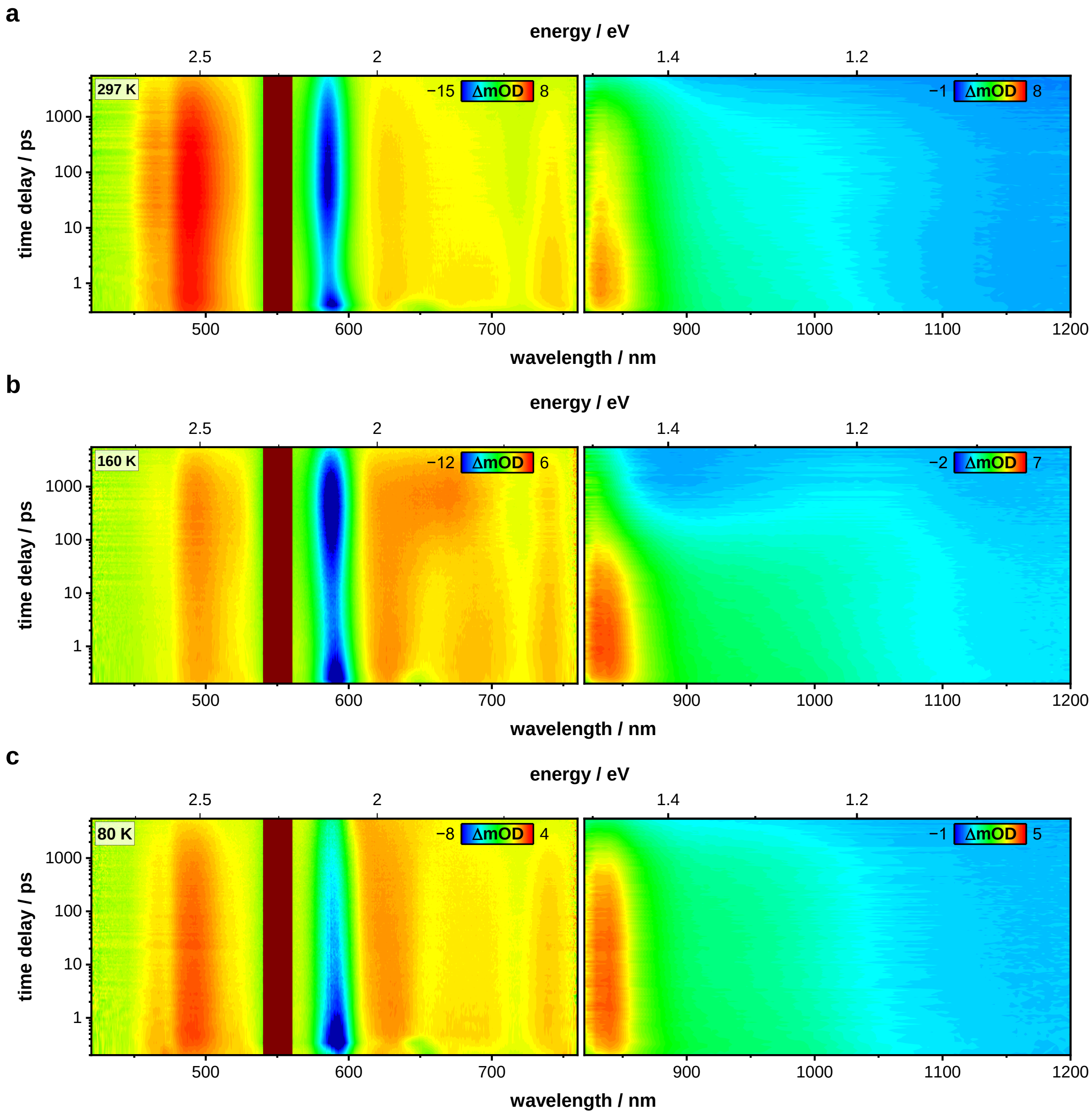

Figure S25 │ Temperature-dependent femtosecond transient absorption data of NR-13. Chirp- and zero-point-corrected differential transient absorption (TA) 2D heat maps of **NR-13** obtained from femtosecond TA experiments upon photoexcitation at 550 nm (200–50 nJ) in argon-saturated 2-MeTHF at (a) 297 K, (b) 160 K, and (c) 80 K with various time delays between 0 to 5500 ps (corresponding single-wavelength kinetics are shown in the supporting information in **Figure S33**).

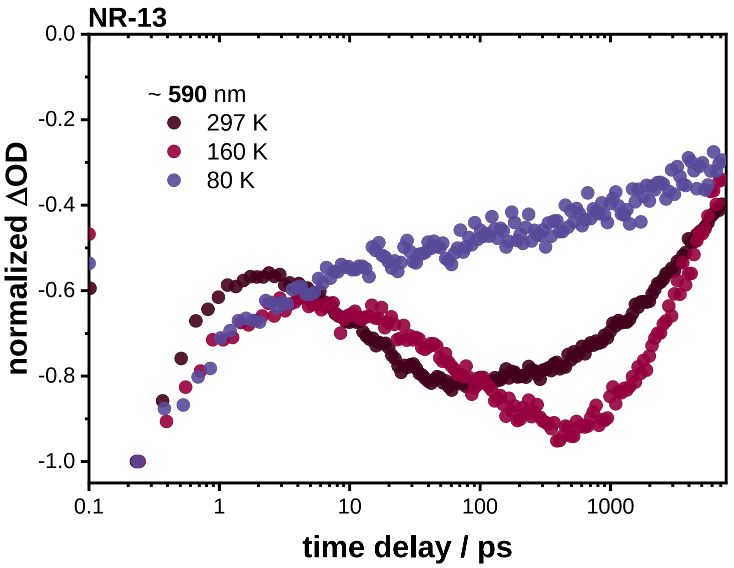

Figure S26 | Temperature-dependent excited state dynamics of NR-13. Comparison of the single-wavelength kinetics of the ρ-band of **NR-13**, that is, around 590 nm, obtained from femtosecond transient absorption experiments upon photoexcitation at 550 nm (200–50 nJ) with various time delays between 0 to 5500 ps in 2-MeTHF at various temperatures (see figure legend for exact values) highlighting the suppressed intramolecular charge-transfer formation in frozen solvent glass.

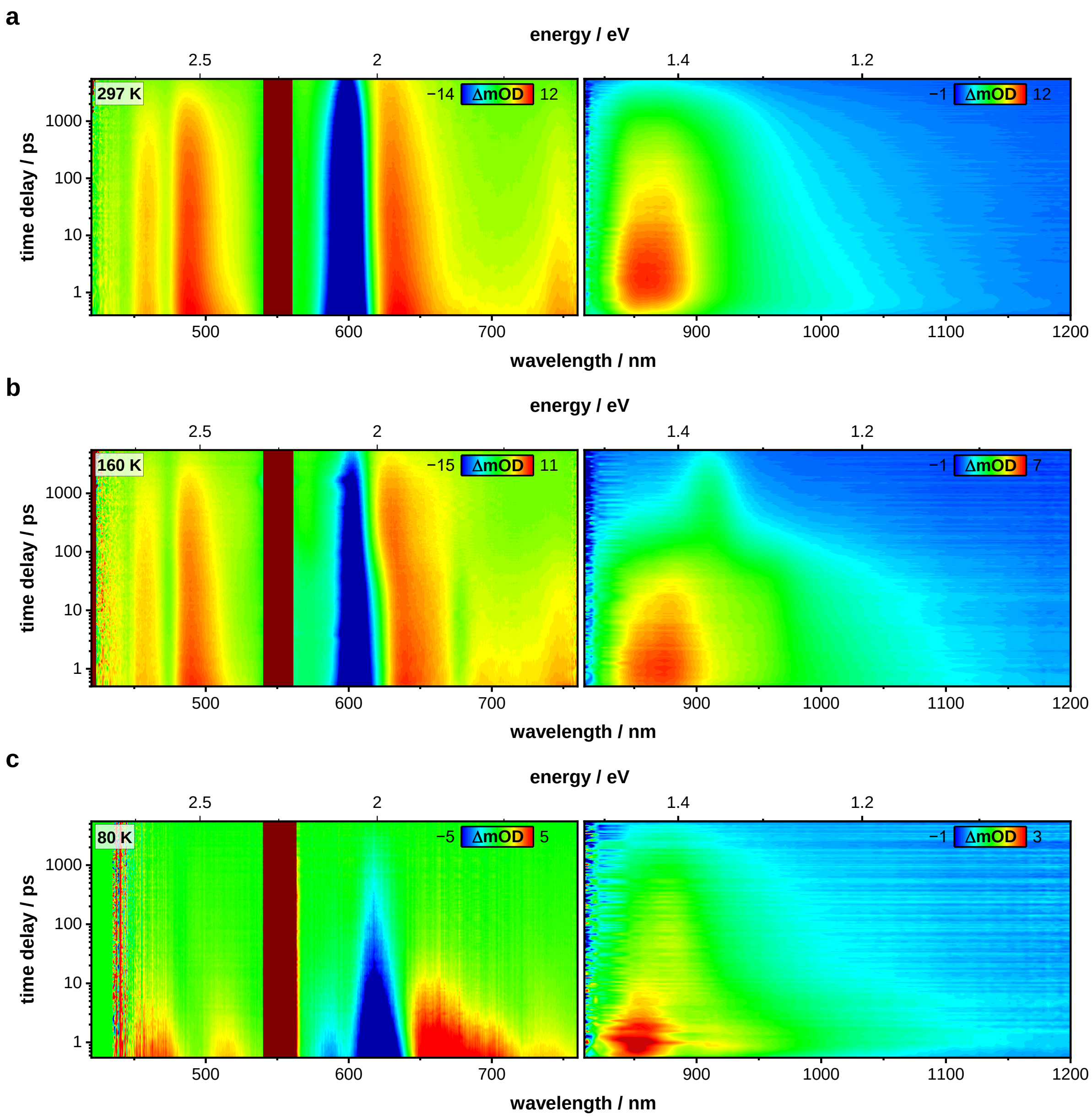

Figure S27 | Temperature-dependent femtosecond transient absorption data of NR-33. Chirp- and zero-point-corrected differential transient absorption (TA) 2D heat maps of **NR-33** obtained from femtosecond TA experiments upon photoexcitation at 550 nm (200–50 nJ) in argon-saturated 2-MeTHF at (a) 297 K, (b) 160 K, and (c) 80 K with various time delays between 0 to 5500 ps (corresponding single-wavelength kinetics are shown in the supporting information in **Figure S35**).

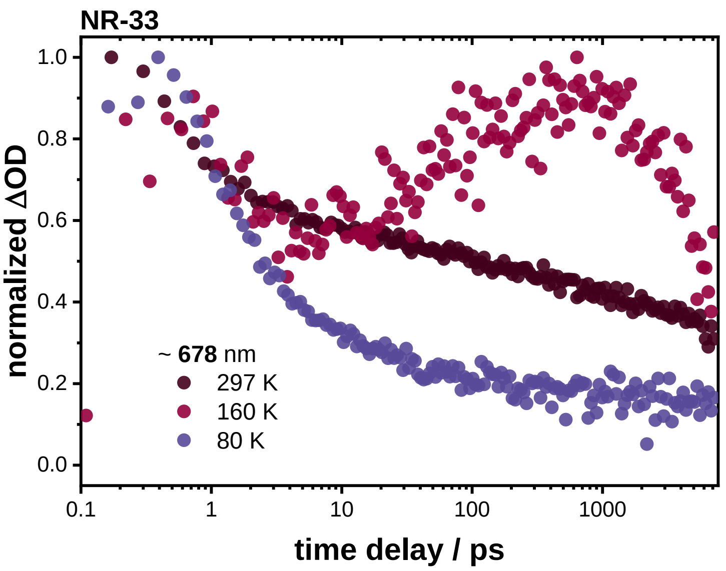

Figure S28 | Temperature-dependent excited state dynamics of NR-33. Comparison of the single-wavelength kinetics of **NR-33** at 678 nm obtained from femtosecond transient absorption experiments upon photoexcitation at 550 nm (200–50 nJ) with various time delays between 0 to 5500 ps in 2-MeTHF at various temperatures (see figure legend for exact values) highlighting the suppressed intramolecular charge-transfer formation in frozen solvent glass. For **NR-33**, the ground-state bleaching was not compared as features at 678 nm are more pronounced and, thus, allow for an easier comparison.

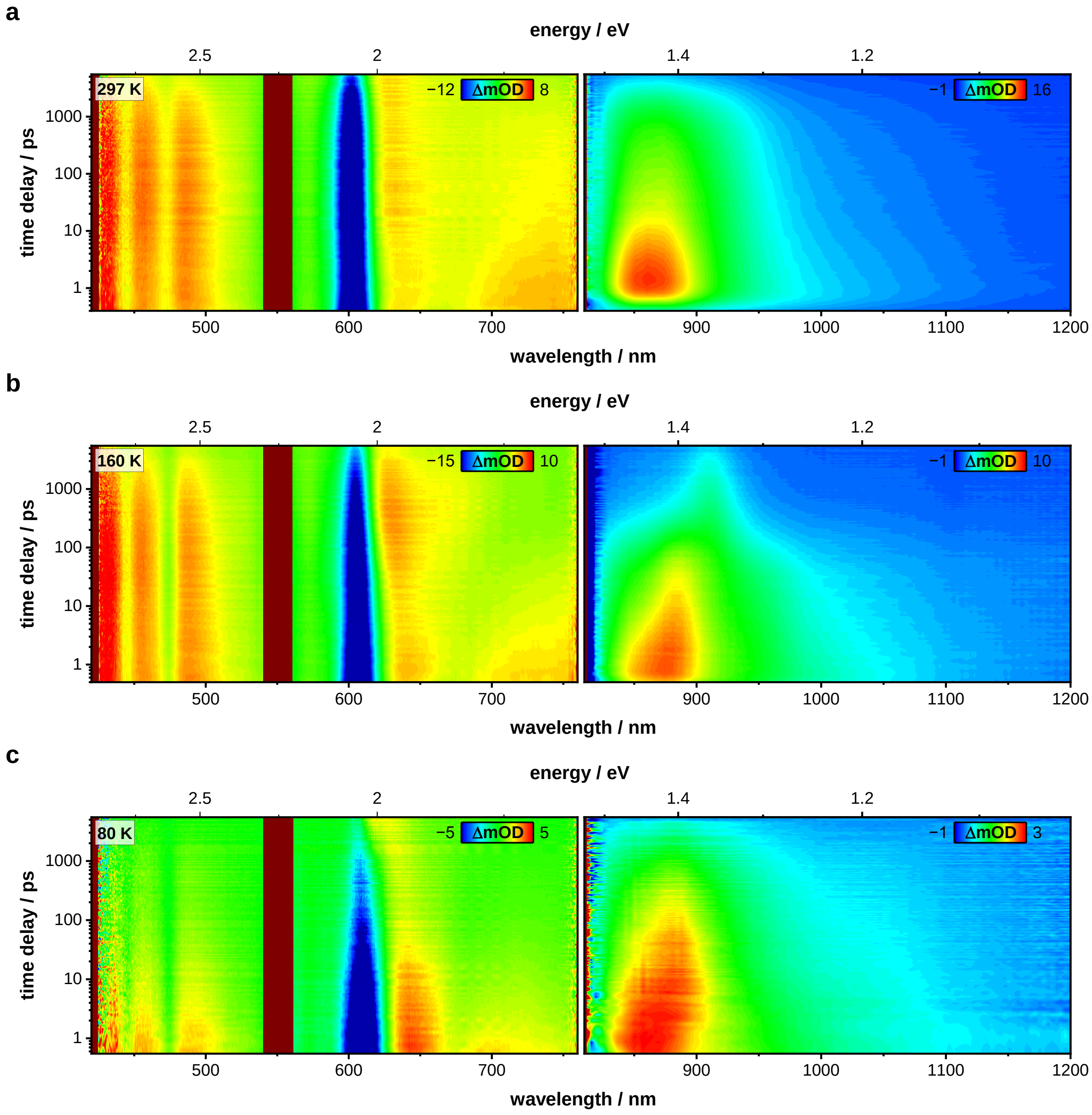

Figure S29 | Temperature-dependent femtosecond transient absorption data of NR-53. Chirp- and zero-point-corrected differential transient absorption (TA) 2D heat maps of **NR-53** obtained from femtosecond TA experiments upon photoexcitation at 550 nm (200–50 nJ) in argon-saturated 2-MeTHF at (a) 297 K, (b) 160 K, and (c) 80 K with various time delays between 0 to 5500 ps (corresponding single-wavelength kinetics are shown in the supporting information in **Figure S37**).

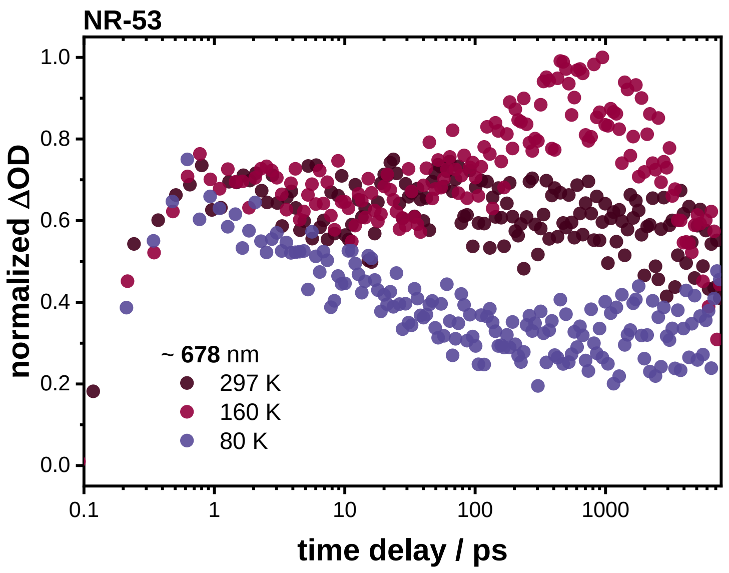

Figure S30 | Temperature-dependent excited state dynamics of NR-53. Comparison of the single-wavelength kinetics of **NR-53** at 678 nm obtained from femtosecond transient absorption experiments upon photoexcitation at 550 nm (200–50 nJ) with various time delays between 0 to 5500 ps in 2-MeTHF at various temperatures (see figure legend for exact values) highlighting the suppressed intramolecular charge-transfer formation in frozen solvent glass. For **NR-53**, the ground-state bleaching was not compared as features at 678 nm are more pronounced and, thus, allow for an easier comparison.

model used to fit the fsTA data of all three nanoribbons in **2-MeTHF** at

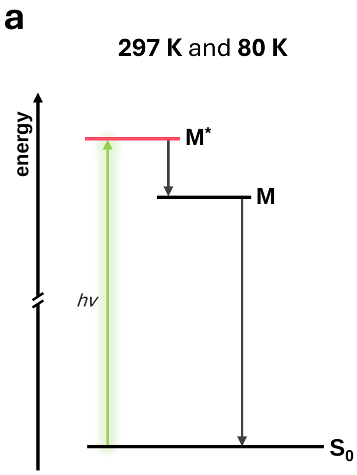

Figure S31 | Kinetic model employed to fit the temperature-dependent femtosecond transient absorption data of all three nanoribbons. (a) The deactivation cascade of **NR-13**, **NR-33**, and **NR-53** in 2-MeTHF at 297 and 80 K is modeled using a two-species sequential model, in which a mixed electronic state (M*), that is, a mixture of a diabatic locally excited and charge-transfer state, is initially populated upon photoexcitation at 550 nm. Solvent and vibrational relaxation yields a relaxed mixed electronic state (M) before the deactivation process is concluded. Any additional species cannot be deconvoluted within the achievable time delays of the fsTA setup. (b) In contrast, the deactivation cascade of cascade of **NR-13**, **NR-33**, and **NR-53** at 160 K is modeled using a three-species sequential model. For all three nanoribbons, initial excitation into M* is followed by solvent and structural reorganization which leads to the evolution of the admixture to produce a pure, diabatic intramolecular charge-transfer (CT*), which subsequently relaxes to produce CT.

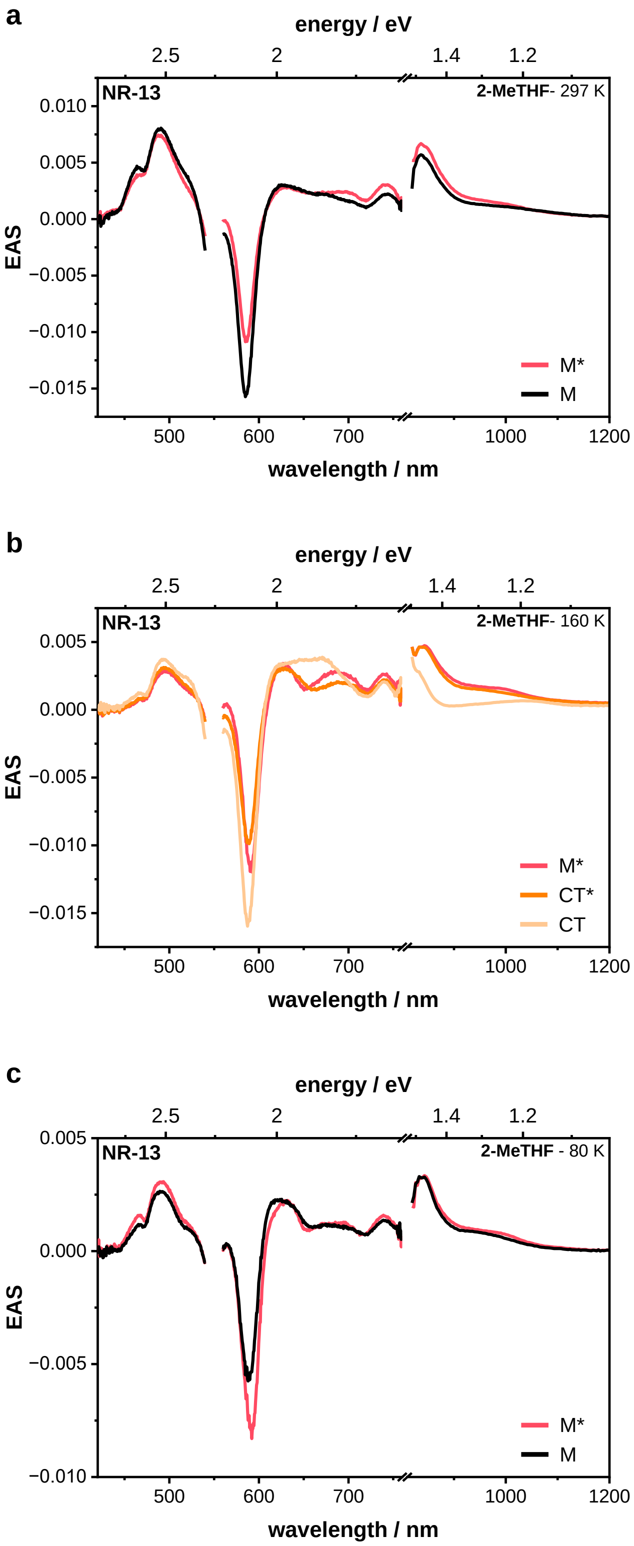

Figure S32 | Global analysis of the temperature-dependent femtosecond transient absorption data of NR-13. Deconvoluted evolution-associated spectra (EAS) of the mixed electronic state (M*, red), the subsequent solvent- and vibrationally-relaxed mixed electronic state (M; black), the pure (diabatic) intramolecular charge-transfer state (CT*; dark orange), and the structurally- and solvent-relaxed CT (light orange) as obtained by global analysis of the transient absorption data of **NR-13** in 2-MeTHF at (a) 297 K, (b) 160 K, and (c) 80 K shown in **Figure S25** using the kinetic model given in **Figure S31**.

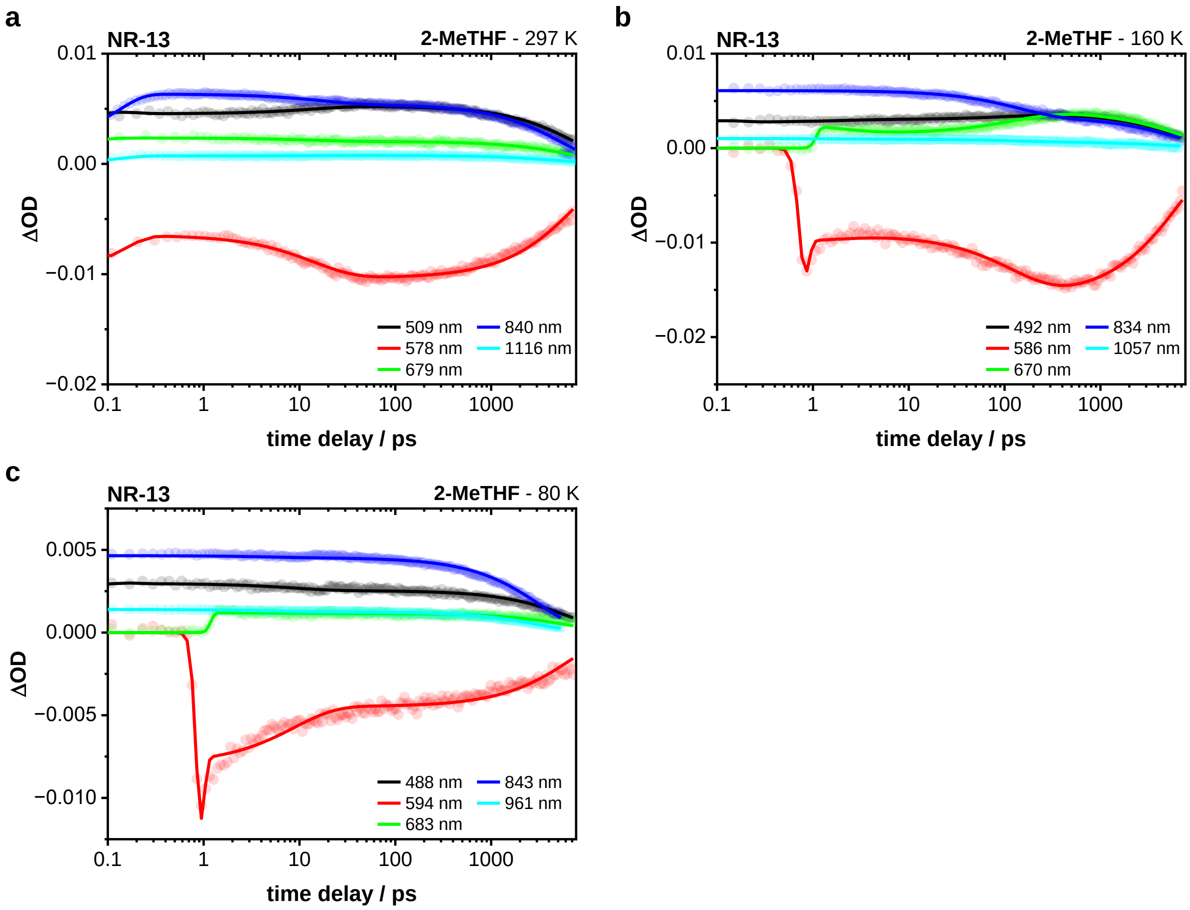

Figure S33 | Global analysis of the temperature-dependent femtosecond transient absorption data of NR-13. Raw data single-wavelength kinetics of selected wavelengths (scatter plot) and fits to the data (solid line) of **NR-13** in 2-MeTHF at (a) 297 K, (b) 160 K, and (c) 80 K shown in **Figure S25**. Raw data and corresponding fit for a particular wavelength are assigned to the same color (see figure legend for exact details).

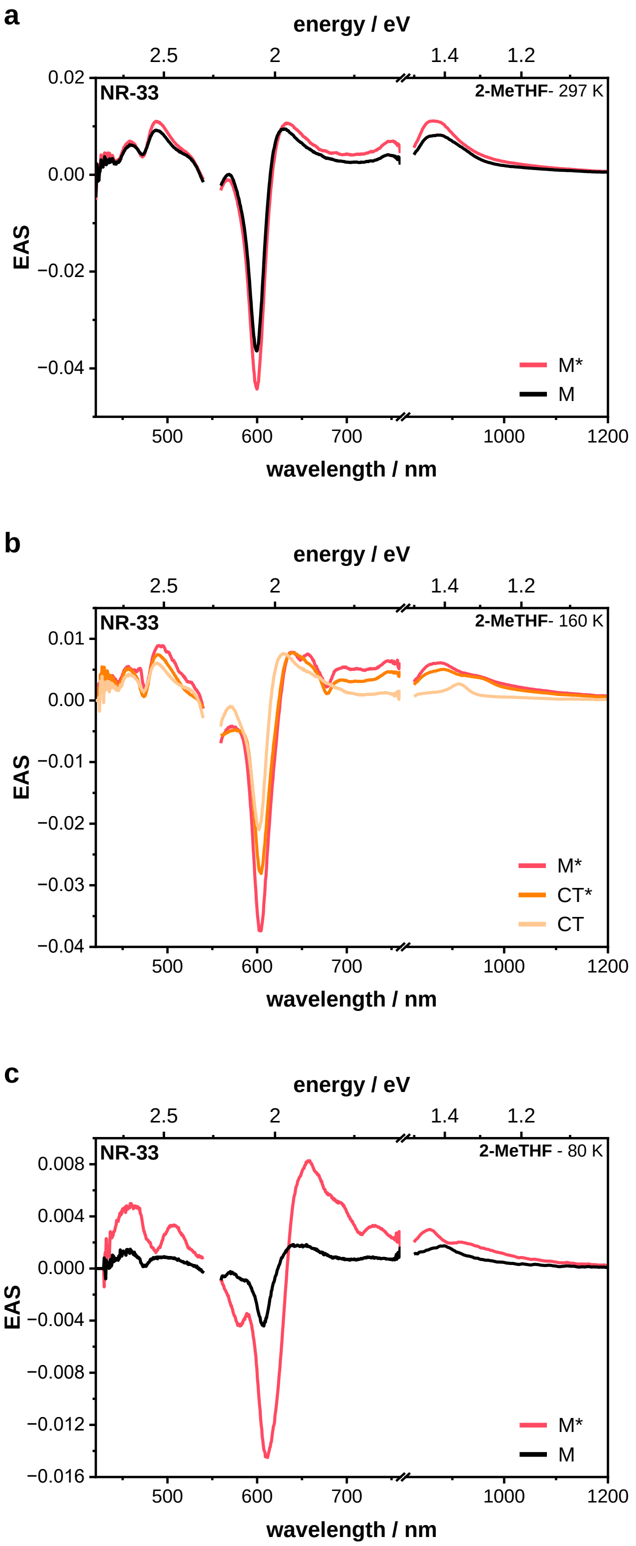

Figure S34 | Global analysis of the temperature-dependent femtosecond transient absorption data of NR-33. Deconvoluted evolution-associated spectra (EAS) of the mixed electronic state (M*, red), the subsequent solvent- and vibrationally-relaxed mixed electronic state (M; black), the pure (diabatic) intramolecular charge-transfer state (CT*; dark orange), and the structurally- and solvent-relaxed CT (light orange) as obtained by global analysis of the transient absorption data of **NR-33** in 2-MeTHF at (a) 297 K, (b) 160 K, and (c) 80 K shown in **Figure S27** using the kinetic model given in **Figure S31**.

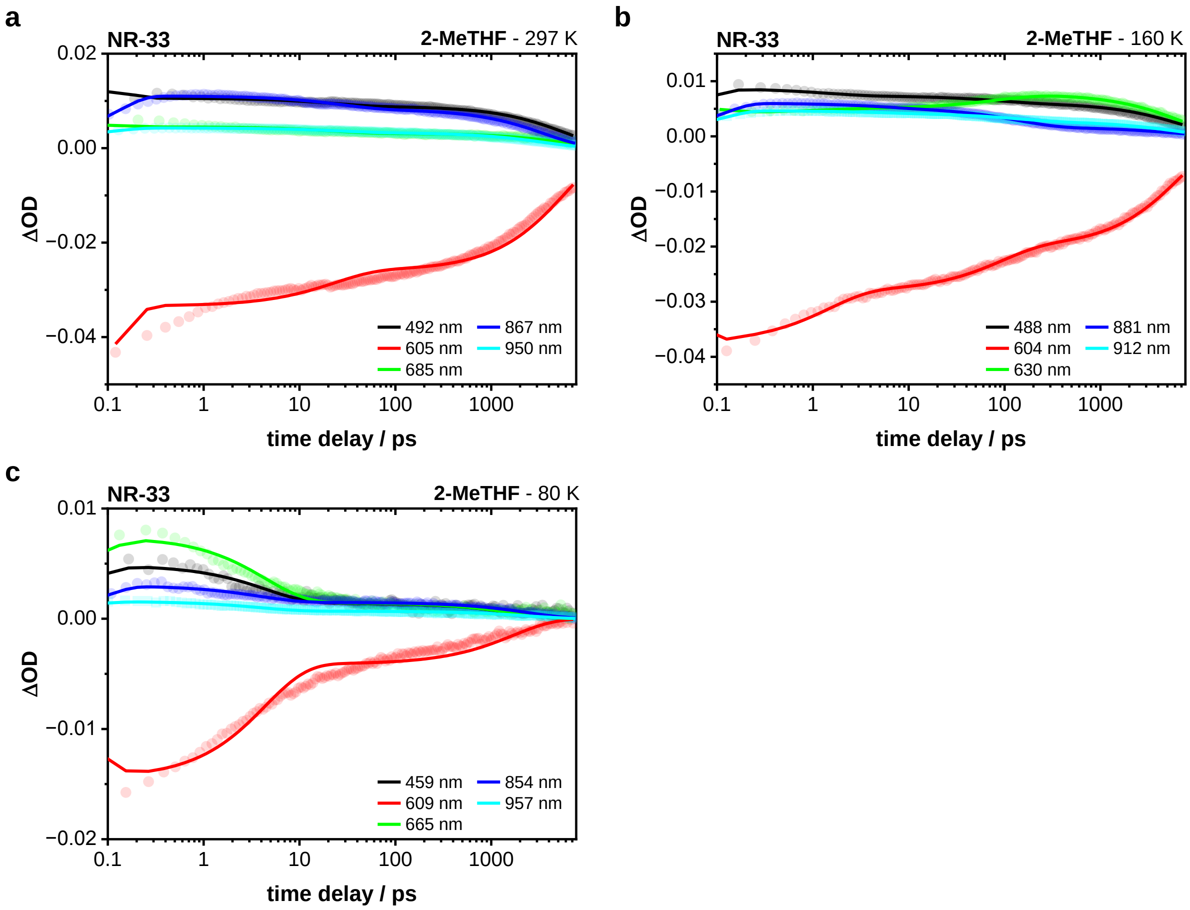

Figure S35 | Global analysis of the temperature-dependent femtosecond transient absorption data of NR-33. Raw data single-wavelength kinetics of selected wavelengths (scatter plot) and fits to the data (solid line) of **NR-33** in 2-MeTHF at (a) 297 K, (b) 160 K, and (c) 80 K shown in **Figure S27**. Raw data and corresponding fit for a particular wavelength are assigned to the same color (see figure legend for exact details).

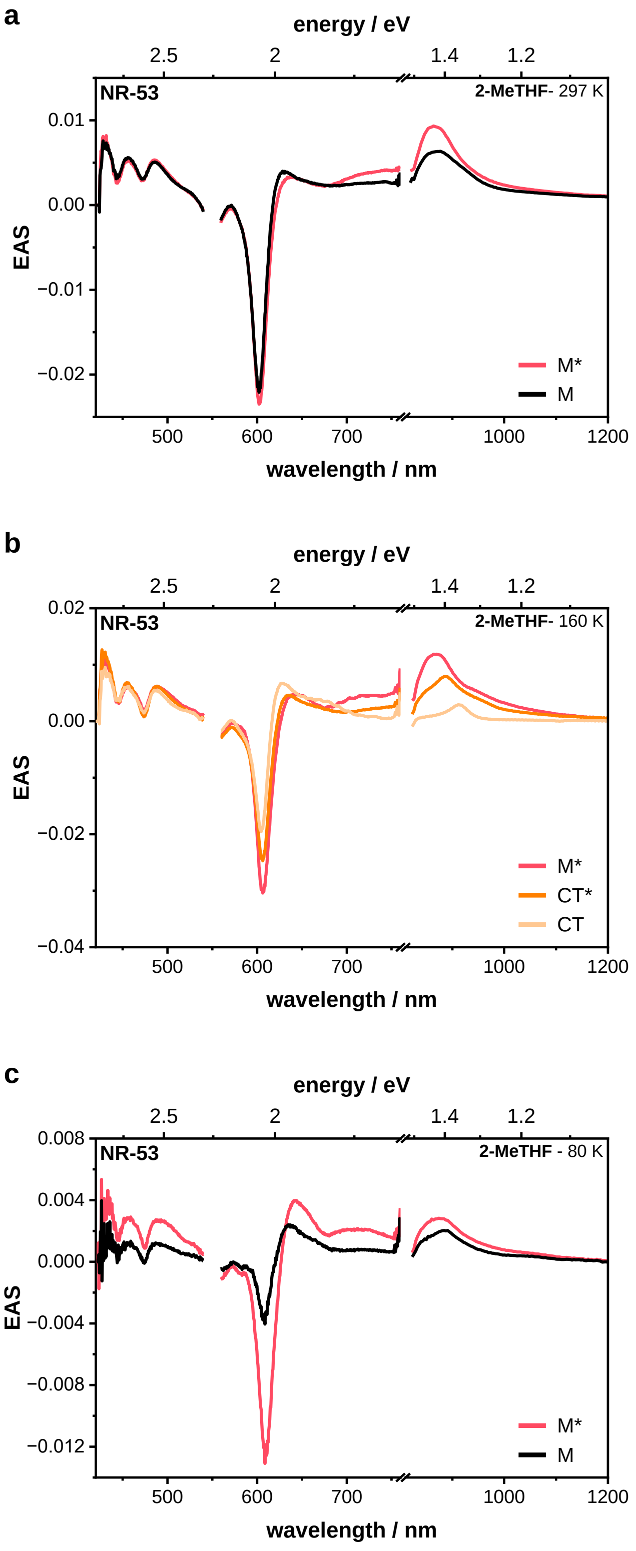

Figure S36 | Global analysis of the temperature-dependent femtosecond transient absorption data of NR-53. Deconvoluted evolution-associated spectra (EAS) of the mixed electronic state (M*; red), the subsequent solvent- and vibrationally-relaxed mixed electronic state (M; black), the pure (diabatic) intramolecular charge-transfer state (*i*-CT*; dark orange), and the structurally- and solvent-relaxed CT (light orange) as obtained by global analysis of the transient absorption data of **NR-53** in 2-MeTHF at (a) 297 K, (b) 160 K, and (c) 80 K shown in **Figure S29** using the kinetic model given in **Figure S31**.

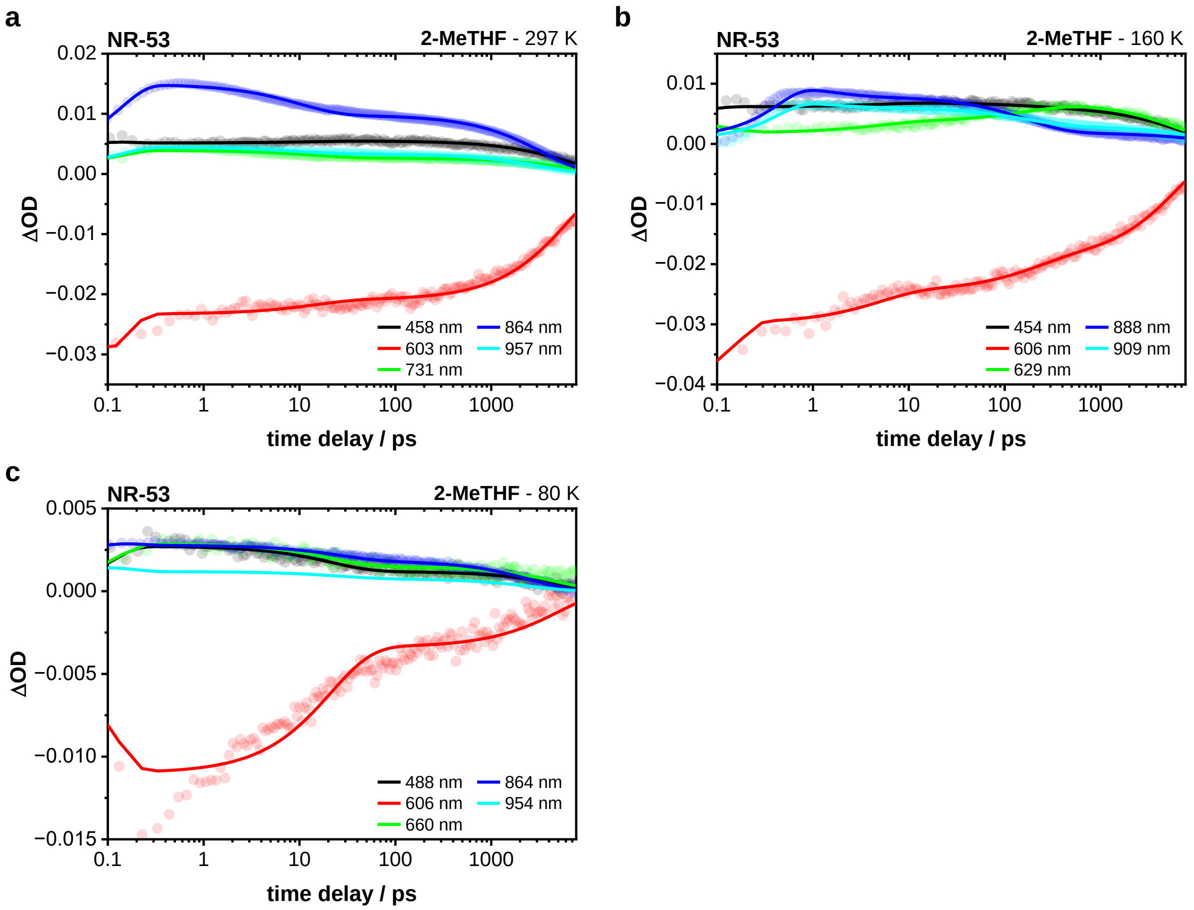

Figure S37 | Global analysis of the temperature-dependent femtosecond transient absorption data of NR-53. Raw data single-wavelength kinetics of selected wavelengths (scatter plot) and fits to the data (solid line) of **NR-53** in 2-MeTHF at (a) 297 K, (b) 160 K, and (c) 80 K shown in **Figure S29**. Raw data and corresponding fit for a particular wavelength are assigned to the same color (see figure legend for exact details).

Table S7 | Lifetimes obtained by global analysis of the femtosecond transient absorption data at cryogenic temperatures. Lifetimes of the hot mixed electronic state (M*), the subsequent solvent-and vibrationally-reorganized mixed electronic state (M), the pure (diabatic) intramolecular charge-transfer state (CT*), and the structurally- and solvent-relaxed CT as obtained by global analysis of the data of **NR-13**, **NR-33**, and **NR-53** recorded in 2-MeTHF upon 550 nm photoexcitation at different temperatures in femtosecond transient absorption spectroscopy experiments.

compound	temperature / K	lifetimes M* / ps	M / ns	CT* /ps	CT / ns
**NR-13**	**297**	16	6.6	-	-
	**160**	2.0	-	132	6.7[a]
	**80**	7.5	5.7	-	-
**NR-33**	**297**	25	4.8	-	-
	**160**	3.3	-	112	6.4[a]
	**80**	4.1	2.1	-	-
**NR-53**	**297**	13	5.0	-	-
	**160**	3.1	-	181	6.7[a]
	**80**	24	3.9	-	-

[a] The lifetime of the relaxed CT exceeds the achievable time delays of the instrument and, thus, should be interpreted with caution. Nonetheless, this lifetime is in good agreement with the lifetimes obtained from time-resolved emission measurements at 160 K (**Figures 2b**, **S11** and **S12**; **Table S6**).

2.2.3 Femtosecond transient infrared spectroscopy

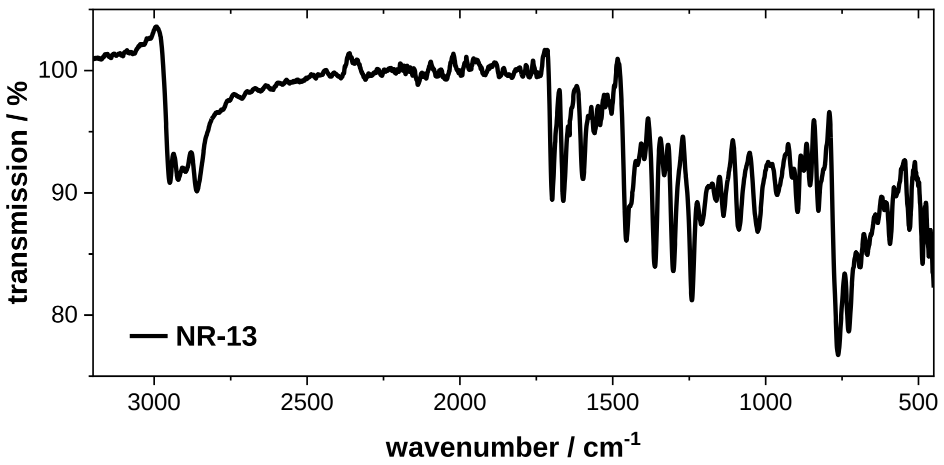

Figure S38 | Steady-state infrared transmission spectrum of NR-13. Attenuated total reflectance infrared transmission spectrum of **NR-13**.

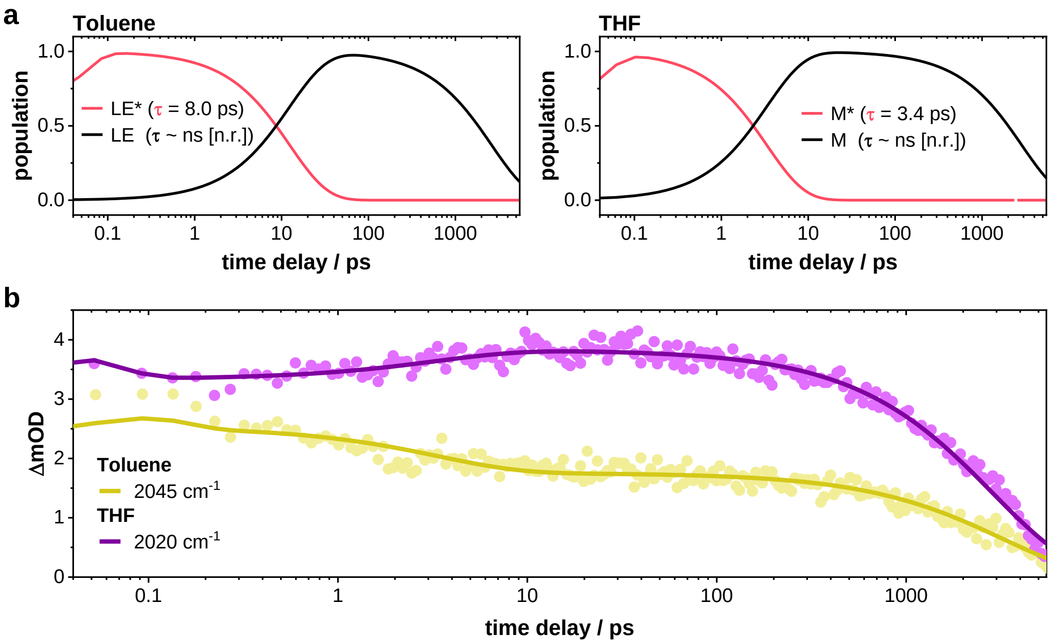

Figure S39 | Transient infrared data of NR-13 in solvents of different polarity. (a) Population dynamics in toluene and THF as obtained by global analysis of the transient IR data of **NR-13** shown in **Figure 4** using a two-species sequential model. (b) Raw data single-wavelength kinetics of selected wavelengths (scatter plot) and corresponding fits to the data (solid line) of **NR-13** in toluene (yellow) and THF (purple). Raw data and their respective fits are assigned to the same color.

2.3 Nanosecond transient absorption spectroscopy data

2.3.1 Nanosecond transient absorption spectroscopy experiments at room temperature

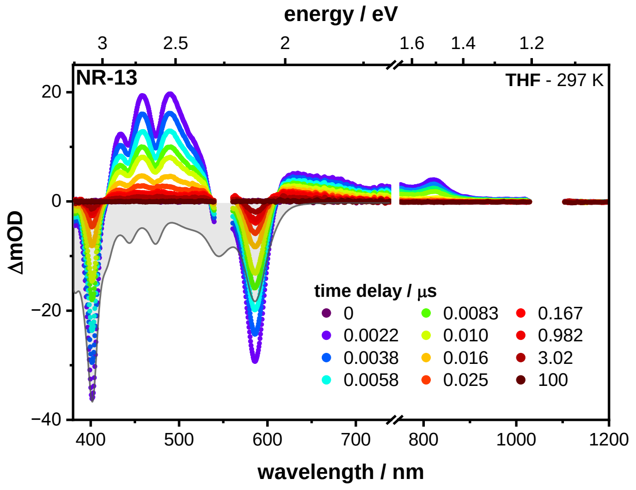

Figure S40 | Nanosecond transient absorption data of NR-13. Zero-point-corrected differential transient absorption (TA) spectra of **NR-13** obtained from nanosecond TA experiments upon photoexcitation at 550 nm (500 nJ) in argon-saturated THF at room temperature with various time delays between 0 to 100 µs (corresponding single-wavelength kinetics are shown in the supporting information in **Figure S49**).

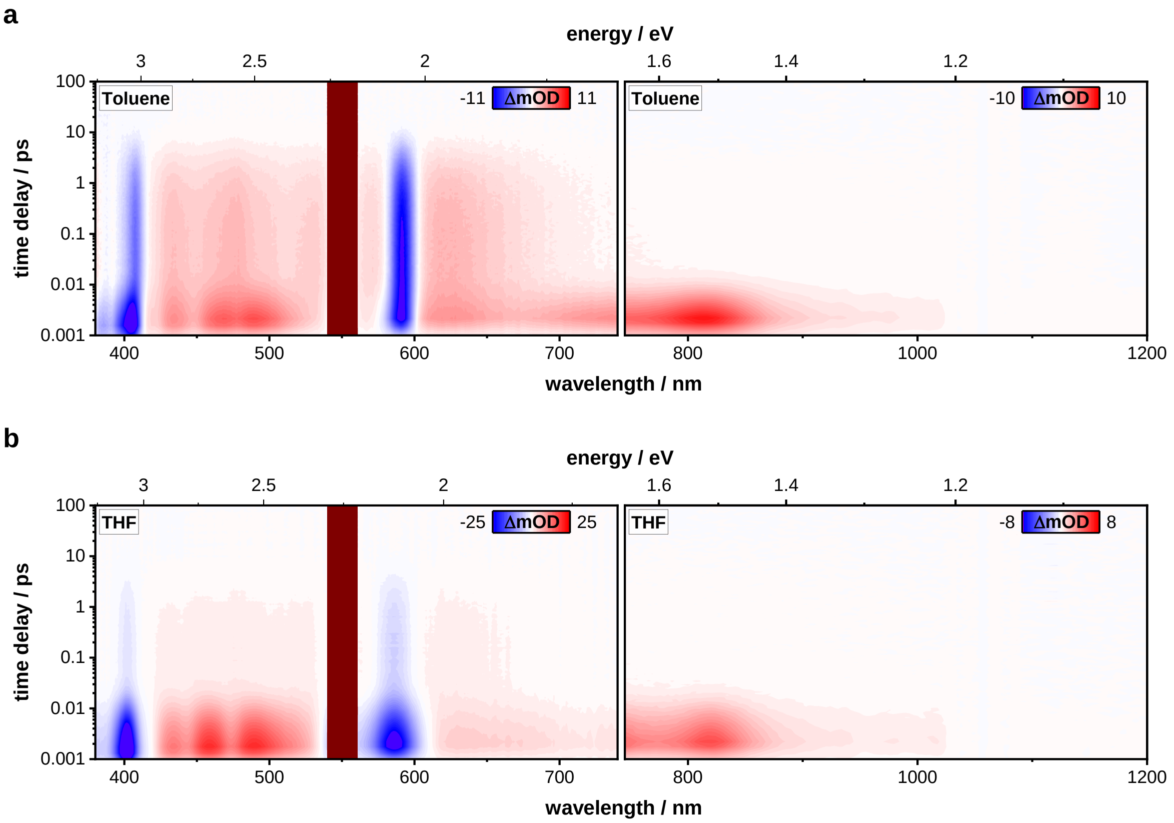

Figure S41 | Nanosecond transient absorption data of NR-13. Zero-point-corrected differential transient absorption (TA) 2D heat maps of **NR-13** obtained from nanosecond TA experiments upon photoexcitation at 550 nm (500 nJ) in argon-saturated (a) toluene and (b) THF at room temperature with various time delays between 0 to 100 μs (corresponding single-wavelength kinetics are shown in the supporting information in **Figure S49**).

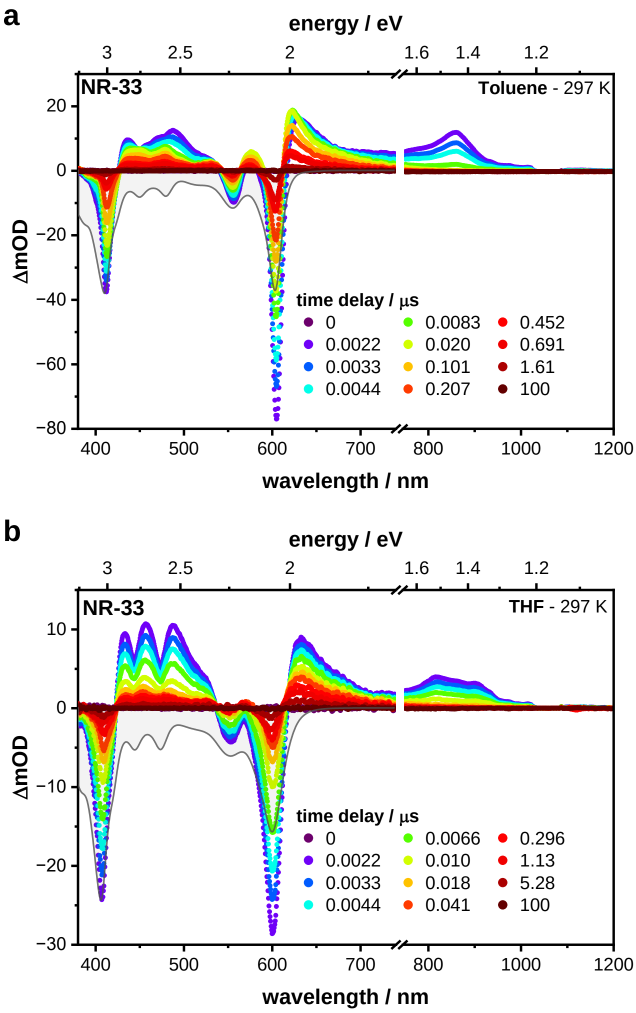

Figure S42 | Nanosecond transient absorption data of NR-33. Zero-point-corrected differential transient absorption (TA) spectra of **NR-33** obtained from nanosecond TA experiments upon photoexcitation at 550 nm (500 nJ) in argon-saturated (a) toluene and (b) THF at room temperature with various time delays between 0 to 100 µs (corresponding single-wavelength kinetics are shown in the supporting information in **Figure S51**).

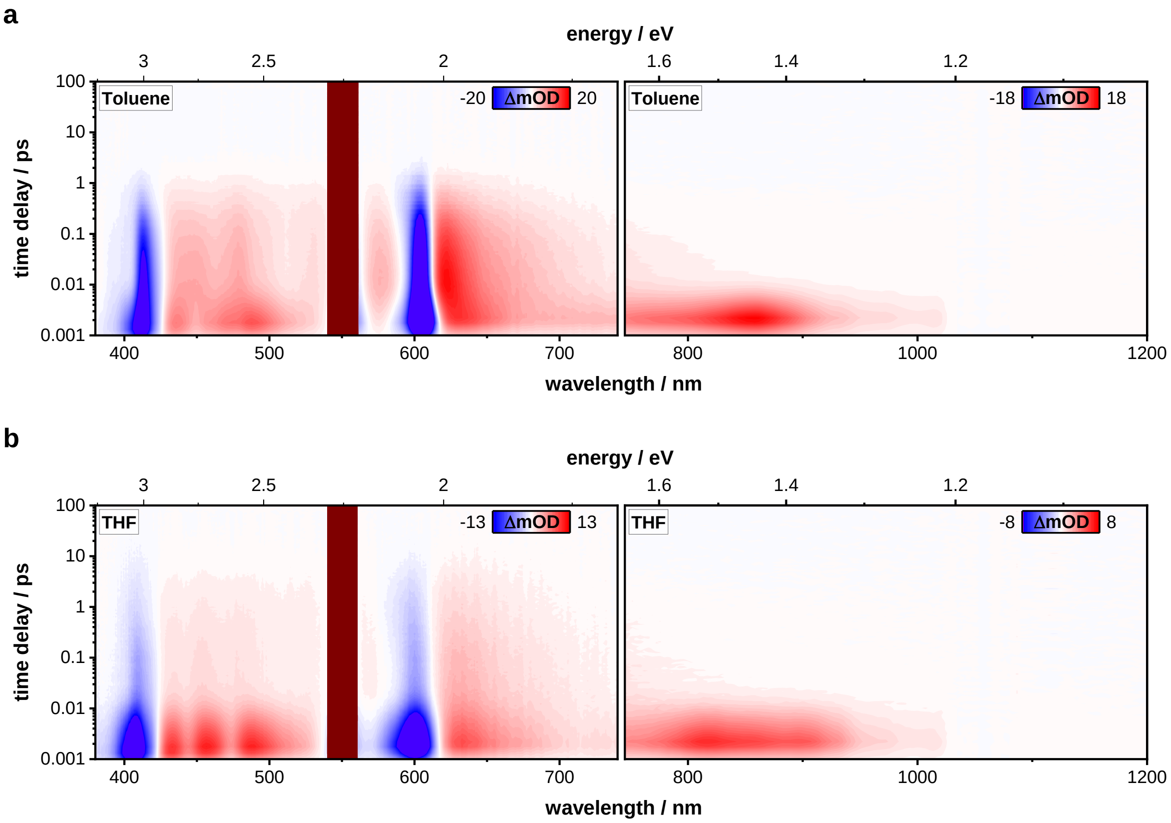

Figure S43 | Nanosecond transient absorption data of NR-33. Zero-point-corrected differential transient absorption (TA) 2D heat maps of **NR-33** obtained from nanosecond TA experiments upon photoexcitation at 550 nm (500 nJ) in argon-saturated (a) toluene and (b) THF at room temperature with various time delays between 0 to 100 μs (corresponding single-wavelength kinetics are shown in the supporting information in **Figure S51**).

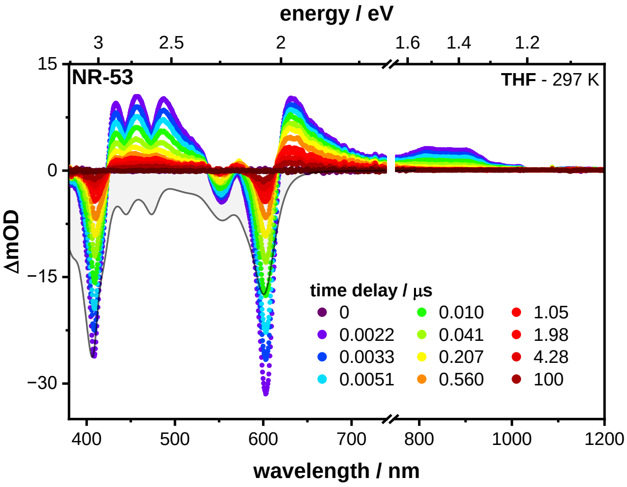

Figure S44 | Nanosecond transient absorption data of NR-53. Zero-point-corrected differential transient absorption (TA) spectra of **NR-53** obtained from nanosecond TA experiments upon photoexcitation at 550 nm (500 nJ) in argon-saturated THF at room temperature with various time delays between 0 to 100 µs (corresponding single-wavelength kinetics are shown in the supporting information in **Figure S54**).

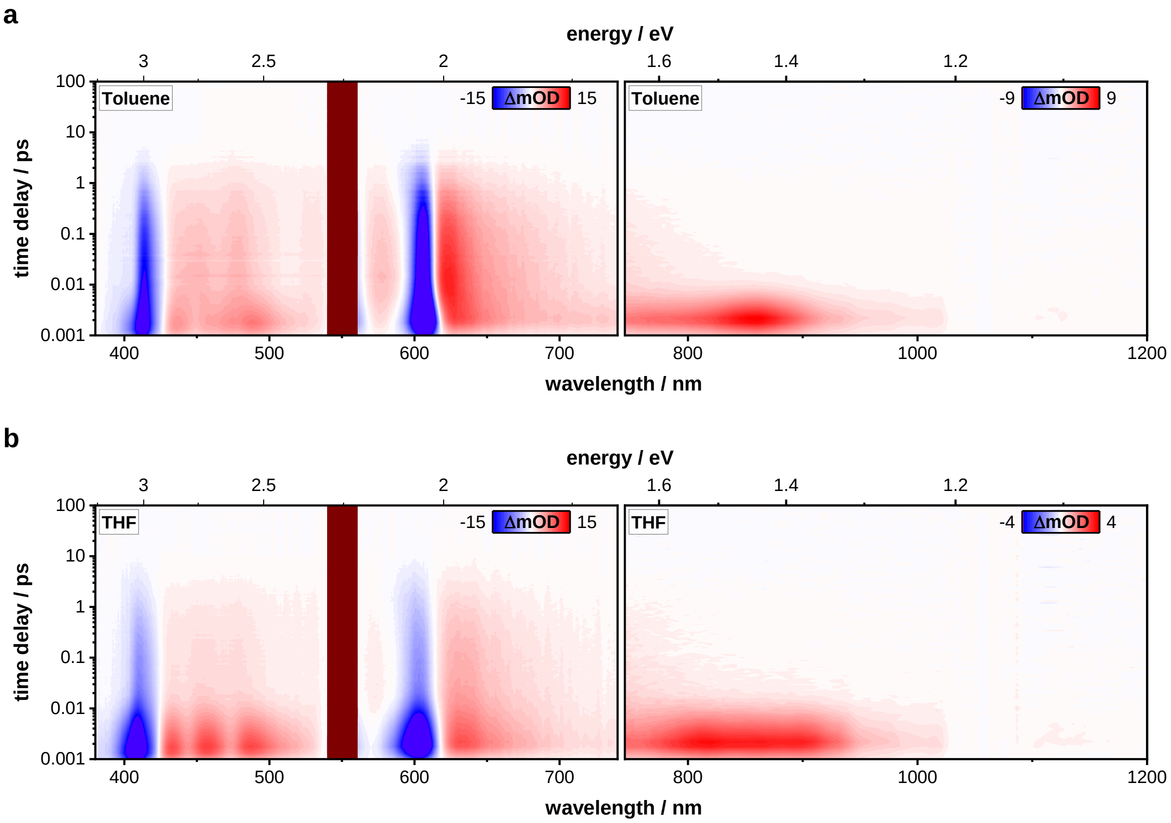

Figure S45 | Nanosecond transient absorption data of NR-53. Zero-point-corrected differential transient absorption (TA) 2D heat maps of **NR-53** obtained from nanosecond TA experiments upon photoexcitation at 550 nm (500 nJ) in argon-saturated (a) toluene and (b) THF at room temperature with various time delays between 0 to 100 µs (corresponding single-wavelength kinetics are shown in the supporting information in **Figure S54**).

model used to fit the nsTA data of **NR-13** in

Toluene and **THF**

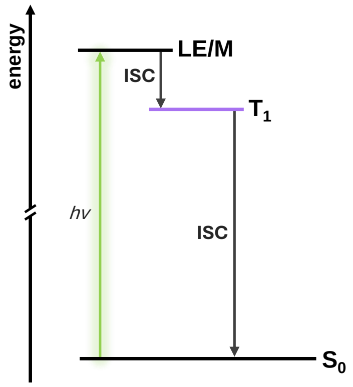

Figure S46 | Kinetic model employed to fit the nanosecond transient absorption data of NR-13 at room temperature. The deactivation cascade in toluene and THF is modeled using a two-species sequential model. Following initial photoexcitation into the mixed electronic state, which exhibits varying locally excited to charge-transfer character depending on solvent polarity (LE and M, respectively), **NR-13** undergoes intersystem crossing (ISC) to yield the first triplet excited state (T_1), after which the deactivation process is concluded.

model used to fit the nsTA data of **NR-33** and **NR-53** in

Toluene and **THF**

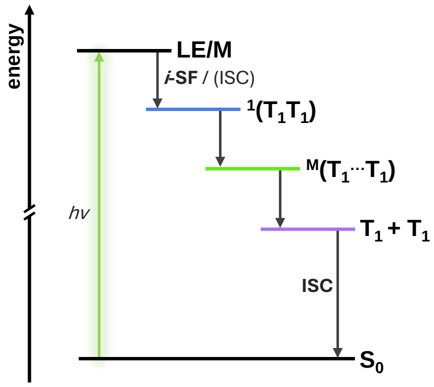

Figure S47 | Kinetic model employed to fit the nanosecond transient absorption data of NR-33 and NR-53 at room temperature. The deactivation cascade of **NR-33** and **NR-53** for both solvents is modeled using a four-species sequential model. Initial photoexcitation into the mixed electronic state, which exhibits varying locally excited to charge-transfer character depending on solvent polarity (LE and M, respectively), is followed by the formation of a singlet correlated triplet pair $^1(T_1T_1)$ *via* intramolecular singlet fission (*i*-SF). Subsequently, the weakly coupled triplet pair state $^M(T_1 \cdots T_1)$ is formed, before spin decoherence yields the free triplet excited state ($T_1 + T_1$). A parallel formation of T_1 with some minor contribution stemming from the intersystem crossing channel (ISC) cannot be precluded but is not modeled due to challenges differentiating between *via* ISC and *i*-SF generated triplets spectroscopically and, consequently, a lack of experimental data that would allow for an accurate modeling of such. Using a sequential model with three (or fewer) species results in prominent left and right singular vectors, indicating that the kinetic model is underrepresented. Specifically, the shape of the right singular vectors resembles a triplet excited state, suggesting that a sequential model with four species (modelling strongly and weakly coupled triplet pair intermediates) is the most appropriate.

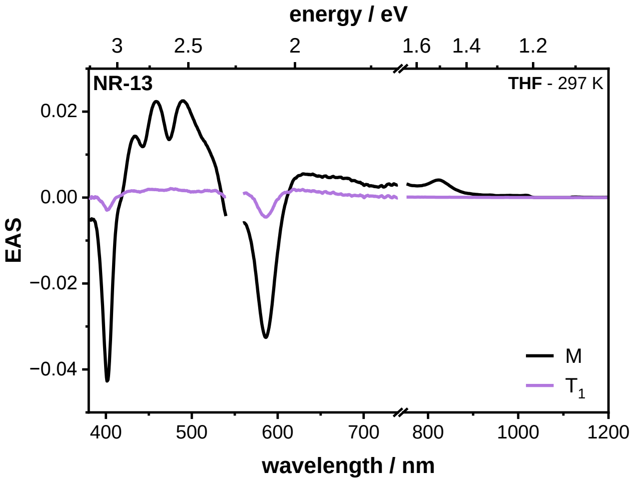

Figure S48 │ Global analysis of the nanosecond transient absorption data of NR-13. Deconvoluted evolution-associated spectra (EAS) of the mixed electronic state (M; black), and the triplet excited state (T_1; purple) as obtained by global analysis of the transient absorption data of **NR-13** in argon-saturated THF shown in **Figure S40** using the kinetic model given in **Figure S46**.

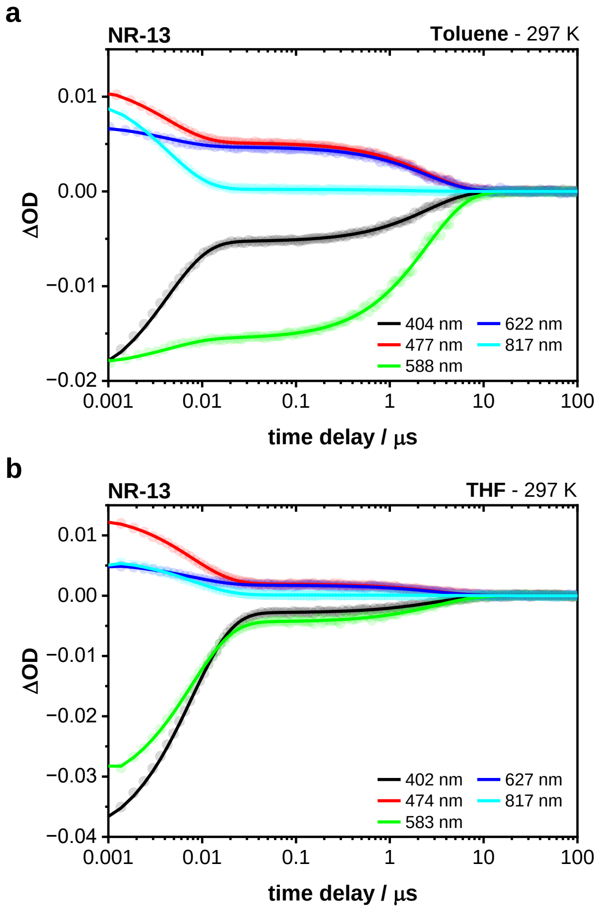

Figure S49 | Global analysis of the nanosecond transient absorption data of NR-13. Raw data single-wavelength kinetics of selected wavelengths (scatter plot) and fits to the data (solid line) of **NR-13** in (a) toluene and (b) THF at room temperature shown in **Figures 5** and **S40**. Raw data and corresponding fit for a particular wavelength are assigned to the same color (see figure legend for exact details).

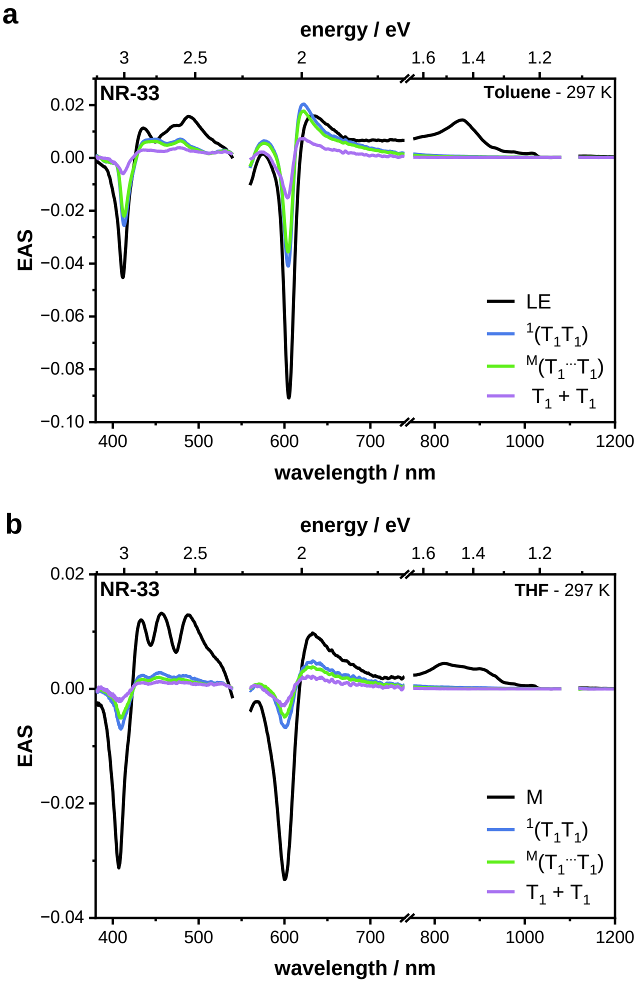

Figure S50 | Global analysis of the nanosecond transient absorption data of NR-33. Deconvoluted evolution-associated spectra (EAS) of the mixed electronic state (black), which has predominantly locally excited character (LE) or includes some charge-transfer contributions (M), the singlet correlated triplet pair state ($^1(T_1T_1)$; blue), the subsequent weakly coupled triplet pair state ($^M(T_1{\cdots}T_1)$; green), and the free triplet excited state ($T_1 + T_1$; purple) as obtained by global analysis of the transient absorption data of **NR-33** in argon-saturated (a) toluene and (b) THF shown in **Figure S42** using the kinetic model given in **Figure S47**.

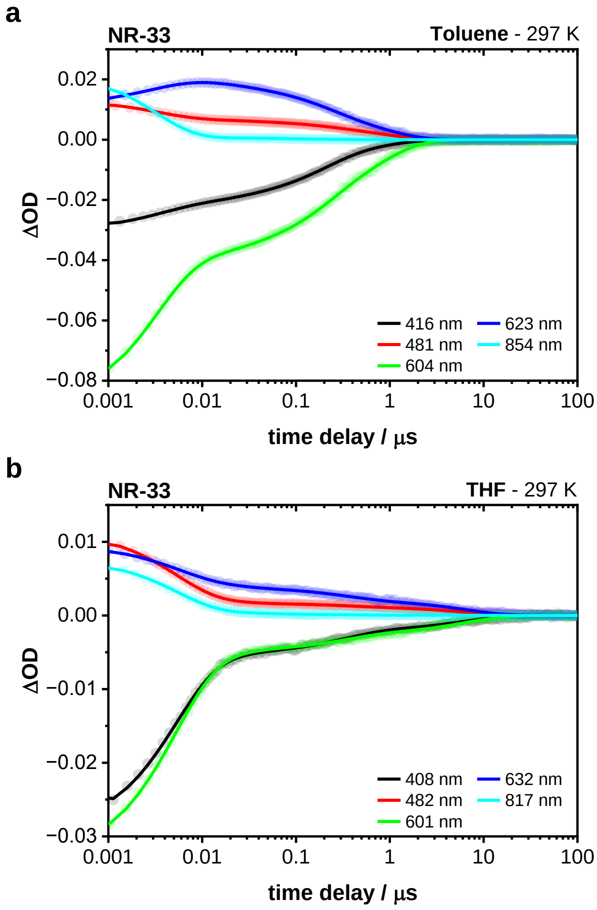

Figure S51 | Global analysis of the nanosecond transient absorption data of NR-33. Raw data single-wavelength kinetics of selected wavelengths (scatter plot) and fits to the data (solid line) of **NR-33** in (a) toluene, and (b) THF at room temperature shown in **Figure S42**. Raw data and corresponding fit for a particular wavelength are assigned to the same color (see figure legend for exact details).

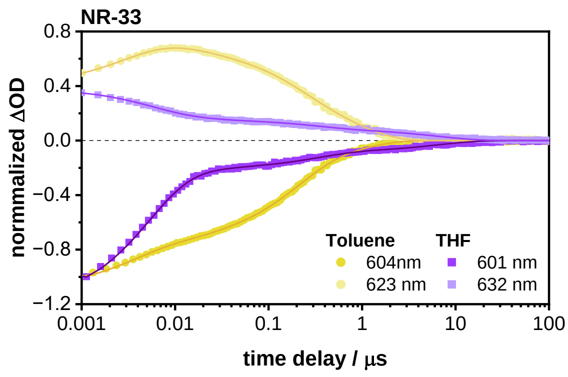

Figure S52 | Influence of solvent polarity on the triplet excited state formation for NR-33. Single-wavelength kinetics and corresponding fits of **NR-33** obtained from nanosecond transient absorption experiments shown in **Figure S42** highlighting the multiexponential decay as well as the reduced formation of long-lived species (namely, triplet excited states) in higher-polarity solvents.

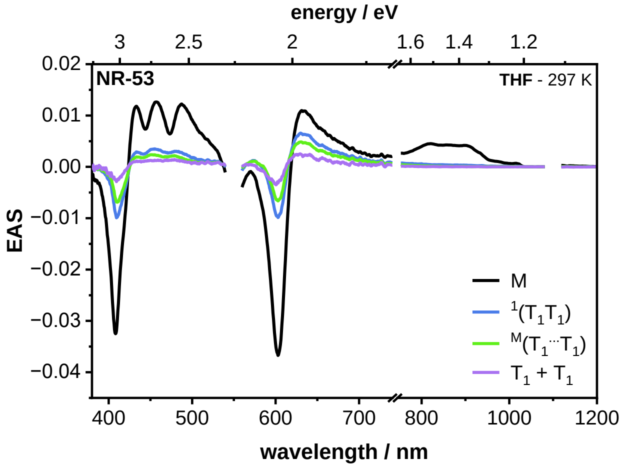

Figure S53 │ Global analysis of the nanosecond transient absorption data of NR-53. Deconvoluted evolution-associated spectra (EAS) of the mixed electronic state (M; black), the singlet correlated triplet pair state ($^1(T_1T_1)$; blue), the subsequent weakly coupled triplet pair state ($^M(T_1{\cdots}T_1)$; green), and the free triplet excited state ($T_1 + T_1$; purple) as obtained by global analysis of the transient absorption data of **NR-53** in argon-saturated THF shown in **Figure S44** using the kinetic model given in **Figure S47**.

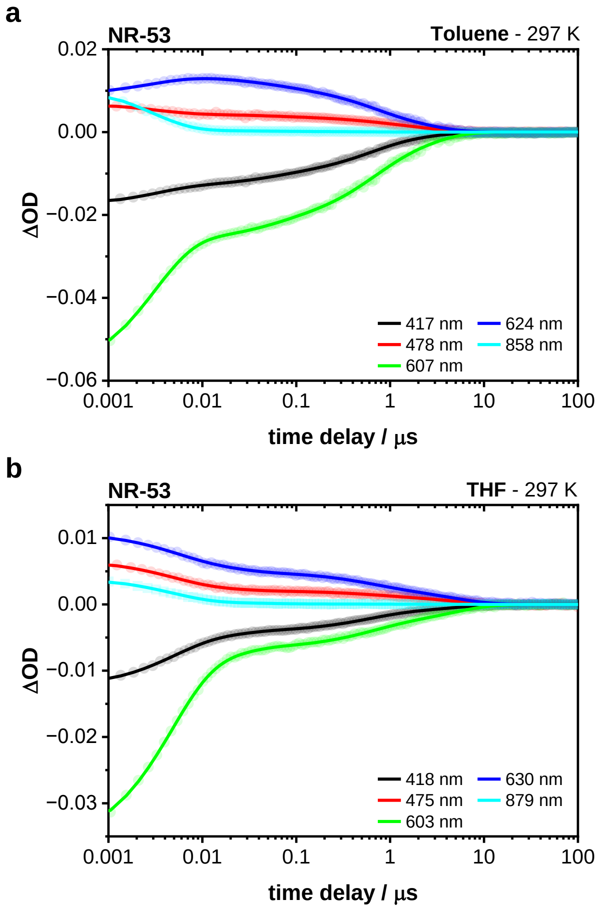

Figure S54 | Global analysis of the nanosecond transient absorption data of NR-53. Raw data single-wavelength kinetics of selected wavelengths (scatter plot) and fits to the data (solid line) of **NR-53** in (a) toluene and (b) THF at room temperature shown in **Figures 5** and **S44**. Raw data and corresponding fit for a particular wavelength are assigned to the same color (see figure legend for exact details).

Table S8 | Lifetimes obtained by global analysis of the femtosecond and nanosecond transient absorption data at room temperature. Lifetimes of the mixed electronic state, which exhibits varying locally excited to charge-transfer character depending on solvent polarity (LE* and M*, respectively), the solvent- and vibrationally-relaxed mixed electronic state (M), the singlet correlated triplet pair state $^1(T_1T_1)$, the weakly coupled triplet pair state $^M(T_1{\cdots}T_1)$, and the free triplet excited state (T_1 + T_1) obtained by global analysis of the data of **NR-13**, **NR-33**, and **NR-53** recorded in femtosecond transient absorption (fsTA) and nanosecond transient absorption (nsTA) experiments in toluene and THF, respectively.

		fsTA	nsTA			
**comp.**	**solvent**	**LE^*/M^* / ps**	**LE/M / ns**	**$^1(T_1T_1)$ / ns**	**$^M(T_1{\cdots}T_1)$ / ns**	**$T_1 + T_1$ / µs**
**NR-13**	**Toluene**	0.9	4.3	-	-	2.5
	**THF**	11	7.8	-	-	3.6
**NR-33**	**Toluene**	2.1	3.2	16	188	4.1
	**THF**	10	5.3	16	326	6.4
**NR-53**	**Toluene**	5	3.0	45	543	2.2
	**THF**	23	4.6	17	548	4.5

2.3.1.1 Triplet-sensitization experiments

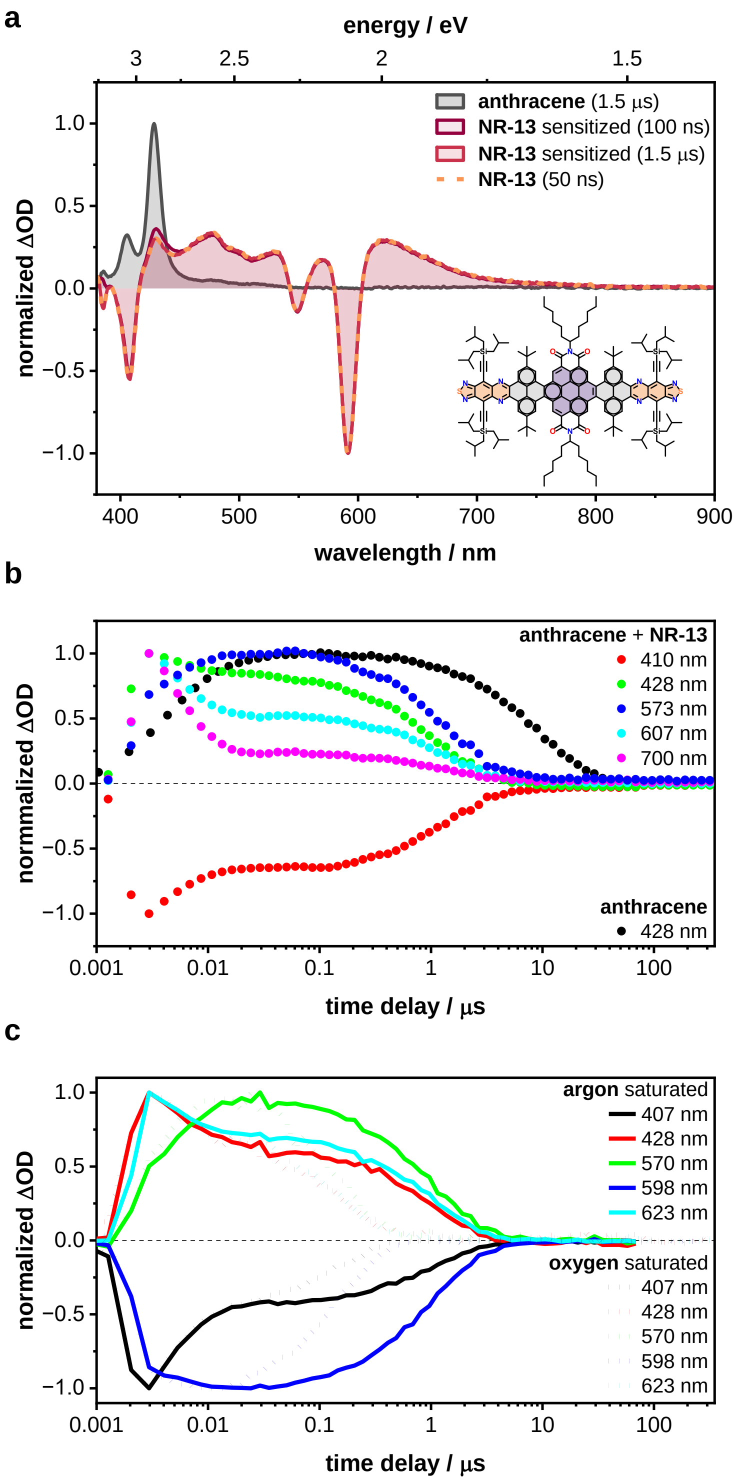

Figure S55 | Triplet-triplet sensitization. (a) Normalized zero-point-corrected differential absorption spectra of the triplet sensitizer anthracene (grey) and sensitized **NR-13** (orange/red) recorded in argon-saturated toluene at room temperature. (b) Single-wavelength kinetics of selected wavelengths of an anthracene/**NR-13** solution and pure anthracene (see figure legend for details). (c) Single-wavelength kinetics of selected wavelengths of **NR-13** in argon-saturated (solid line) as well as oxygen-saturated (dotted line) toluene to highlight the oxygen sensitivity of the observed long-lived species.

Conventional triplet-sensitization experiments using anthracene (or *N*-Methylfulleropyrrolidine) as triplet sensitizer were unsuccessful for the extended nanoribbons (**NR-33** and **NR-53**) due to their very large molar extinction coefficients ε. Such high ε, on one hand, limit the maximum concentration which can be used without absorbing all the white light at certain wavelengths. This is detrimental to quenching experiments though, as high quencher concentrations are desirable for efficient triplet-triplet energy transfer. On the other hand, large ε also render a selective excitation of the triplet sensitizer unlikely, further reducing the probability for a successful sensitization event. Therefore, the presence of long-lived triplet excited states was investigated by probing the sensitivity of the long-lived species to molecular oxygen in solution.

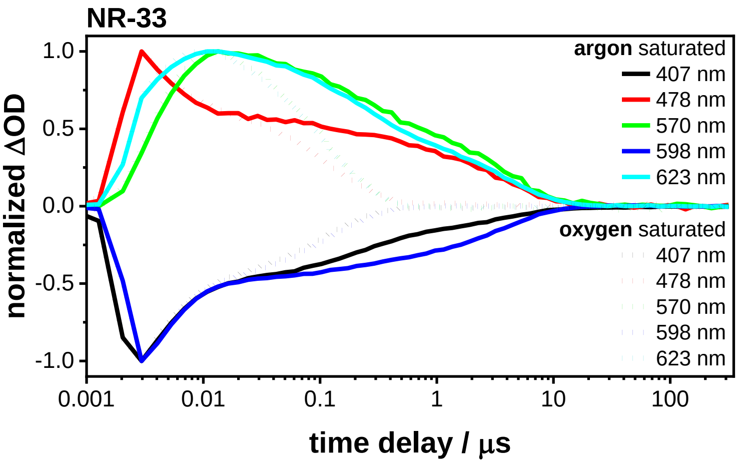

Figure S56 | Oxygen sensitivity of the long-lived species. Single-wavelength kinetics of selected wavelengths of **NR-33** in argon-saturated (solid line) as well as oxygen-saturated (dotted line) toluene to highlight the oxygen sensitivity of the observed long-lived species.

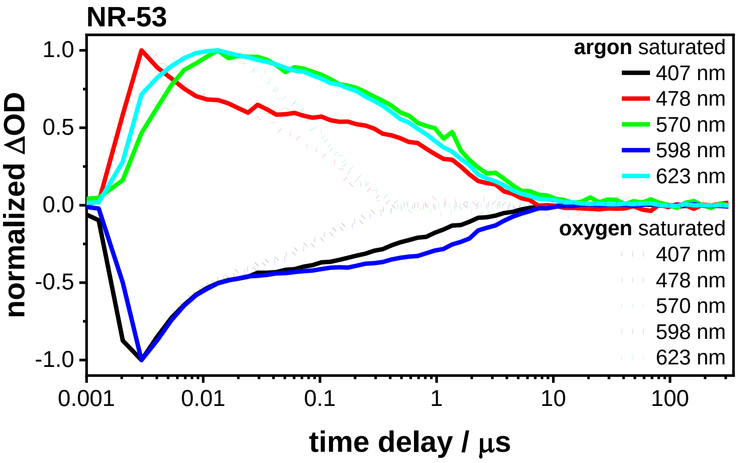

Figure S57 | Oxygen sensitivity of the long-lived species. Single-wavelength kinetics of selected wavelengths of **NR-53** in argon-saturated (solid line) as well as oxygen-saturated (dotted line) toluene to highlight the oxygen sensitivity of the observed long-lived species.

2.3.1.2 Triplet quantum yields

Due to overlapping excited-state absorption features of the triplet excited state with the mixed state as well as the ground-state bleaching, triplet quantum yields cannot be easily determined *via* the obtained EAS derived from global analysis. Instead, further experiments, i.e., emission of singlet oxygen in solution, were conducted in order to estimate the triplet quantum yields of the different nanoribbons, in general, and the singlet correlated triplet pair state $^1(T_1T_1)$ in the extended nanoribbons, in particular.

The analysis and calculation of the singlet oxygen yields (Φ_Δ) was conducted using the relative method.[5] Here, the integrated oxygen phosphorescence emission signal of a sample solution after O_2 purging for 20 min upon continuous monochromatic excitation at 385 nm is compared to the oxygen emission signal of a reference. In this study, C_{60} was used as a reference ($\Phi_{\Delta,C60}$ = 0.98 ± 0.05).[6] To minimize inaccuracies due to statistical errors in the absorption measurements, the integrated singlet oxygen emission of the reference and the nanoribbon samples was measured at various different optical densities at the excitation wavelength

Table S9 | Singlet oxygen yields. Determined singlet oxygen yields Φ_Δ of **NR-13**, **NR-33**, and **NR-53** in toluene using C_{60} as a reference.[5,6]

	NR-13	NR-33	NR-53
**Φ_Δ / %**	81	64	63

An error margin of ± 15 % is implicit in the determination of the singlet oxygen yields.

To calculate the initial $^1(T_1T_1)$ yield of **NR-33** and **NR-53**, the obtained singlet oxygen yields were used as baseline values for the extrapolation. It is assumed that a portion of the total triplet excited state population has already deactivated to the electronic ground state. This assumption is supported by lifetimes of $^1(T_1T_1)$ and the weakly interacting triplet pair $^M(T_1\cdots T_1)$, which range from tens to hundreds of nanoseconds. In contrast, the timescale for efficient collision and subsequent reaction of the nanoribbons with oxygen in solution is expected to be slow, on the order of hundreds of nanoseconds.

The slow physical quenching rates are attributed to the slow diffusion of molecular oxygen to the surface of the molecules, primarily due to the exceptionally large size of the nanoribbons, which acts as the rate-determining step. Estimating this diffusion using the Stokes-Einstein equation is challenging due to the non-spherical shape of the nanoribbons, which extend predominantly in one direction.

This reduces the efficiency of successful sensitization events between the extended nanoribbons (**NR-33** and **NR-53**) and oxygen. Given that the experiments were conducted in toluene—a nonpolar solvent with relatively low viscosity—an efficient quenching timescale of approximately 200 nanoseconds is assumed and used in the subsequent extrapolation.

The singlet correlated triplet pair yields can be extrapolated from the determined singlet oxygen yields (see **Table S9**) and the population dynamics as well as the dissociation yields (see **Table S10**) obtained from nanosecond transient absorption experiments as follows:

$^1(T_1T_1)$ yield of **NR-33**:

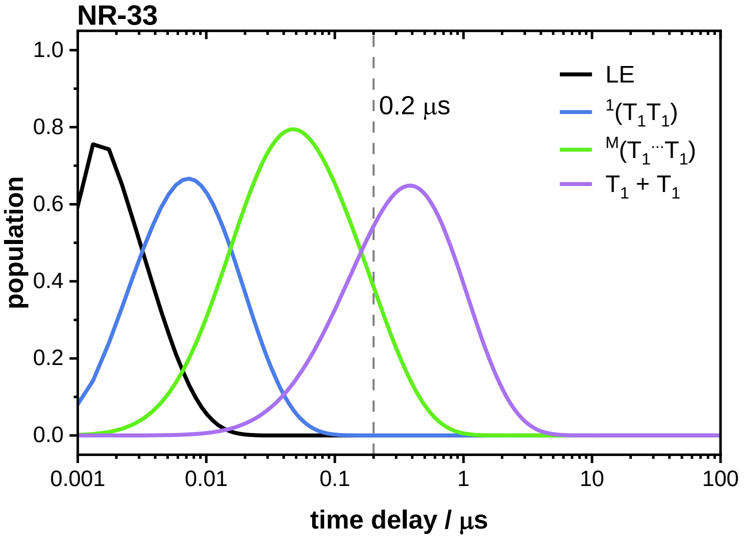

Figure S58 | Extrapolation of the $^1(T_1T_1)$ yield. Population dynamics of **NR-33** in argon-saturated toluene at room temperature obtained by global analysis of the nanosecond transient absorption data shown in **Figure S42**.

Φ_{1TT} = 64.2% (singlet oxygen yield) × [{0.0 (population of $^1(T_1T_1)$ at t = 200 ns)} + {0.39 (population of $^M(T_1\cdots T_1)$ at t = 200 ns) / 0.87 (dissociation yield going from $^1(T_1T_1)$ to $^M(T_1\cdots T_1)$)} + {0.54 (population of $T_1 + T_1$ at t = 200 ns) / 0.43 (dissociation yield going from $^M(T_1\cdots T_1)$ to $T_1 + T_1$ / 0.87 (dissociation yield going from $^1(T_1T_1)$ to $^M(T_1\cdots T_1)$))}] = 122%

$^1(T_1T_1)$ yield of **NR-53**:

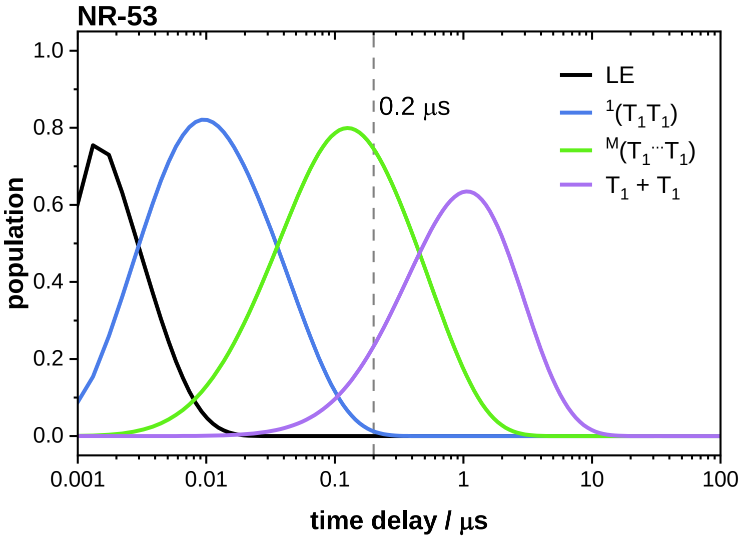

Figure S59 | Extrapolation of the $^1(T_1T_1)$ yield. Population dynamics of **NR-53** in argon-saturated toluene at room temperature obtained by global analysis of the nanosecond transient absorption data shown in **Figure 5**.

Φ_{1TT} = 63.5% × (0.013 + [0.74 / 0.81] + [{0.23 / 0.81} / 0.35]) = 111%

Table S10 | Extrapolated singlet correlated triplet pair yields. Calculated singlet correlated triplet pair $^1(T_1T_1)$ yields (Φ_{1TT}) of **NR-33** and **NR-53** in argon-saturated toluene at room temperature extrapolated based on the determined singlet oxygen yields (see **Table S9**) and the population dynamics derived by global analysis (see **Figures S58** and **S59**).

	**NR-33**	**NR-53**
**population** $^1(T_1T_1)$ at **200 ns**	0.00	0.013
**population** $^M(T_1 \cdots T_1)$ at **200 ns**	0.39	0.74
**population** T_1 at **200 ns**	0.54	0.23
**dissociation yield** $^1(T_1T_1) \rightarrow {}^M(T_1 \cdots T_1)$	0.87	0.81
**dissociation yield** $^M(T_1 \cdots T_1) \rightarrow T_1 + T_1$	0.43	0.35
Φ_{1TT} **/ %**	122	111

An error margin of ± 20% is implicit in the determination of the $^1(T_1T_1)$ yields due to the various assumptions made in the calculation.

2.3.2 Nanosecond transient absorption spectroscopy experiments at cryogenic temperatures

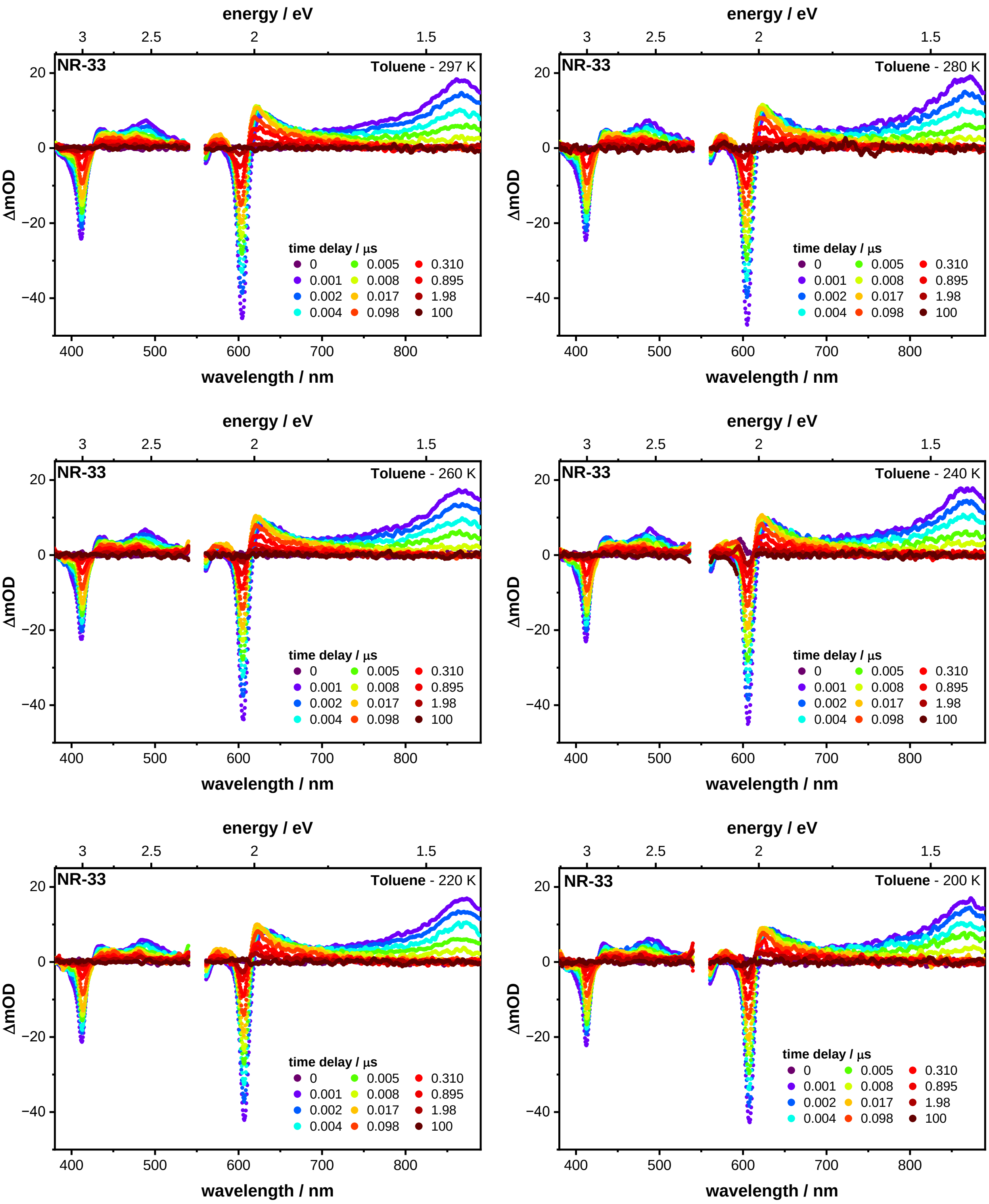

Figure S60 | Temperature-dependent nanosecond transient absorption data of NR-33. Zero-point-corrected differential transient absorption (TA) spectra of **NR-33** obtained from nanosecond TA experiments upon photoexcitation at 550 nm (500 nJ) in argon-saturated toluene at different temperatures between 297 and 200 K (see figure legend for exact values) with various time delays between 0 to 100 µs (corresponding single-wavelength kinetics are shown in the supporting information in **Figures S62**).

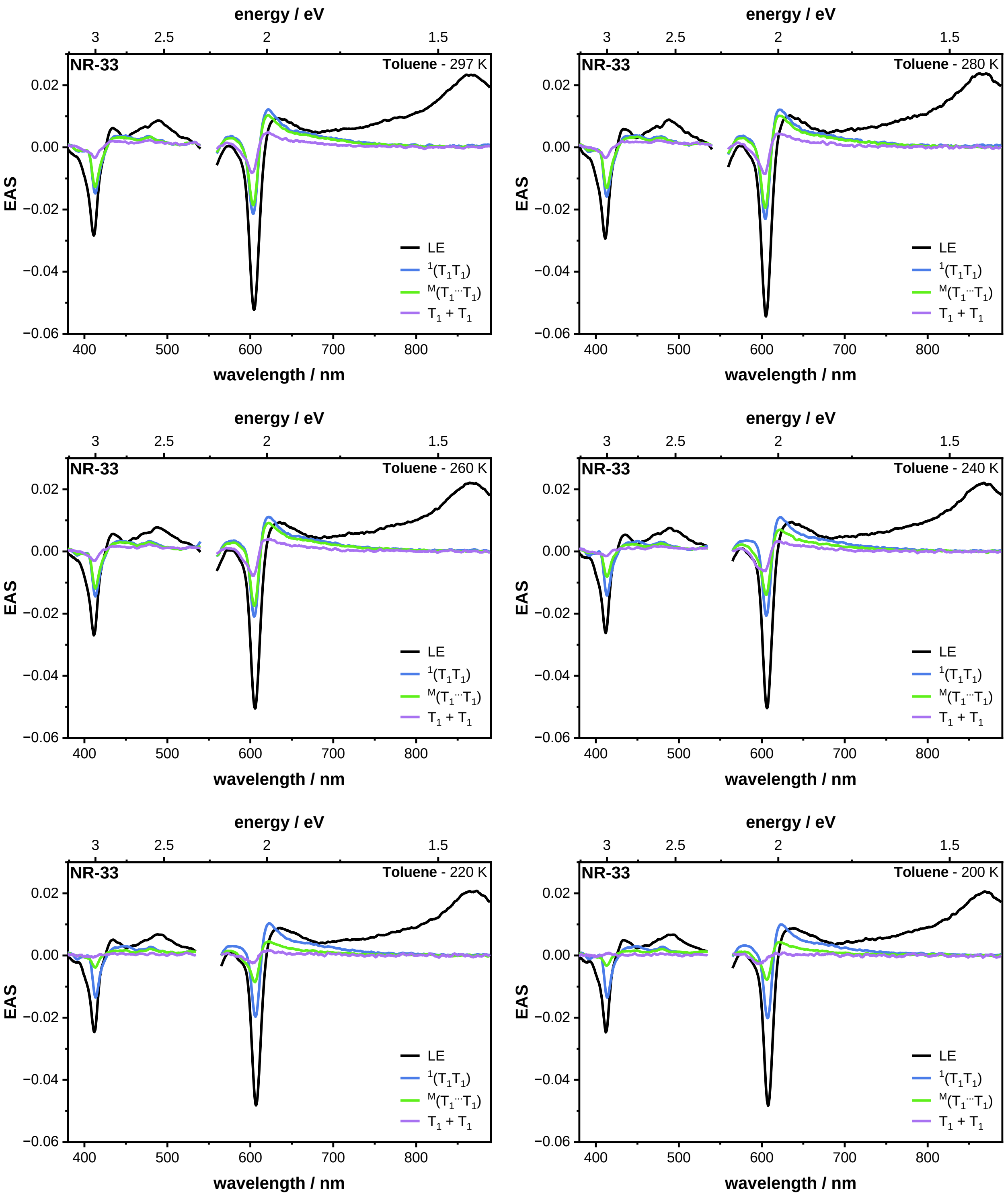

Figure S61 | Global analysis of the temperature-dependent nanosecond transient absorption data of NR-33. Deconvoluted evolution-associated spectra (EAS) of the initially populated electronic state with predominant locally excited character (LE; black), the singlet correlated triplet pair state ($^1(T_1T_1)$; blue), the subsequent weakly coupled triplet pair state ($^M(T_1\cdots T_1)$; green), and the free triplet excited state ($T_1 + T_1$; purple) as obtained by global analysis of the transient absorption data of **NR-33** in argon-saturated toluene at different temperatures between 297 and 200 K (see figure legend for exact values) shown in **Figure S60** using the kinetic model given in **Figure S47**.

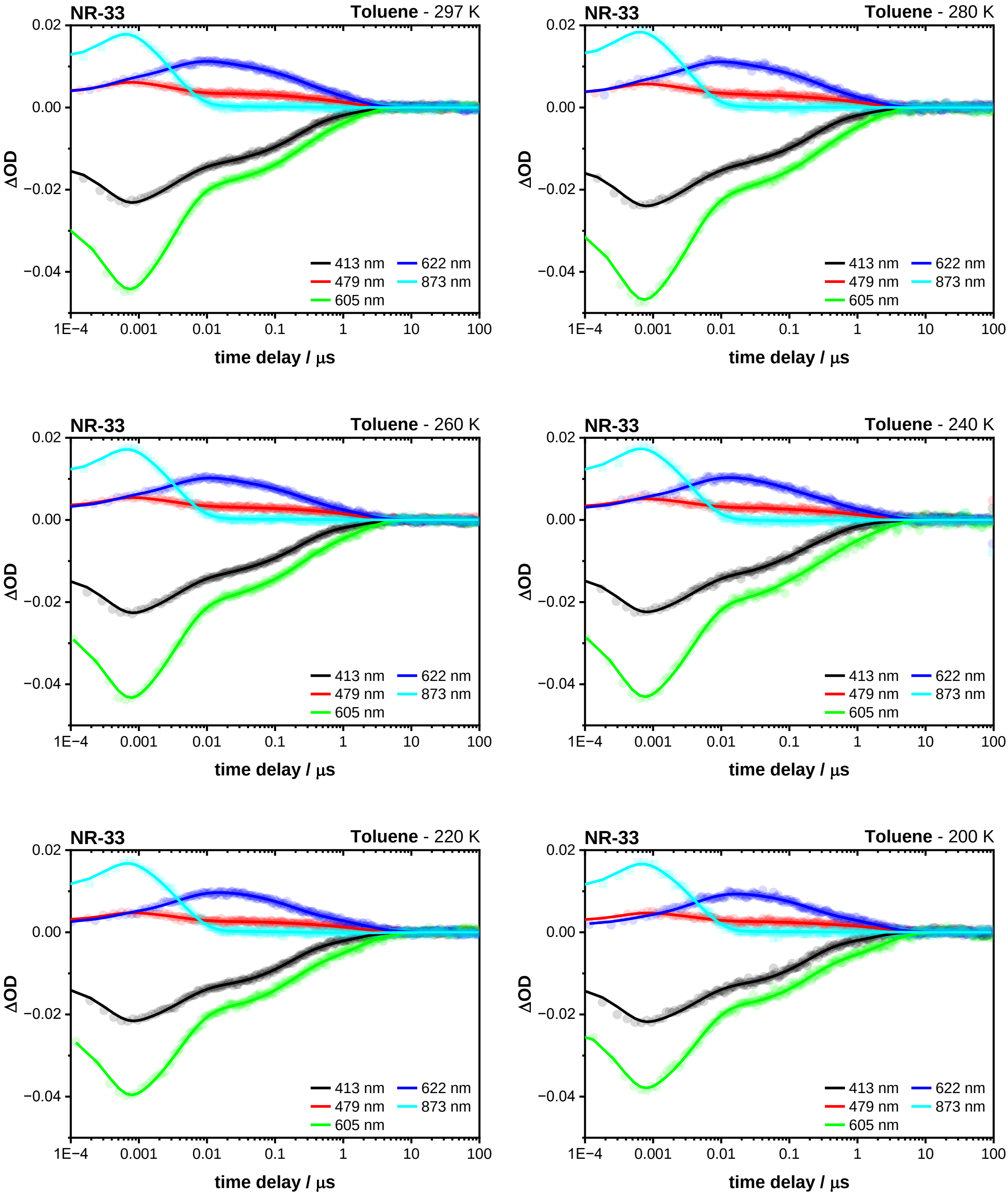

Figure S62 | Global analysis of the temperature-dependent nanosecond transient absorption data of NR-33. Raw data single-wavelength kinetics of selected wavelengths (scatter plot) and fits to the data (solid line) of **NR-33** in toluene at different temperatures between 297 and 200 K shown in **Figure S60**. Raw data and corresponding fit for a particular wavelength are assigned to the same color (see figure legend for exact details).

Table S11 | Lifetimes and triplet (dissociation) yields of NR-33 obtained by global analysis of the nanosecond transient absorption data at cryogenic temperatures. Lifetimes and yields of the solvent- and vibrationally-relaxed mixed electronic state with predominant locally excited character (LE), the singlet correlated triplet pair state $^1(T_1T_1)$, the weakly coupled triplet pair state $^M(T_1 \cdots T_1)$, and the free triplet excited state ($T_1 + T_1$) obtained by global analysis of the data of **NR-33** recorded in argon-saturated toluene upon 550 nm excitation in nanosecond transient absorption experiments.

	lifetimes				yields		
**temperature / K**	**LE / ns**	**$^1(T_1T_1)$ / ns**	**$^M(T_1 \cdots T_1)$ / ns**	**$T_1 + T_1$ / µs**	**$^1(T_1T_1)$ / %**	**$^M(T_1 \cdots T_1)$ / %**	**$T_1 + T_1$ / %**
**297 K**	3.1	15	202	1.3	122	84.7	41.7
**280 K**	3.2	16	212	1.4	118[a]	82.7	40.9
**260 K**	3.4	21	213	1.6	120[a]	82.5	38.6
**240 K**	3.7	71	346	1.9	121[a]	62.6	37.8
**220 K**	3.9	156	1133	2.8	120[a]	41.2	34.1
**200 K**	4.1	176	1840	2.9	115[a]	35.8	24.1

[a] $^1(T_1T_1)$ yields (at temperatures < 297 K) are calculated by comparing the ratio of $^1(T_1T_1)$ to LE at the respective temperatures to the ratio obtained at room temperature (for which the yield of $^1(T_1T_1)$ was extrapolated to 122%).

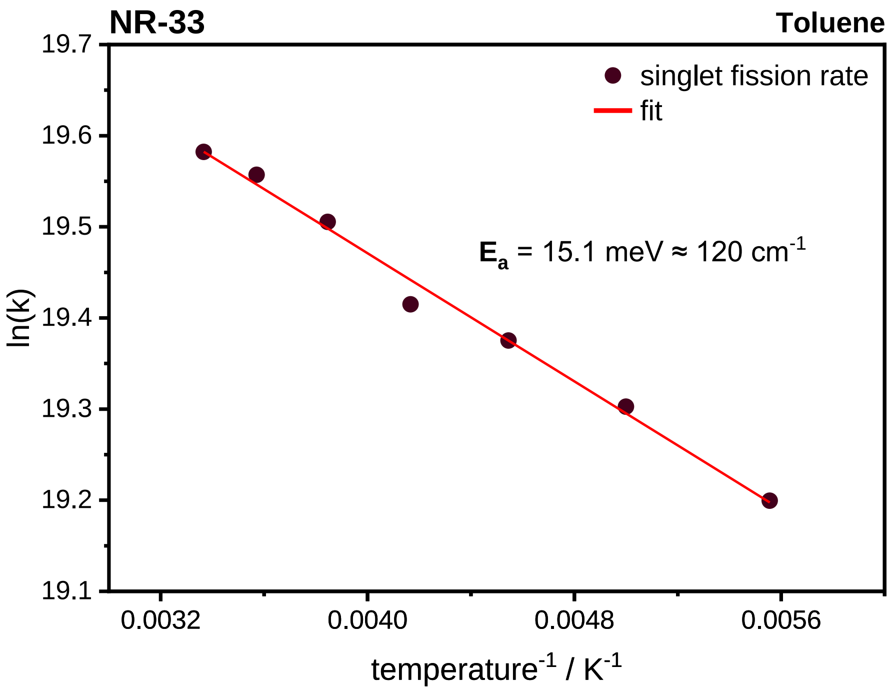

Figure S63 | Arrhenius plot of the singlet fission rate . Determination of the activation energy of the first step of singlet fission, that is, the formation of $^1(T_1T_1)$, by plotting the logarithm of the singlet fission rate against the reciprocal of the temperature in a so-called Arrhenius plot. The red line corresponds to the best linear fit of the data (black scatter plot) yielding a slope of $-\frac{E_a}{R}$ = 175.8 K for **NR-33** in argon-saturated toluene (R^2 = 0.99).

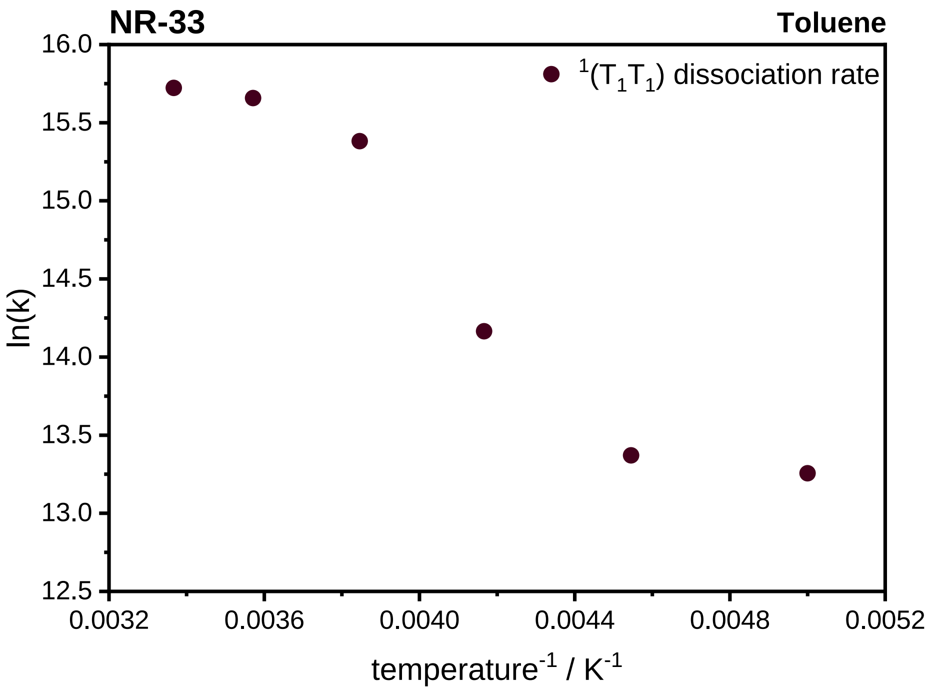

Figure S64 | Logarithm of the triplet dissociation rate from $^1(T_1T_1)$ to $^M(T_1{\cdots}T_1)$ as a function of the reciprocal temperature. Plot of the logarithm of the triplet dissociation rate, that is, the decoherence of the singlet correlated triplet pair state $^1(T_1T_1)$ into the weakly coupled triplet pair state $^M(T_1{\cdots}T_1)$, as a function of the reciprocal of the temperature.

Figure S64 demonstrates that the triplet dissociation rate from $^1(T_1T_1)$ to $^M(T_1{\cdots}T_1)$ decreases with decreasing temperature, but the relationship is not linear; instead, it appears to be a complex, multifaceted process. As indicated by the lifetimes of $^M(T_1{\cdots}T_1)$ approaching those of the free triplets T_1 at lower temperatures (**Table S11**), and considering a potential minor, parallel contribution *via* intersystem crossing to the formation of long-lived triplet species, providing an exact explanation for the observed dynamics proves challenging. Nonetheless, this data suggests that the generation of $^M(T_1{\cdots}T_1)$ involves a thermally-activated process likely requiring some degree of flexibility and associated torsional motion within the nanoribbons. Such intramolecular torsional motion modulates intertriplet exchange couplings, which is crucial for spin evolution from $^1(T_1T_1)$ to $^5(T_1T_1)$, or $^M(T_1{\cdots}T_1)$ in this context.

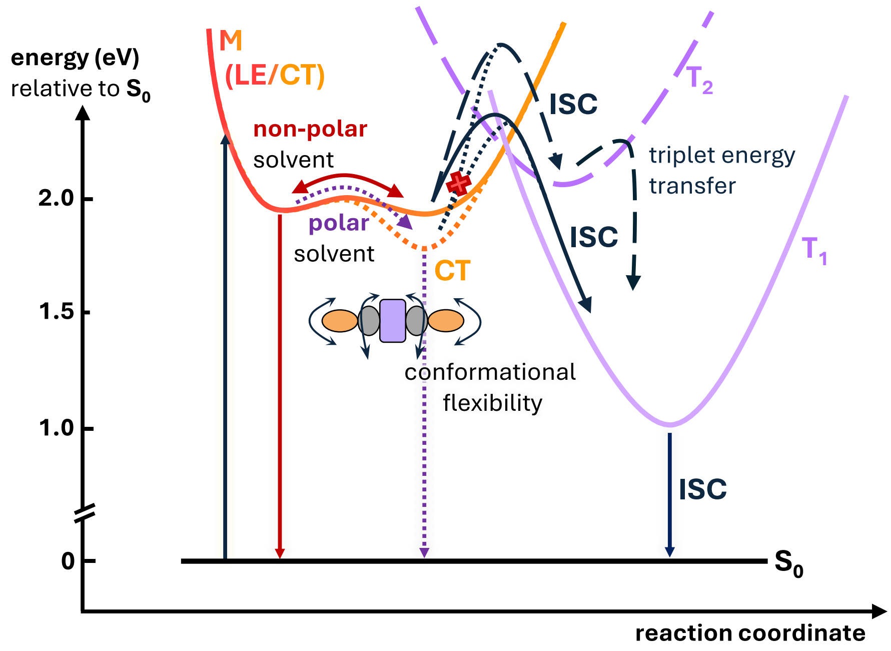

Figure S65 | Deactivation cascade of nanoribbon NR-13. Following the lowest-energy optical transition, the system transitions into a state exhibiting an admixture of locally excited (LE) and charge-transfer (CT) character owing to its push-pull nature. The environment governs the ratio of LE to CT and, thereby, influences the subsequent deactivation cascade. In non-polar solvents, **NR-13** shows very efficient triplet formation as well. However, unlike **NR-33** and **NR-53**, triplets are not formed *via* intramolecular singlet fission but intersystem crossing (presumably due to the incorporation of sulfur atoms in the terminal (acceptor) pyrazino-benzothiadiazole subunits). In more polar solvents, the stabilization of the CT state shifts the dynamics towards the formation of a diabatic CT state, whose formation is accompanied by significant structural reorganization, similar to **NR-33** and **NR-53** (see **Figure 6**).

3. Spectro-electrochemistry

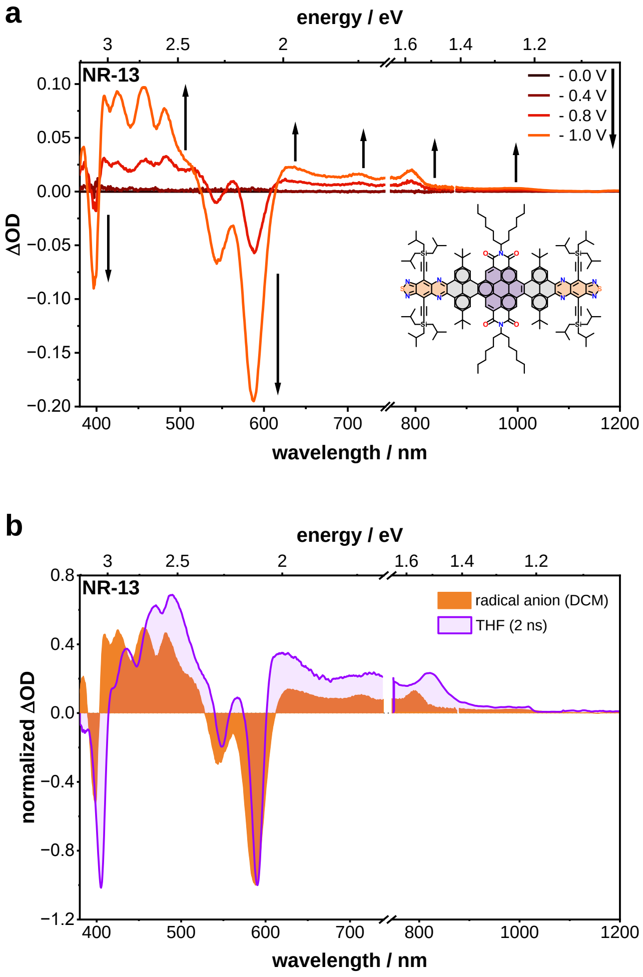

Figure S66 | Spectro-electrochemistry. (a) Differential absorption spectra (ΔOD) of the radical anion of **NR-13** derived from spectro-electrochemistry measurements in dichloromethane (DCM) under a negative potential versus an Ag quasi-reference electrode (see legend). (b) Zero-point-corrected differential absorption spectra of **NR-13** in THF (purple) recorded at a time delay of 2 ns in comparison to the differential absorption spectrum of the anion of **NR-13** derived from spectro-electrochemistry measurements shown in (a) to demonstrate the qualitative similarities in their shape underscoring the pronounced charge-transfer character in the mixed excited state.

4. Computational analysis

Determination of the Onsager radii

First the structures were preoptimized using the AM1* method as implemented in the Empire software package.[7–12] Following that, the preoptimized structures were further geometrically optimized employing density functional theory (DFT), more precisely the B3LYP correlation-exchange functional and Pople´s 6-31G(d) basis set.[13,14] Further, dispersion interactions were taken into account using Grimme´s empirical dispersion correction (GD3).[15]
The molecular volume inside a contour of 0.001 e^-/a_0^3 density was computed to estimate the Onsager radii for the ground state structures of **NR-13**, **NR-33**, and **NR-53**. As advised, the thereby obtained value was raised by 0.5 Å.[16]

Table S12. Calculated excitation energies (S_1 and T_1) and the energy difference between S_1 and 2*T_1 for **NR-13-H**, **NR-33-H**, and **NR-53-H**, using the B3LYP functional with the 6-31G(d,p) basis set in chloroform (geometries optimized at the B3LYP/6-31G(d,p) level). All values are given in eV.

B3LYP-6-31G(d,p)-Chloroform/B3LYP-6-31G(d,p)							
	T_1	T_1'	T_2	T_2'	S_1	S_2	S_1-2*T_1
**NR13-H**	0.82	0.82	1.69	1.69	1.87	2.01	**0.22***
**NR33-H**	0.82	0.82	1.07	1.07	1.86	1.90	**0.21**
**NR53-H**	0.82	0.82	1.07	1.07	1.86	1.89	**0.21**
M062X-6-31G(d,p)-Chloroform/B3LYP-6-31G(d,p)							
	T_1	T_1'	T_2	T_2'	S_1	S_2	S_1-2*T_1
**NR13-H**	1.04	1.04	2.13	2.25	2.38	2.56	0.30*
**NR33-H**	1.04	1.04	1.38	1.38	2.38	2.51	0.30
**NR53-H**	1.04	1.04	1.38	1.38	2.38	2.49	0.30

* The same calculations with a larger basis set yield better S_1-2*T_1 values: 0.06 eV for **NR-13-H**.

5. References

(1) *CRC Handbook of Chemistry and Physics*, 104th ed.; Rumble, J. R., Ed.; CRC Press, 2023.

(2) Basel, B. S.; Zirzlmeier, J.; Hetzer, C.; Reddy, S. R.; Phelan, B. T.; Krzyaniak, M. D.; Volland, M. K.; Coto, P. B.; Young, R. M.; Clark, T.; Thoss, M.; Tykwinski, R. R.; Wasielewski, M. R.; Guldi, D. M. Evidence for Charge-Transfer Mediation in the Primary Events of Singlet Fission in a Weakly Coupled Pentacene Dimer. *Chem* **2018**, *4* (5), 1092–1111. https://doi.org/10.1016/j.chempr.2018.04.006.

(3) Zoon, P. D.; Brouwer, A. M. A Push-Pull Aromaticchromophore with a Touch of Merocyanine. *Photochem. Photobiol. Sci.* **2009**, *8* (3), 345–353. https://doi.org/10.1039/b818371f.

(4) Bublitz, G. U.; Boxer, S. G. Effective Polarity of Frozen Solvent Glasses in the Vicinity of Dipolar Solutes. *J. Am. Chem. Soc.* **1998**, *120* (16), 3988–3992. https://doi.org/10.1021/ja971665c.

(5) Epelde-Elezcano, N.; Martínez-Martínez, V.; Peña-Cabrera, E.; Gómez-Durán, C. F. A.; Arbeloa, I. L.; Lacombe, S. Modulation of Singlet Oxygen Generation in Halogenated BODIPY Dyes by Substitution at Their Meso Position: Towards a Solvent-Independent Standard in the Vis Region. *RSC Adv.* **2016**, *6* (48), 41991–41998. https://doi.org/10.1039/C6RA05820E.

(6) Spiller, W.; Kliesch, H.; Wöhrle, D.; Hackbarth, S.; Röder, B.; Schnurpfeil, G. Singlet Oxygen Quantum Yields of Different Photosensitizers in Polar Solvents and Micellar Solutions. *J. Porphyr. Phthalocyanines* **1998**, *02* (02), 145–158. https://doi.org/10.1002/(SICI)1099-1409(199803/04)2:2<145::AID-JPP60>3.0.CO;2-2.

(7) Winget, P.; Horn, A. H. C.; Selçuki, C.; Martin, B.; Clark, T. AM1* Parameters for Phosphorus, Sulfur and Chlorine. *J. Mol. Model.* **2003**, *9* (6), 408–414. https://doi.org/10.1007/s00894-003-0156-7.

(8) Kayi, H.; Clark, T. AM1* Parameters for Copper and Zinc. *J. Mol. Model.* **2007**, *13* (9), 965–979. https://doi.org/10.1007/s00894-007-0214-7.

(9) Winget, P.; Clark, T. AM1* Parameters for Aluminum, Silicon, Titanium and Zirconium. *J. Mol. Model.* **2005**, *11* (6), 439–456. https://doi.org/10.1007/s00894-005-0236-y.

(10) Margraf, J. T.; Hennemann, M.; Clark, T. EMPIRE: A Highly Parallel Semiempirical Molecular Orbital Program: 3: Born-Oppenheimer Molecular Dynamics. *J. Mol. Model.* **2020**, *26* (3), 22–25. https://doi.org/10.1007/s00894-020-4293-z.

(11) Margraf, J. T.; Hennemann, M.; Meyer, B.; Clark, T. EMPIRE: A Highly Parallel Semiempirical Molecular Orbital Program: 2: Periodic Boundary Conditions. *J. Mol. Model.* **2015**, *21* (6), 1–7. https://doi.org/10.1007/s00894-015-2692-3.

(12) Hennemann, M.; Clark, T. EMPIRE: A Highly Parallel Semiempirical Molecular Orbital Program: 1: Self-Consistent Field Calculations. *J. Mol. Model.* **2014**, *20* (7), 1–11. https://doi.org/10.1007/s00894-014-2331-4.

(13) Axel, D. B. Density-functional Thermochemistry. III. The Role of Exact Exchange. *J. Chem. Phys.* **1993**, *98* (7), 5648–5652.

(14) Petersson, G. A.; Bennett, A.; Tensfeldt, T. G.; Al-Laham, M. A.; Shirley, W. A.; Mantzaris, J. A Complete Basis Set Model Chemistry. I. The Total Energies of Closed-Shell Atoms and Hydrides of the First-Row Elements. *J. Chem. Phys.* **1988**, *89* (4), 2193–2218. https://doi.org/10.1063/1.455064.

(15) Grimme, S.; Antony, J.; Ehrlich, S.; Krieg, H. A Consistent and Accurate Ab Initio

Parametrization of Density Functional Dispersion Correction (DFT-D) for the 94 Elements H-Pu. *J. Chem. Phys.* **2010**, *132* (15). https://doi.org/10.1063/1.3382344.

(16) Frisch, M. J.; Trucks, G. W.; Schlegel, H. B.; Scuseria, G. E.; Robb, M. A.; Cheeseman, J. R.; Scalmani, G.; Barone, V.; Petersson, G. A.; Nakatsuji, H.; Li, X.; Caricato, M.; Marenich, A. V.; Bloino, J.; Janesko, B. G.; Gomperts, R.; Mennucci, B.; Hratchian, H. P.; Ortiz, J. V.; Izmaylov, A. F.; Sonnenberg, J. L.; Williams-Young, D.; Ding, F.; Lipparini, F.; Egidi, F.; Goings, J.; Peng, B.; Petrone, A.; Henderson, T.; Ranasinghe, D.; Zakrzewski, V. G.; Gao, J.; Rega, N.; Zheng, G.; Liang, W.; Hada, M.; Ehara, M.; Toyota, K.; Fukuda, R.; Hasegawa, J.; Ishida, M.; Nakajima, T.; Honda, Y.; Kitao, O.; Nakai, H.; Vreven, T.; Throssell, K.; Montgomery, J. A., Jr.; Peralta, J. E.; Ogliaro, F.; Bearpark, M. J.; Heyd, J. J.; Brothers, E. N.; Kudin, K. N.; Staroverov, V. N.; Keith, T. A.; Kobayashi, R.; Normand, J.; Raghavachari, K.; Rendell, A. P.; Burant, J. C.; Iyengar, S. S.; Tomasi, J.; Cossi, M.; Millam, J. M.; Klene, M.; Adamo, C.; Cammi, R.; Ochterski, J. W.; Martin, R. L.; Morokuma, K.; Farkas, O.; Foresman, J. B.; Fox, D. J. Gaussian 16, Revision C. 01, Gaussian. *Inc., Wallingford CT* **2016**, *3*.
